# Gene-Based Sequencing Identifies Lipid-Influencing Variants with Ethnicity-Specific Effects in African Americans

**DOI:** 10.1371/journal.pgen.1004190

**Published:** 2014-03-06

**Authors:** Amy R. Bentley, Guanjie Chen, Daniel Shriner, Ayo P. Doumatey, Jie Zhou, Hanxia Huang, James C. Mullikin, Robert W. Blakesley, Nancy F. Hansen, Gerard G. Bouffard, Praveen F. Cherukuri, Baishali Maskeri, Alice C. Young, Adebowale Adeyemo, Charles N. Rotimi

**Affiliations:** 1 Center for Research in Genomics and Global Health, National Human Genome Research Institute, National Institutes of Health, Bethesda, Maryland, United States of America; 2 National Institutes of Health Intramural Sequencing Center, National Human Genome Research Institute, National Institutes of Health, Bethesda, Maryland, United States of America; 3 Genome Technology Branch, National Human Genome Research Institute, National Institutes of Health, Bethesda, Maryland, United States of America; 4 National Human Genome Research Institute, National Institutes of Health, Bethesda, Maryland, United States of America; Institute for Molecular Medicine Finland (FIMM), Finland

## Abstract

Although a considerable proportion of serum lipids loci identified in European ancestry individuals (EA) replicate in African Americans (AA), interethnic differences in the distribution of serum lipids suggest that some genetic determinants differ by ethnicity. We conducted a comprehensive evaluation of five lipid candidate genes to identify variants with ethnicity-specific effects. We sequenced *ABCA1*, *LCAT*, *LPL*, *PON1*, and *SERPINE1* in 48 AA individuals with extreme serum lipid concentrations (high HDLC/low TG or low HDLC/high TG). Identified variants were genotyped in the full population-based sample of AA (n = 1694) and tested for an association with serum lipids. rs328 (*LPL*) and correlated variants were associated with higher HDLC and lower TG. Interestingly, a stronger effect was observed on a “European” vs. “African” genetic background at this locus. To investigate this effect, we evaluated the region among West Africans (WA). For TG, the effect size among WA was the same in AA with only African local ancestry (2–3% lower TG), while the larger association among AA with local European ancestry matched previous reports in EA (10%). For HDLC, there was no association with rs328 in AA with only African local ancestry or in WA, while the association among AA with European local ancestry was much greater than what has been observed for EA (15 vs. ∼5 mg/dl), suggesting an interaction with an environmental or genetic factor that differs by ethnicity. Beyond this ancestry effect, the importance of African ancestry-focused, sequence-based work was also highlighted by serum lipid associations of variants that were in higher frequency (or present only) among those of African ancestry. By beginning our study with the sequence variation present in AA individuals, investigating local ancestry effects, and seeking replication in WA, we were able to comprehensively evaluate the role of a set of candidate genes in serum lipids in AA.

## Author Summary

Most of the work on the genetic epidemiology of serum lipids in African Americans (AA) has focused on replicating findings that were identified in European ancestry individuals. While this can be very informative about the generalizability of lipids loci across populations, African ancestry-specific variation will be missed using this approach. Our aim was to comprehensively evaluate five lipid candidate genes in an AA population, from the identification of variants of interest to population-level analysis of high-density lipoprotein cholesterol (HDLC) and triglycerides (TG). We sequenced five genes in individuals with extreme lipids (n = 48) drawn from a population-based study of AA. The variants identified were genotyped in 1,694 AA and analyzed. Notable among the findings were the observation of ancestry specific effect for several variants in the *LPL* gene among these admixed individuals, with a greater effect observed among those with European ancestry in this region. These associations were further elucidated by replication in West Africans. By beginning with the sequence variation present among AA, investigating ancestry effects, and seeking replication in West Africans, we were able to comprehensively evaluate these candidate genes with a focus on African ancestry individuals.

## Introduction

The role of the distribution of serum lipids in influencing disease risk is well-established. Serum lipids are under the influence of genetic and non-genetic (e.g., dietary) factors. Lipids are routinely evaluated in the screening for and monitoring of metabolic disorders. Effectively controlling serum lipids is a key intervention for metabolic disorders, providing a compelling motivation for investigating the genetic determinants of these traits, as new understanding of biology and potential drug targets can be achieved using this approach. Heritability estimates for these traits suggest that they are highly heritable, with a range of 43–76% for high-density lipoprotein cholesterol (HDLC) and 28–71% for triglycerides (TG) among those of European ancestry [1]–[6] (with overlapping estimates among African ancestry individuals [6]–[9]). While large-scale efforts have made considerable progress in identifying genetic factors underlying the distribution of serum lipids (for instance [10]), the focus of the majority of reports of the genetic epidemiology of serum lipids in diverse populations has been on replication or fine-mapping of variants that were identified in European ancestry individuals [10]–[15]. Although agreement between findings in samples of different ancestries does provide support for the significance of specific variants, this approach can only give a limited understanding of the genetic factors that influence trait distribution in diverse populations as it ignores variation of importance in the replication sample that would not be identified in the initial sample (due to interethnic frequency differences, for example).

The existence of interethnic differences in distribution of serum lipids between African Americans (AA) and individuals of European ancestry is known [16]. AA individuals generally have healthier lipid profiles than those of non-African ancestry, counter to expectation based on distributions of lifestyle factors that influence serum lipids. In nationally-representative data, for instance, mean serum triglycerides were 113 mg/dl in AA and 143 mg/dl in European Americans (EA), and high-density lipoprotein cholesterol (HDLC) was higher in AA compared to EA (54 vs. 50 mg/dl) [12]. The fact that these differences are seen in children [17]–[19] and that low TG has also been observed among those of similar genetic ancestry but widely divergent environments (for instance, among African Americans and West Africans [16]) provide strong evidence for a role of genetic factors. Further support for this inference comes from the observation that HDLC level increases with increasing proportion of genome-wide African ancestry in AA; this proportion is associated inversely with TG [16], [20]. Taken together, these observations suggest the contribution of genetic variation that is highly differentiated or not shared between populations in influencing serum lipids, motivating African-ancestry focused analyses.

Although there are many genes that have been associated with serum lipids that could have been selected for this study, we focused on the following 5 genes because of their potential (based on literature review) to provide novel insights into the well-documented differences between lipid profile of EA and AA: *ATP-binding cassette A1* (*ABCA1*), *lecithin-cholesterol acyltransferase* (*LCAT*), *lipoprotein lipase* (*LPL*), *paraoxonase 1* (*PON1*), *serpin peptidase inhibitor E1* (*SERPINE1*). ABCA1 is a membrane-associated protein that is central to reverse cholesterol transport, acting as an efflux pump to facilitate the removal of cellular lipid to apolipoprotein A-I. Sequence variants in *ABCA1* have consistently been associated with HDLC concentration [10], [12], [15], and variations in this gene lead to Tangier disease, defined by extremely low levels of HDLC [21]. LCAT converts free cholesterol into cholesterol ester, a key step in the formation of HDL, and sequence variants in *LCAT* are associated with HDLC concentration [10], [12], [15], [22], [23]. LPL hydrolyzes TG and releases fatty acids. Sequence variants in *LPL* are associated with TG [10], [12], [15], [20], [22] and HDLC [10], [12], [15]. PON1 hydrolyzes a wide range of substrates and protects against lipid oxidation, being largely responsible for the antioxidative properties of the HDL particle. *PON1* is of particular interest based on a protective role for atherosclerosis and related outcomes (reviewed in [24]). Additionally, a linkage analysis for HDLC in West Africans identified a region that includes *PON1* [7]. *SERPINE1* encodes plasminogen activator inhibitor-1, an important regulator of fibrinolysis. PAI-1 concentration is associated with both CVD [25] and Metabolic Syndrome [26], and sequence variation in *SERPINE1* is associated with TG [27], [28].

We sought to take a comprehensive, in-depth look at the association between these selected lipid candidate genes and serum lipids in African Americans. We sequenced these genes in 48 individuals from the extremes of the distribution of serum lipids in a population-based sample and genotyped the identified variants in the full cohort (n = 1694). The association between these variants and serum lipids was evaluated using separate rare and common variant analyses. We were able to identify lipids-associated variants that were African ancestry-specific (or at much higher frequency in African ancestry) and variants with a different effect depending on ancestry.

## Results

Characteristics of AA participants included in all stages of this study are displayed in Table 1. By design, the differences in lipid parameters are striking between those individuals who were selected for sequencing due to extreme lipid values. It is still notable, however, that the difference in mean TG between groups is 100 mg/dl, given that these participants came from a population-based sample of AA that was not selected for any extreme phenotype. In this study, participants with a favorable lipid profile were more likely to be women and leaner. Among the full sample of genotyped individuals, mean TG is quite low, as is consistently observed in African-ancestry populations. As expected, HDLC and TG were inversely correlated (Pearson correlation coefficient −0.3, p<0.0001). West Africans (WA) included in the replication analysis were somewhat older (mean 47.7 years), with lower HDLC (mean 39.3 mg/dl) and similar TG (mean 75.5 mg/dl; Table S1).

**Table 1:** Participant characteristics^1^.

	Sequencing Stage		Genotyping Stage
	Favorable Profile	Unfavorable Profile	
N	24	24	1694
Age	39.8 (±11.1)	42.1 (±8.8)	37.7 (±16.3)
% Women	71%	42%	61%
BMI	25.0 (±4.6)	37.3 (±8.5)	29.4 (±8.3)
HDLC	70.1 (±4.6)	33.0 (±1.3)	53.0 (±14.5)
TG^2^	47.0 (±5.0)	147.0 (±34.0)	77.0 (±51.0)

*Abbreviations: Body Mass Index (BMI); ^1^Mean (± standard deviation) given except where indicated; ^2^median and interquartile range given due to non-normality.*

### Sequencing Stage

We sequenced 98,901 base pairs across selected regions of the five candidate genes (including known or predicted exons, flanking introns, 5′ untranslated region (UTR)/promoter region, and evolutionarily conserved regions of each gene), distributed as follows: *ABCA1* 64,516; *LCAT* 4,432; *LPL* 10,131; *PON1* 9,999; and *SERPINE1* 9,823. The frequency and type of SNPs discovered in this sequencing stage were not different from the distribution of variants identified in the same regions in the 1000 Genomes data (AFR)[29] when data was limited to variants with MAF≥0.01 (our power to detect variants with a MAF<0.01 was less than 60% given the number of chromosomes interrogated [n = 96]). Given that our interest was in describing all the variation in these genes, including variants that might not have been detected in our sequencing stage, the 675 variants identified were supplemented with imputation using 1000 Genomes data as reference [29] for a total of 1,918 variants carried forward for genotyping in the larger cohort. There were 110 variants that were only present among those in the extreme group with a favorable lipid profile and 115 variants that were only found in the extreme group with an unfavorable lipid profile (Table S2). Of these variants that were exclusive to one extreme group that were not successfully genotyped or imputed for follow-up in the full study population, there were two of note. rs268 in *LPL* has been previously associated with serum lipids and related disease outcomes [30]–[32]; this variant was excluded from analysis in the full study population because it was not in HWE. *ABCA1* variant rs35819696 was previously identified in AA from the Dallas Heart Study who had low HDLC [33]; this variant was found only in the unfavorable lipid profile group, but it was monomorphic in the full study population, precluding further analysis.

### Genotyping Stage

Of the 1,918 variants identified in the sequencing and imputation stages, 1,415 were successfully genotyped or imputed in the full sample of 1,694 individuals. In the rare variant (RV) analysis, one association was found between gene-defined SNP set and serum lipids that remained statistically significant after correction for multiple comparisons. RVs in *ABCA1* were associated with logTG (p = 0.0095). No associations were observed for any of the other genes with logTG or for any of the genes with HDLC in the RV analysis. Haplotype analyses were conducted by gene but no haplotypes were statistically significant after permutation testing. Statistically significant results from the common variant (CV) analysis, after correction for multiple hypothesis testing, are presented in Table 2 and reviewed below by gene. All effects are described in terms of the minor allele.

**Table 2:** Common variants in candidate genes associated with serum lipids in African Americans (n = 1694).

Trait	Gene	Variant(s)	Chr	Position^1^	MAF	Ref/Alt	Coding	β^2^ (95% CI)	p-value^3^
*HDLC*	*ABCA1*	rs2740477	9	107564077	0.36	G/A	Het	−2.2 (−3.5, −0.9)	0.02^5^
		rs199734367	9	107570148	0.50	CACACACACAT/C	Dom	−2.5 (−4.1, −1.0)	0.03^5^
		rs78294949	9	107605638	0.05	G/T	Add	4.0 (1.8, 6.2)	0.009
		rs114851717^4^	9	107606009	0.06	G/C	Add	4.0 (2.0, 6.1)	0.003
		rs115763221	9	107611337	0.10	G/T	Dom	2.8 (1.1, 4.5)	0.03^5^
		rs73521828	9	107613849	0.08	T/A	Het	3.2 (1.4, 5.0)	0.01^5^
	*LCAT*	rs13306496	16	67976692	0.12	G/T	Rec	−6.5 (−11.9, −1.1)	0.03^5^
	*LPL*	rs256^4^	8	19811967	0.07	C/T	Rec	14.7 (5.9, 23.5)	0.008
		rs328^4^	8	19819724	0.07	C/G	Rec	15.2 (6.9, 23.5)	0.002
		rs201109344	8	19821465	0.09	TG/T	Add	2.7 (1.0, 4.4)	0.01
		rs1059611^4^	8	19824563	0.18	T/C	Add	1.7 (0.5, 2.9)	0.04^5^
	*PON1*	rs2049649	7	94949329	0.42	A/G	Dom	−2.0 (−3.4, −0.6)	0.03^5^
*LogTG*	*ABCA1*	rs2515602^4^	9	107600168	0.27	A/G	Het	−3.2% (−5.2, −1.5)	0.009
		rs2472449	9	107604197	0.24	G/T	Het	−3.2% (−5.1, −1.3)	0.03^5^
		rs58219156	9	107604722	0.20	A/C	Rec	8.6% (3.4,13.8)	0.03^5^
		rs35699471	9	107650820	0.08	C/T	Het	−4.8% (−7.5, −2.0)	0.02^5^
		rs1800977^4^	9	107690450	0.26	G/A	Dom	−3.7% (−5.6, −1.8)	0.003
	*LPL*	rs74304285	8	19808030	0.08	G/A	Het	−4.3% (−7.0, −1.7)	0.01
		rs328^4^	8	19819724	0.07	C/G	Add	−5.1% (−7.5, −2.6)	0.0001
		rs12679834	8	19820433	0.09	T/C	Add	−5.4% (−7.8, −3.0)	0.00009
	*PON1*	rs3917549	7	94935198	0.48	CA/A	Het	−2.7% (−4.5, −0.9)	0.03^5^
	*SERPINE1*	rs2227674	7	100776208	0.27	A/G	Het	−2.5% (−4.4, −0.6)	0.03^5^

Abbreviations: Additive (Add), Chromosome (Chr), Dominant (Dom), Minor Allele Frequency (MAF), High-density Lipoprotein Cholesterol (HDLC), Recessive (Rec), Log-transformed Triglycerides (logTG); ^1^Build 37 position; ^2^ βs are given for the minor (“Alt”) allele. TG values were log-transformed: β values represent the percent change in TG. ^3^Correction for multiple hypotheses conducted by calculating the effective degrees of freedom using the covariance matrix for variants in each gene. All P-values have been multiplied by the effective degrees of freedom for that gene (see methods and reference[54]); ^4^Similar results observed for the following CVs in high LD (R^2^>0.8) with listed SNP: rs114851717 (rs138618449, rs148714575, rs115761095, rs76729624), rs256 (rs271), rs328 (rs325, rs117199990, rs145391587, rs75278536, rs77069344, rs11570891, rs1803924, rs3735964), rs1059611 (rs149865365), rs2515602 (rs2472386), rs1800977 (rs2437817, rs2243312).CVs in LD (R^2^>0.6); ^5^These associations would no longer be statistically significant if adjusted for model selection (4 genetic models compared).

CVs in *LPL* were associated with serum lipids. The well-known missense variant rs328 and variants that are in linkage disequilibrium (LD) with it were associated with increased HDLC and decreased logTG. Across this region, individuals who were homozygous for the risk allele had ∼15 mg/dl higher HDLC compared to those with other genotypes (p = 0.0002 for rs328), while each minor allele was associated with a ∼5% decrease in TG (p = 0.0001 for rs328). A variant in LD (R^2^ = 0.6), rs12679834, was also associated with logTG (p = 0.00009), but not HDLC (p = 0.2).

**Figure 1:**
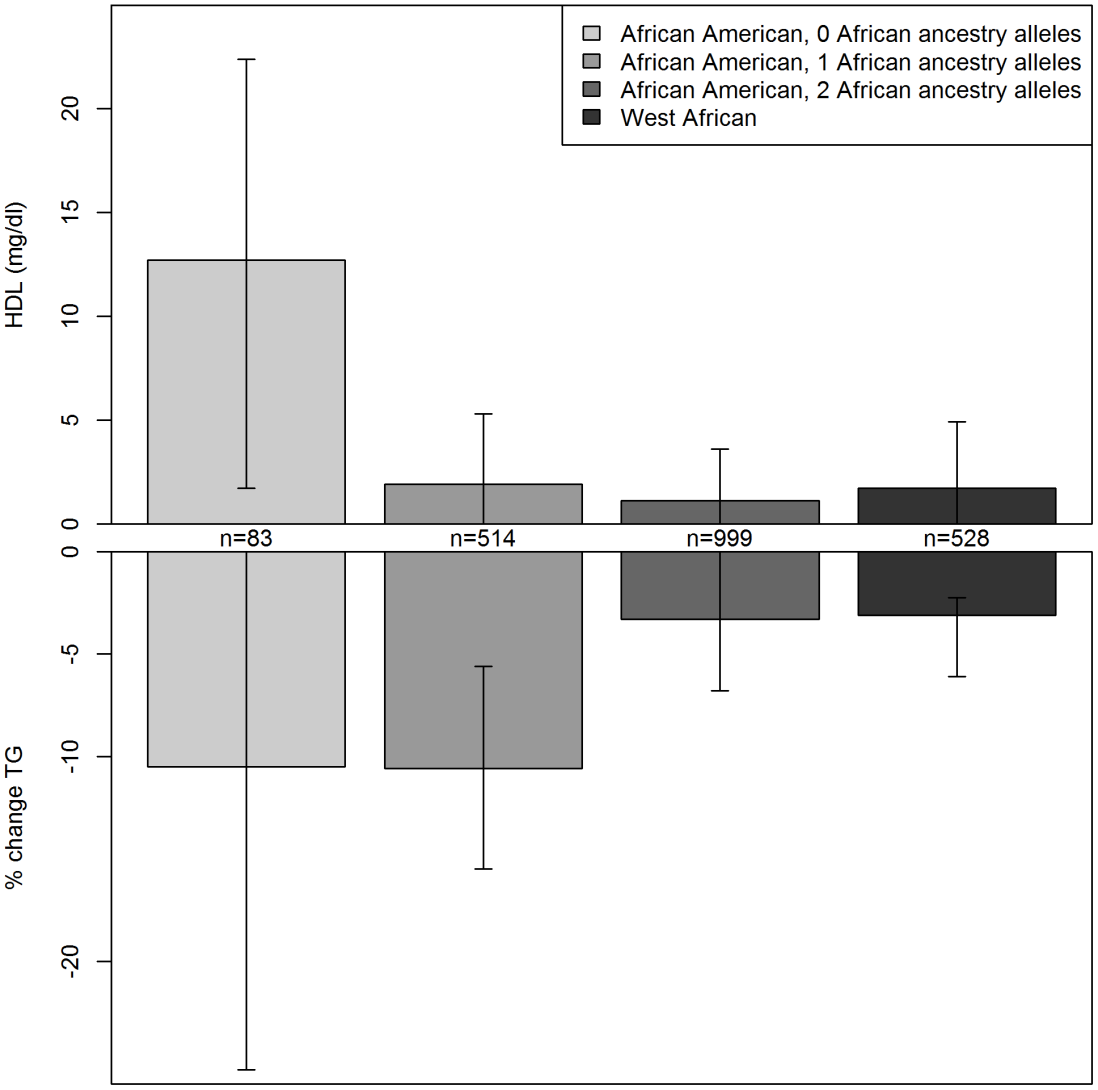
Serum lipids by rs328 genotype and local ancestry in African Americans and West Africans. Difference in serum HDLC and logTG with the variant genotype for African Americans with 0, 1, or 2 African ancestry alleles at this locus, and West African individuals. Results for other *LPL* variants in LD with rs328 were similar.

For further understanding of the association between variants in this region and serum lipids, the influence of local ancestry on the reported associations was evaluated. As with all analyses in this study, models were adjusted for genome-wide average proportion of African ancestry; thus, local ancestry effects observed should not be confounded by genome-wide African ancestry. Nearly all of the associations in this region were significantly modified by local ancestry (Table S3). As illustrated for rs328 (Figure 1), the variant was associated with a larger effect size on the European- compared to the African-ancestry background in this admixed sample of AA. Statistically significant interactions were observed between rs328 carrier status and local ancestry for both HDLC (p_interaction_ = 0.008) and logTG (p_interaction_ = 0.01). Notably, among AA with only European ancestry at this locus, carriers of the minor allele had 12.7 mg/dl higher HDLC than those homozygous for the major allele (p = 0.02); among those with African ancestry at this locus, rs328 was not associated with HDLC (difference between carriers and non-carriers of the minor allele <2 mg/dl). Similarly, among those with either 1 or 2 copies of the European ancestry allele at this locus, rs328 carriers of the minor allele had 10.5% lower TG levels than non-carriers (p = 0.00002), while this difference was reduced substantially to 3.3% (p = 0.06) among those with two African-ancestry alleles. As expected, genome-wide proportion of African ancestry varied within category of local ancestry: mean genome-wide African ancestry was 68%, 75%, and 82% among those with 0, 1, and 2 copies of the African ancestry allele, respectively.

There was evidence for replication of the association between some of these variants and logTG among WA. The association between rs328 and logTG was much smaller and not statistically significant among the WA: the minor allele was associated with 2% lower TG (p = 0.3). There was insufficient power to evaluate the rs328-HDLC relationship in WA. Nearby variant rs12679834 (R^2^ with rs328 = 0.81) was associated with a 5% lower TG in WA (p = 0.04). The stronger association with rs12679834 may result from the slightly higher MAF of this variant compared to rs328 (0.09 vs. 0.07). Of note is the consistency of the ancestry effects for both HDLC and logTG: the association observed for these *LPL* variants among WA matched very closely with what was observed among the admixed AA with primarily African ancestry at this locus (Figure 1).

Another *LPL* variant, rs1059611 (and rs149865365, R^2^ = 0.99 with rs1059611), which was not in LD with the other *LPL* variants (R^2^<0.3 for all comparisons), was associated with 2 mg/dl higher HDLC; there is also evidence of significant modification of this association by local ancestry (p_interaction_ = 0.004). As above with rs328, greater differences in HDLC by minor allele carrier status were observed on the European ancestry background compared to the African ancestry background (14.6 for 2 copies of the European ancestry allele vs. <2 mg/dl with any copy of the African ancestry allele). Despite reasonable power (0.74) to detect an association of the magnitude observed in AA among WA, the rs1059611 finding did not replicate (−0.6 mg/dl, p = 0.6); this result is consistent with the observed local ancestry effect (with association in AA seen only on the European ancestry background).

Several intronic CVs in *ABCA1* that are predicted to affect a variety of regulatory motifs [34] were associated with altered serum lipids (Table 2). Four of these CVs are in much higher frequency among African ancestry individuals, with rs78294949, rs114851717, and rs73521828 only found among those with African ancestry, and clear differentiation by ethnicity for rs115763221 (1000 Genomes [29] MAF: 0.17 (AFR), 0.003 (EUR)).

*LCAT* variant rs13306496 was associated with 5 mg/dl lower HDLC. This intronic variant does not alter LCAT protein sequence, but it is predicted to alter regulatory motifs (HaploReg [34]). This variant is present at a higher frequency among African-ancestry populations compared to other ethnicities (1000 Genomes [29]): MAF 0.16 (AFR) and 0 (EUR). Variants in this gene were not associated with TG.

In *PON1*, two CVs were associated with serum lipids. A common *PON1* variant, rs2049649, was associated with HDLC. This intronic variant alters regulatory motifs and promoters [34]. A small deletion, rs3917549, was associated with lower logTG. rs3917549 alters regulatory motifs and is in much higher frequency among African ancestry vs. European ancestry populations (AFR: 0.62, EUR: 0.18).

For *SERPINE1*, one CV was associated with serum lipids. *SERPINE1* CV rs2227674 was inversely associated with logTG. This variant has been previously associated with plasminogen activator inhibitor-1 levels [35].

## Discussion

In this study, we undertook a comprehensive evaluation of the sequence variation present in 5 serum lipids candidate genes and their association with HDLC and TG to shed light on the consistently observed, yet unexplained, different lipid profiles seen in African Americans (AA) compared to European Americans (EA). Genes *ABCA1*, *LCAT*, *LPL*, *PON1*, and *SERPINE1* were each sequenced in 48 AA individuals, and the variants identified were genotyped in a population-based sample of AA. Using a variety of analytical techniques, we were able to describe the genetic architecture of these traits at these loci in AA. Notable among our findings, in terms of underscoring the importance of African ancestry-focused studies of serum lipids, are the discovery of lipids-associated variants that are in higher frequency (or only found) among African ancestry individuals, loci for which effect size differed by genetic ancestry in this admixed population, and an opportunity to take advantage of interethnic LD differences.

One of the motivations for conducting population-specific work is that some risk variants may be absent or at different frequencies by ethnicity. For example, variants of minimal impact among European ancestry individuals may have a greater significance in AA because of a higher frequency. Of those variants that were analyzed in this study, all of the *LCAT* variants were in significantly higher frequency (χ^2^ test, p<0.05) among the 1000 Genomes African vs. European ancestry samples (AFR vs. EUR); for the other genes, the majority of variants followed this pattern (*ABCA1* 74%, *LPL* 70%, *PON1* 64%, *SERPINE1* 52%). Notably higher minor allele frequencies, among 1000 Genomes African vs. European ancestry samples (AFR vs. EUR), were observed for some of the associated CVs including *ABCA1* variant rs115763221 (AFR: 0.17, EUR: 0.003, p<0.0001), *LCAT* variant rs13306496 (AFR: 0.16; EUR: 0.003, p<0.0001), and *LPL* variant rs201109344 (AFR: 0.08, EUR: 0.001, p<0.0001). Three (rs78294949, rs114851717, and rs73521828) of the associated *ABCA1* CVs were not present in the 1000 Genomes European ancestry samples. Many of the RVs in this study were African ancestry specific, and 65% of the RVs analyzed were found in AFR and not in EUR. Clearly, these associations could not be evaluated in a non-African ancestry sample.

Interethnic differences in the genetic architecture of serum lipid traits extend beyond simple frequency differences as highlighted with this study's observation of variants with differing effect sizes by local ancestral background. A common *LPL* nonsense variant, rs328 (S447X), and variants in LD with rs328 were associated with both HDLC and TG in this study. This variant is associated with increased *LPL* mRNA [36], increased LPL activity [37], decreased TG [20], [38]–[40], and increased HDLC [41]–[44]. In this study of admixed AA, much larger associations were observed among those with European ancestry at this locus as compared to those with predominantly African ancestry at this locus, as has been previously observed [20]. The local ancestry-stratified logTG outcomes in AA are remarkably consistent with observations in the respective parental populations: the size of the observed association was 2–3% in AA with only African local ancestry and WA, while the larger effect size among AA with local European ancestry (10.5%) is nearly identical to what was reported in a meta-analysis of over 43,000 predominantly European ancestry individuals (10%) [45]. The local ancestry-stratified HDLC outcomes also show a larger effect size with local European ancestry, but the comparisons with parental populations are more complex. While the effect size among AA with local African ancestry was consistent with what was seen in WA, the effect size among AA with local European ancestry was more than twice what was observed in those with European ancestry (12.7 vs. ∼5 mg/dl higher for GG vs. CC) [41]–[44]. Thus, this variant is showing a larger association in AA than has been previously reported for European ancestry populations, even when limiting to AA individuals with local European ancestry. This inconsistency suggests the presence of a genetic or environmental factor that influences the rs328-HDLC association and differs by ethnicity. Effect modification of the rs328-HDLC association has been observed with dietary fat parameters [39], [44]. Intriguingly, in one analysis, an interaction that differed by ethnicity was observed for HDLC, and a similar interaction was not observed for TG, in agreement with our TG findings, which were consistent within genetic ancestry categories.

The generally reduced LD in the genomes of African ancestry individuals compared to European ancestry individuals can be used to narrow the region of interest around an association signal (trans-ethnic fine-mapping). Similar associations have been observed among those of European and Asian ancestry for rs1059611 and rs328 with serum lipids [39], [46], [47]; this similarity is unsurprising given the high LD between these variants among these populations (R^2^ = 0.96). In this study of AA, however, these variants were not in LD (R^2^ = 0.28), the rs328-HDLC effect size was nearly 9-fold larger than the rs1059611-HDLC effect size, and no association was observed for rs1059611-logTG.

It is expected that interethnic differences in relevant genetic variants may contribute to the observed differences in the distribution of serum lipids in African ancestry individuals compared to those of other ethnicity (with more favorable lipid profiles, higher HDLC and lower TG among AA). The most compelling case to be made for a genetic contribution to the interethnic serum lipid differences may be with rs328 (and variants in LD with rs328) and HDLC. These variants are associated with a favorable lipid profile, but the associated variants in this region are consistently in ∼5% higher minor allele frequency among EA than AA, and less common among WA. However, for HDLC, the effect size in AA was 15 mg/dl for variants in this region, while among EA the effect size is 4–6 mg/dl. Interestingly, though TG is consistently low across African ancestry populations, HDLC is generally much higher among AA than WA (mean HDLC: AA 53.0, WA 39.3 mg/dl). This HDLC distribution is consistent with the possibility of variants from European ancestry influencing HDLC among AA, but with a larger effect in AA, perhaps due to some gene × environment interaction (as has been reported for rs328 [39], [44]).

A similar approach to ours was undertaken in the Dallas Heart Study [33]. In this study, Cohen et al found that nonsynonymous sequence variants were more common among individuals in the lowest vs. the highest 5% of the distribution of HDLC in their population-based study. While we did not find an excess of nonsynonymous variants in those sequenced compared to what was found in the 1000 Genomes data, one of the variants identified among African Americans in their low HDLC group was also observed in our unfavorable lipid profile group, providing further support for the role of *ABCA1* variant rs35819696 in influencing serum lipids.

Some strengths of this study deserve mention. First, the fact that this project began with sequencing AA at the extremes of the lipid distribution is significant. Selecting variants in this way, as opposed to simply trying to assess the replication of findings identified in other populations, allowed us to address the question of what variation influences these traits in African-ancestry individuals instead of evaluating how similar results are across ethnicities. This distinction is important given the evidence that the distribution of serum lipids differs by ancestry and the evidence of a different relationship between serum lipids and metabolic disorders (reviewed in [16]). Additionally, with the increasing emphasis in complex disease research on rare variation, which is less shared across ethnicities than common variation, beginning with variants identified directly in an AA sample is appropriate. Another strength of this study was the inclusion of supporting information from WA individuals. Given the genetic similarity between these populations (∼80% genome-wide shared ancestry with AA) and the widely divergent lifestyle, diet, and environmental contexts between them, such comparisons are invaluable in disentangling the basis of complex traits in admixed individuals. In this analysis, these data were particularly informative for evaluating the loci for which there was a different association by local ancestry.

This study had a few limitations which should be considered. The selection of these candidate genes on which to focus our study is certainly insufficient to describe all regions for which genetic factors may play a role in the interethnic differences in serum lipids and it was not our intention to do so. As we sequenced 48 individuals (96 chromosomes) and excluded variants that were only observed once in these individuals, it is expected that the variants identified do not fully describe the variation present, although a serious effort to address this lack was made by supplementing our lab-generated data with variants imputed using the 1000 Genomes samples as a reference [29]. Additionally, the number of WA samples that matched our inclusion criteria and could be used for replication was relatively limited, precluding comprehensive evaluation in WA for the majority of variants that were associated among the AA.

The lack of ethnic diversity in genetic research has come to prominence, with large-scale efforts to reduce this disparity underway (for example, the Human Heredity and Health in Africa [H3Africa] initiative, http://www.h3africa.org/). In this analysis, we targeted the genetic architecture of specific candidate genes, focusing on the variation that was identified directly in an AA sample, yielding useful insights into the interethnic differences in the genetic determinants of these traits. Particularly informative was an association between the *LPL* locus and serum lipids that differed by local genetic ancestry, with data from parental populations supporting inferences and demonstrating the complexity of ancestry effects. African ancestry-focused work is an important part of understanding the role of genetics in the distribution of serum lipids.

## Methods

### Study Population

Participants included in these analyses were from the Howard University Family Study (HUFS), which has been described in detail previously [48]. Briefly, the HUFS followed a population-based selection strategy designed to be representative of African American families living in the Washington, DC metropolitan area. Ethnicity was ascertained by self-report. The HUFS was approved by the Howard University Institutional Review Board, and was conducted in accordance with the Declaration of Helsinki. All participants provided written informed consent.

There were 1694 individuals included in the study after application of exclusion criteria: extreme phenotype values (HDLC<20 or >100 mg/dl; TG<20 or >500 mg/dl) and missing covariate data (age, body mass index [BMI], and gender). Given known perturbations to serum lipids that co-occur with Type 2 Diabetes, subjects with fasting blood glucose ≥126 mg/dl or taking physician-prescribed diabetes medication were excluded. These data include related participants. Family members were included in the common variant analysis, where adjustment for the random effect of family was possible (further description below). For the RV analysis, only unrelated family members were included (with one randomly-selected individual from each family, total n = 919).

### Serum Lipid Measurements

Serum measurements were made on fasting samples. HDLC and TG were determined enzymatically with the COBAS Integra 400 Plus Analyzer (Roche Diagnostics, Indianapolis, IN). Methods were standardized to in-house and other appropriate reference methods: CDC reference methods for HDLC, Isotope dilution-mass spectrometry (ID-MS) for TG by the manufacturer.

### Candidate Gene Sequencing

Forty-eight unrelated subjects were selected from the HUFS, with 24 from the “favorable lipid profile” category (lowest TG quartile, highest HDLC quartile) and 24 from the “unfavorable lipid profile” category (highest TG quartile, lowest HDLC quartile). A sample of 48 individuals (96 chromosomes) provides a 99% probability of finding a sequence variant with a minor allele frequency (MAF) of 0.05, 86% probability of finding a variant with an MAF of 0.02 and 62% probability of finding a variant with an MAF of 0.01. The sequencing strategy applied methods as previously described [49]. Briefly, we sequenced known or predicted exons (including 5 bp of the flanking introns), ∼1 kb of the 5′ untranslated region (UTR)/promoter region, and the 3′ UTR if it is evolutionarily conserved. We also sequenced up to three of the most evolutionarily conserved regions of each gene that are not captured by the above features. Sequencing primers were designed to allow for sufficient overlap in individual sequencing reads. Florescent dye-terminator chemistry was used for bi-directional DNA sequencing, and sequence delineation was performed by automated ABI Prism 3730xl DNA sequencers, which typically give >650 bp Q20/Phred20 read lengths. Mutations and heterozygotes were scored by automated comparative analysis against the provided reference sequence. All mutations were confirmed by manual curation.

### Genotyping and Imputation

Sequence variants were selected based on frequency ≥2% (to exclude variants that were only observed once, which may reflect error), for genotyping in the full HUFS sample. 174 of these identified variants were available from previous genotyping in this sample using the Affymetrix Genome-Wide Human SNP Array 6.0 [48]. Primers were designed for an additional 103 SNPs (Table S4), and genotyping was performed using the iPLEX Gold assay on the MassArray platform (Sequenom, San Diego, CA) as previously described [50]. Briefly, the PCR and extension primers were designed using MassArray designer Software. SNPs were excluded for assay failure (n = 19), lack of variation (n = 13), and genotype success rate <90% (n = 24). After these exclusions, none of the variants failed the filter for departure from Hardy-Weinberg equilibrium (p-value<0.000001).

As our goal was to analyze the variation present in these genes in the individuals with extreme lipid profiles as comprehensively as possible, the list of variants identified by sequencing strategies was augmented by variants identified for these individuals from previous GWAS and by imputation based on 1000 Genomes dataset. Imputation was performed using MaCH-Admix [51], an imputation tool specifically designed for use in admixed samples, using a cosmopolitan reference panel based on 1000 Genomes data. Imputed variants were filtered on an R^2^ of 0.3. Rare variants with a MAF<0.01 were excluded due to diminished confidence in imputation in variants below this threshold.

With previous (n = 174) and new (n = 47) genotyping, and imputation (n = 1,194), there were a total of 1,415 variants were analyzed (Table S5). These included 921 common variants (CVs; MAF ≥ 0.05) and 494 less common or rare variants (RVs; MAF < 0.05) distributed as follows within the candidate genes: *ABCA1* (636 CVs, 389 RVs), *LCAT* (6 CVs, 2 RV), *LPL* (139 CVs, 48 RVs), *PON1* (110 CVs, 33 RVs), and *SERPINE1* (30 CVs, 22 RVs).

### Statistical Analysis

TG was log-transformed, but the distribution of HDLC was approximately normal and left untransformed. Principal component analysis to assess population structure in the admixed African Americans was conducted using EIGENSOFT [52], and, as reported previously [53], the first principal component was retained on the basis of Velicer's minimum average partial test, and included in all analyses as a covariate representing overall proportion of African ancestry. All analyses were also adjusted for age, body mass index (BMI), and gender, and P<0.05 after multiple test correction was considered statistically significant. All SNP effects are described in terms of the minor allele (thus, an “inverse association” indicates that the minor allele was associated with decreasing phenotypic values relative to the major allele).

Separate analytical strategies were employed for the analysis of common variants (CVs) and rare variants (RVs). Variants with MAF ≥ 0.05 were included in the common variant (CV) analysis. For the CV analysis, the associations between variants and phenotypes were assessed in linear mixed models (Proc Mixed) in SAS 9.3 (SAS Institute, Cary, NC) with adjustment for age, BMI, gender, and the overall proportion African ancestry, as well as random clustering within families. For each variant, models were run for additive, dominant, recessive, and heterosis coding. To correct for the number of SNPs tested, P-values were adjusted for the effective number of SNPs (based on LD>0.6) within the gene evaluated, as previously described [54]. Briefly, this method involves conducting a covariance matrix for all of the variants within a gene, using this covariance to determine the LD-adjusted number of independent tests that were conducted when analyzing all of the variants in that gene, and multiplying the individual P-values by that correction factor.

All variants with MAF<0.05 were included in the less common and rare variant analysis (hereafter referred to as the rare variant [RV] analysis). For the RV analysis, SKAT was used (http://www.hsph.harvard.edu/skat/) [55]. SKAT aggregates individual score tests statistics for a set of SNPs, returning a P-value for the set (in our implementation, each gene). Only unrelated participants were included (all unrelated and one randomly-selected individual from each family, n = 919). SKAT accommodates adjustment for covariates, and all analyses were adjusted (as in the CV analysis) for age, BMI, gender, and overall proportion African ancestry. Bootstrap resampling under the null model (considering covariates) was conducted, and statistical significance for the RV analysis was declared after correction for a family-wise error rate of 0.05.

For follow-up in the *LPL* region, local ancestry at the locus was estimated as previously described [56]. Briefly, ancestry at each locus was categorized as having 0, 1, or 2 chromosomes of African ancestry as estimated based on nearly 800,000 markers using LAMPANC version 2.3 [57] and HapMap Phase II+III CEU and YRI reference allele frequencies (http://hapmap.ncbi.nlm.nih.gov/downloads/frequencies/2010-08_phaseII+III/). A difference in genotype-phenotype association by local ancestry was evaluated in SAS using linear mixed models (PROC MIXED) with a genotype by local ancestry interaction term and evaluating models stratified by local ancestry.

Haplotype analysis was conducted in all candidate genes using a sliding window approach in PLINK [58], with up to 5 SNPs included in each haplotype and adjustment for covariates (age, gender, BMI, and overall proportion African ancestry). Permutation testing (1000 permutations) was employed to evaluate statistical significance.

### Replication

Identified loci in African Americans were assessed for replication in a West African sample obtained from the African American Diabetes Mellitus study (AADM; described previously [59]). Briefly, AADM is a large-scale case-control study designed to explore the genetic and environmental determinants of T2D from West Africa, but only non-diabetic controls were included in this analysis. All participants provided written, informed consent. Variants were genotyped using the Affymetrix Axiom Genome-Wide Pan-African Array Set (∼2.2 million markers), which is optimized for coverage of African-ancestry populations. Imputation was also conducted in this sample (as described above). A limited number of participants with genotype data remained after applying the exclusions described above (n≤536). This sample had 80% power to detect an effect of 7% for logTG and 5 mg/dl for HDLC when the variant was common (MAF = 0.05), and 80% power to detect effects of 15% and 11 mg/dl when the variant was less common (MAF = 0.01). The variants for which there was at least moderate power (>60%, QUANTO [60]) to detect an association of the magnitude observed in African Americans are described in the text.

## Supporting Information

Table S1 Participant characteristics, Africa America Diabetes Mellitus Study. Characteristics of West African participants from the Africa America Diabetes Mellitus Study evaluated for replication.
(DOCX)

Table S2 Variants found exclusively in one of the sequencing extremes. Variants that were exclusively found in either the Favorable or Unfavorable Lipid Group during the Sequencing Stage.
(DOCX)

Table S3 Local ancestry interactions among serum lipids-associated LPL variants. Associations between Serum Lipids-associated *LPL* variants within Local Ancestry Strata.
(DOCX)

Table S4 Primers used. Further details on the set of primers used for novel genotyping of variants discovered in the sequencing stage.
(DOCX)

Table S5 Description of included variants. Description of all variants included in the genotyping stage of analysis.
(DOCX)
