# Supplementary material for: Gene-Based Sequencing Identifies Lipid-Influencing Variants with Ethnicity-Specific Effects in African Americans
Source: PLoS Genet. 2014 Mar 6;10(3):e1004190. doi: 10.1371/journal.pgen.1004190 (PMC3945436; doi:10.1371/journal.pgen.1004190)
Supplement: Table S1 — Participant characteristics, Africa America Diabetes Mellitus Study. Characteristics of West African participants from the Africa America Diabetes Mellitus Study evaluated for replication. (DOCX) [file pgen.1004190.s001.docx]

|  | **West Africans** |
| --- | --- |
| N | 528 |
| Age | 47.7 (± 13.4) |
| % Women | 65.5% |
| BMI | 26.3 (± 6.4) |
| HDL | 39.3 (± 12.4) |
| TG^1^ | 75.5 (± 41.5) |

**Table S1.** Participant Characteristics, Africa America Diabetes Mellitus Study

*^1^Median and interquartile range presented due to non-normality of distribution.*
