# Supplementary material for: Gene-Based Sequencing Identifies Lipid-Influencing Variants with Ethnicity-Specific Effects in African Americans
Source: PLoS Genet. 2014 Mar 6;10(3):e1004190. doi: 10.1371/journal.pgen.1004190 (PMC3945436; doi:10.1371/journal.pgen.1004190)
Supplement: Table S2 — Variants found exclusively in one of the sequencing extremes. Variants that were exclusively found in either the Favorable or Unfavorable Lipid Group during the Sequencing Stage. (DOCX) [file pgen.1004190.s002.docx]

**Table S2.**  Variants found exclusively in one of the Sequencing Extremes

| **Extreme Group** | **Gene** | **SNP^1^** | **Chr** | **Position^2^** | **Ref/Alt Allele** | **HUFS MAF^3^** | **1000 Genomes Frequency (AFR/EUR)** | **Variant**  **Type** |
| --- | --- | --- | --- | --- | --- | --- | --- | --- |
| **Favorable^4^** | *ABCA1* | rs112644588 | 9 | 107526304 | A/G | 0.03 | 0.01/0 | 3' |
|  | *ABCA1* | rs116660941 | 9 | 107526427 | G/C | 0.03 | 0.06/0 | 3' |
|  | *ABCA1* | rs147942567 | 9 | 107526663 | G/T | 0.03 | 0.01/0 | 3' |
|  | *ABCA1* | rs78973029 | 9 | 107528266 | G/A | 0.03 | 0.06/0 | 3' |
|  | *ABCA1* | rs114587458 | 9 | 107529957 | T/G | 0.03 | 0.06/0 | 3' |
|  | *ABCA1* | rs139927095 | 9 | 107532481 | T/C | 0.03 | 0.01/0 | 3' |
|  | *ABCA1* | rs151080234 | 9 | 107532668 | G/A | 0.01 | 0.004/0 | 3' |
|  | *ABCA1* | rs112148734 | 9 | 107536939 | C/T | 0.03 | 0.02/0 | 3' |
|  | *ABCA1* | rs113533802 | 9 | 107537064 | A/T | 0.03 | 0.02/0 | 3' |
|  | *ABCA1* | rs114721211 | 9 | 107537851 | T/A | 0.03 | 0.06/0 | 3' |
|  | *ABCA1* | rs78492621 | 9 | 107538082 | A/G | 0.02 | 0.03/0 | 3' |
|  | *ABCA1* | rs2482430 | 9 | 107539352 | C/T | 0.1 | 0.05/0.21 | 3' |
|  | *ABCA1* | rs2482431 | 9 | 107540284 | T/C | 0.07 | 0.04/0.21 | 3' |
|  | *ABCA1* | rs150113640 | 9 | 107540778 | G/T | 0.01 | 0.004/0 | 3' |
|  | *ABCA1* | rs75141626 | 9 | 107543891 | C/T | 0.03 | 0.05/0 | 3' |
|  | *ABCA1* | rs363717 | 9 | 107544700 | T/C | 0.07 | 0.04/0.21 | 3' |
|  | *ABCA1* | rs34879708 | 9 | 107546653 | G/T | 0.01 | 0.02/0 | NonSyn |
|  | *ABCA1* | rs112590861 | 9 | 107547047 | C/T | 0.02 | 0.01/0 | Intronic |
|  | *ABCA1* | rs142026994 | 9 | 107547113 | C/T | 0.03 | 0.05/0 | Intronic |
|  | *ABCA1* | rs62566031 | 9 | 107548250 | T/A | 0.04 | 0.04/0.11 | Intronic |
|  | *ABCA1* | rs201153783 | 9 | 107548299 | A/AT | 0.02 | 0.03/0 | Intronic |
|  | *ABCA1* | rs116184208 | 9 | 107548886 | A/C | 0.02 | 0.03/0 | Intronic |
|  | *ABCA1* | rs114821402 | 9 | 107549065 | G/A | 0.03 | 0.05/0 | Intronic |
|  | *ABCA1* | rs62566032 | 9 | 107549827 | G/A | 0.03 | 0.02/0.12 | Intronic |
|  | *ABCA1* | rs116416613 | 9 | 107550632 | C/T | 0.02 | 0.03/0 | Intronic |
|  | *ABCA1* | rs116383884 | 9 | 107552097 | G/A | 0.02 | 0.02/0.003 | Intronic |
|  | *ABCA1* | rs146800165 | 9 | 107554439 | C/T | 0.02 | 0.02/0 | Intronic |
|  | *ABCA1* | rs116570904 | 9 | 107560130 | T/A | 0.03 | 0.05/0 | Intronic |
|  | *ABCA1* | rs116060323 | 9 | 107560396 | G/C | 0.03 | 0.03/0 | Intronic |
|  | *ABCA1* | rs113456348 | 9 | 107560513 | T/C | 0.02 | 0.01/0 | Intronic |
|  | *ABCA1* | rs191035288 | 9 | 107563336 | A/C | 0.002 | 0.01/0 | Intronic |
|  | *ABCA1* | rs115169883 | 9 | 107567964 | T/C | 0.03 | 0.04/0 | Intronic |
|  | *ABCA1* | rs79617159 | 9 | 107568936 | T/G | 0.03 | 0.03/0.01 | Intronic |
|  | *ABCA1* | rs114992777 | 9 | 107570479 | G/A | 0.03 | 0.03/0 | Intronic |
|  | *ABCA1* | rs73663551 | 9 | 107571824 | C/T | 0.003 | 0.004/0 | Syn |
|  | *ABCA1* | rs41335545 | 9 | 107572953 | A/G | 0.003 | 0.01/0 | Intronic |
|  | *ABCA1* | rs41410048 | 9 | 107576648 | A/G | 0.02 | 0.04/0 | Intronic |
|  | *ABCA1* | rs111337110 | 9 | 107576665 | A/G | 0.03 | 0.05/0.01 | Intronic |
|  | *ABCA1* | rs41436749 | 9 | 107576732 | T/G | 0.003 | 0.01/0 | NonSyn |
|  | *ABCA1* | rs58694166 | 9 | 107577200 | T/C | 0.03 | 0.03/0.01 | Intronic |
|  | *ABCA1* | rs33918808 | 9 | 107579632 | C/G | 0.17 | 0.16/0.03 | NonSyn |
|  | *ABCA1* | rs73663555 | 9 | 107580273 | G/A | 0.003 | 0.01/0 | Intronic |
|  | *ABCA1* | rs139397024 | 9 | 107583915 | C/T | 0.02 | 0.01/0 | Intronic |
|  | *ABCA1* | rs3818689 | 9 | 107585016 | G/C | 0.03 | 0.02/0.06 | Intronic |
|  | *ABCA1* | rs35093463 | 9 | 107586238 | C/A | 0.03 | 0.02/0.06 | Intronic |
|  | *ABCA1* | rs139457469 | 9 | 107586862 | G/A | 0.01 | 0.01/0 | Intronic |
|  | *ABCA1* | rs145248363 | 9 | 107587835 | A/C | 0.02 | 0.01/0 | Intronic |
|  | *ABCA1* | rs2066715 | 9 | 107588033 | C/T | 0.02 | 0.004/0.06 | NonSyn |
|  | *ABCA1* | rs148364457 | 9 | 107590658 | G/C | 0.02 | 0.01/0 | Intronic |
|  | *ABCA1* | rs13306069 | 9 | 107591024 | C/T | 0.03 | 0.02/0.06 | Intronic |
|  | *ABCA1* | rs2066717 | 9 | 107591478 | G/A | 0.03 | 0.02/0.06 | Intronic |
|  | *ABCA1* | rs191105105 | 9 | 107591573 | A/G | 0.01 | 0.01/0 | Intronic |
|  | *ABCA1* | rs116572835 | 9 | 107591779 | A/G | 0.02 | 0.01/0 | Intronic |
|  | *ABCA1* | rs139365336 | 9 | 107593624 | G/A | 0.02 | 0.01/0 | Intronic |
|  | *ABCA1* | rs34590907 | 9 | 107594088 | T/C | 0.01 | 0.01/0 | Syn |
|  | *ABCA1* | rs79983614 | 9 | 107596590 | C/T | 0.03 | 0.04/0 | Intronic |
|  | *ABCA1* | rs2297399 | 9 | 107599390 | G/A | 0.02 | 0.004/0.06 | Intronic |
|  | *ABCA1* | rs35545100 | 9 | 107600404 | C/G | 0.02 | 0.004/0.06 | Intronic |
|  | *ABCA1* | rs3824479 | 9 | 107604394 | T/A | 0.02 | 0.004/0.05 | Intronic |
|  | *ABCA1* | rs78733415 | 9 | 107604485 | C/A | 0.01 | 0.03/0 | Intronic |
|  | *ABCA1* | rs73521808 | 9 | 107605521 | C/G,T | 0.05 | 0.1/0.003 | Intronic |
|  | *ABCA1* | rs148714575 | 9 | 107606383 | C/T | 0.07 | 0.11/0 | Intronic |
|  | *ABCA1* | rs115761095 | 9 | 107606447 | G/C | -- | 0.11/0 | Intronic |
|  | *ABCA1* | rs76729624 | 9 | 107606514 | G/A | -- | 0.11/0 | Intronic |
|  | *ABCA1* | rs147489607 | 9 | 107606582 | C/A | 0.02 | 0.03/0 | Intronic |
|  | *ABCA1* | rs139941675 | 9 | 107606583 | C/G | 0.03 | 0.03/0 | Intronic |
|  | *ABCA1* | rs139257838 | 9 | 107609089 | CAGAT/C | 0.02 | 0.07/0 | Intronic |
|  | *ABCA1* | rs114089923 | 9 | 107611242 | A/C | 0.01 | 0.03/0 | Intronic |
|  | *ABCA1* | rs60326642 | 9 | 107612889 | T/A | 0.02 | 0.07/0 | Intronic |
|  | *ABCA1* | rs73521824 | 9 | 107613602 | A/G | 0.02 | 0.07/0 | Intronic |
|  | *ABCA1* | rs144501024 | 9 | 107617519 | G/A | 0.01 | 0.03/0 | Intronic |
|  | *ABCA1* | rs57782550 | 9 | 107617728 | C/T | 0.01 | 0.03/0 | Intronic |
|  | *ABCA1* | rs144449874 | 9 | 107623124 | G/A | 0.02 | 0.02/0.001 | Intronic |
|  | *ABCA1* | rs73504110 | 9 | 107623807 | G/C | 0.09 | 0.16/0 | Intronic |
|  | *ABCA1* | rs145623477 | 9 | 107624407 | C/T | 0.01 | 0.02/0 | Intronic |
|  | *ABCA1* | rs148160159 | 9 | 107625534 | G/A | 0.01 | 0.02/0 | Intronic |
|  | *ABCA1* | rs141765508 | 9 | 107636736 | T/C | 0.03 | 0.07/0.01 | Intronic |
|  | *ABCA1* | rs191098995 | 9 | 107637069 | C/T | 0.04 | 0.08/0.03 | Intronic |
|  | *ABCA1* | rs140277506 | 9 | 107638698 | A/G | 0.01 | 0.01/0.02 | Intronic |
|  | *ABCA1* | rs143972088 | 9 | 107646014 | G/A | 0.01 | 0.01/0 | Intronic |
|  | *ABCA1* | rs139609748 | 9 | 107655219 | A/G | 0.003 | 0.01/0 | Intronic |
|  | *ABCA1* | rs138960174 | 9 | 107657647 | T/C | 0.01 | 0.02/0 | Intronic |
|  | *ABCA1* | rs72735008 | 9 | 107658723 | C/T | 0.01 | 0.01/0.02 | Intronic |
|  | *ABCA1* | rs73504185 | 9 | 107660938 | T/C | 0.05 | 0.05/0 | Intronic |
|  | *ABCA1* | rs77780610 | 9 | 107662659 | A/C | 0.01 | 0.01/0.02 | Intronic |
|  | *ABCA1* | rs73506109 | 9 | 107664475 | A/T | 0.04 | 0.03/0.001 | Intronic |
|  | *ABCA1* | rs113310792 | 9 | 107670866 | T/C | 0.04 | 0.07/0.003 | Intronic |
|  | *ABCA1* | rs150414107 | 9 | 107677049 | T/G | 0.01 | 0.02/0 | Intronic |
|  | *ABCA1* | rs149413390 | 9 | 107677938 | A/G | 0.02 | 0.02/0 | Intronic |
|  | *ABCA1* | rs10512335 | 9 | 107680639 | T/G | 0.11 | 0.14/0.06 | Intronic |
|  | *ABCA1* | rs73506147 | 9 | 107681194 | G/C | 0.11 | 0.14/0.06 | Intronic |
|  | *ABCA1* | rs7872096 | 9 | 107681614 | C/T | 0.1 | 0.14/0.04 | Intronic |
|  | *ABCA1* | rs7035444 | 9 | 107683257 | A/T | 0.08 | 0.12/0.001 | Intronic |
|  | *ABCA1* | rs147859690 | 9 | 107683299 | G/A | 0.01 | 0.02/0 | Intronic |
|  | *ABCA1* | rs12342233 | 9 | 107683594 | C/A | 0.1 | 0.16/0.04 | Intronic |
|  | *ABCA1* | rs144441671 | 9 | 107685767 | T/C | 0.01 | 0.02/0 | Intronic |
|  | *ABCA1* | rs115919518 | 9 | 107687896 | C/G | 0.03 | 0.04/0 | Intronic |
|  | *ABCA1* | rs139686313 | 9 | 107689802 | T/C | 0.01 | 0.02/0 | Intronic |
|  | *LPL* | rs13266204 | 8 | 19800005 | A/G | 0.06 | 0.05/0.24 | Intronic |
|  | *LPL* | rs11542065 | 8 | 19805815 | C/G | 0.01 | 0.02/0 | NonSyn |
|  | *LPL* | rs306 | 8 | 19817443 | G/A | 0.05 | 0.07/0 | Intronic |
|  | *LPL* | rs149089920 | 8 | 19819645 | G/A | 0.002 | -- | NonSyn |
|  | *LPL* | rs150647190 | 8 | 19820853 | G/A | 0.06 | 0.07/0 | Intronic |
|  | *LPL* | 8_19867705 | 8 | 19823425 | C/T | -- | -- | 3' |
|  | *LPL* | 8_19868510 | 8 | 19824230 | T/G | 0.001 | -- | 3' |
|  | *LPL* | rs188554527 | 8 | 19824628 | G/T | 0.02 | 0/0 | 3' |
|  | *PON1* | rs3917577 | 7 | 94927708 | T/C | 0.1 | 0.09/0.11 | 3' |
|  | *PON1* | rs80019660 | 7 | 94937419 | G/A | -- | 0/0 | NonSyn |
|  | *PON1* | rs854560 | 7 | 94946084 | A/T | 0.17 | 0.15/0.35 | NonSyn |
|  | *SERPINE1* | rs2227696 | 7 | 100780890 | C/T | 0.01 | 0/0 | 3' |
| **Unfavorable^5^** | *ABCA1* | rs141665560 | 9 | 107528655 | A/T | 0.02 | 0.04/0 | 3'^6^ |
|  | *ABCA1* | rs138217135 | 9 | 107542636 | G/C | 0.03 | 0.02/0 | 3' |
|  | *ABCA1* | rs148080589 | 9 | 107543345 | A/G | 0.01 | 0.01/0 | 3' |
|  | *ABCA1* | 9_106583788 | 9 | 107543967 | C/A | 0.0005 | 0/0 | 3' |
|  | *ABCA1* | rs74316246 | 9 | 107546500 | A/G | 0.01 | 0.03/0 | 3' |
|  | *ABCA1* | rs141566813 | 9 | 107551651 | T/G | 0.004 | 0.01/0 | Intronic |
|  | *ABCA1* | rs140520600 | 9 | 107554440 | G/A | 0.01 | 0.01/0 | Intronic |
|  | *ABCA1* | 9_106594891 | 9 | 107555070 | C/T | -- | -- | Syn |
|  | *ABCA1* | rs138341589 | 9 | 107555847 | C/A | 0.003 | 0.004/0 | Intronic |
|  | *ABCA1* | rs112161989 | 9 | 107562939 | T/A | 0.03 | 0.03/0 | Intronic |
|  | *ABCA1* | rs111840495 | 9 | 107565515 | C/T | 0.03 | 0.03/0 | Intronic |
|  | *ABCA1* | rs181469381 | 9 | 107567472 | C/T | 0.02 | 0.01/0 | Intronic |
|  | *ABCA1* | rs112335062 | 9 | 107569386 | G/A | 0.02 | 0.03/0 | Intronic |
|  | *ABCA1* | rs116034780 | 9 | 107571771 | C/T | 0.005 | 0.01/0 | NonSyn |
|  | *ABCA1* | rs112338016 | 9 | 107578676 | G/A | 0.02 | 0.02/0 | Intronic |
|  | *ABCA1* | rs2020926 | 9 | 107580842 | T/C | 0.02 | 0.01/0.05 | Intronic |
|  | *ABCA1* | rs78329992 | 9 | 107582360 | G/A | 0.01 | 0.03/0 | Intronic |
|  | *ABCA1* | rs35561837 | 9 | 107583748 | G/A | 0.02 | 0.05/0 | Syn |
|  | *ABCA1* | rs9282546 | 9 | 107584791 | C/T | 0.01 | 0.02/0 | Syn |
|  | *ABCA1* | rs184186942 | 9 | 107585602 | C/T | 0.01 | 0.02/0 | Intronic |
|  | *ABCA1* | rs35207495 | 9 | 107586800 | C/T | 0.01 | 0.01/0 | NonSyn |
|  | *ABCA1* | rs77755970 | 9 | 107587780 | T/A | 0.02 | 0.05/0 | Intronic |
|  | *ABCA1* | rs74821460 | 9 | 107588613 | T/G | 0.02 | 0.05/0 | Intronic |
|  | *ABCA1* | rs35819696 | 9 | 107589246 | T/G | -- | 0/0.01 | NonSyn |
|  | *ABCA1* | rs114427540 | 9 | 107591623 | T/C | 0.03 | 0.03/0 | Intronic |
|  | *ABCA1* | rs41504247 | 9 | 107593411 | T/C | 0.01 | 0.02/0 | Intronic |
|  | *ABCA1* | rs55874167 | 9 | 107602342 | T/G | 0.03 | 0.08/0.09 | Intronic |
|  | *ABCA1* | rs114893478 | 9 | 107604842 | C/T | 0.02 | 0.01/0 | Intronic |
|  | *ABCA1* | 9_106645883 | 9 | 107606062 | G/A | -- | -- | Intronic |
|  | *ABCA1* | rs116728780 | 9 | 107607741 | G/A | 0.03 | 0.08/0 | Intronic |
|  | *ABCA1* | rs77257670 | 9 | 107608319 | T/C | 0.03 | 0.08/0 | Intronic |
|  | *ABCA1* | rs114512051 | 9 | 107608654 | G/A | 0.03 | 0.08/0 | Intronic |
|  | *ABCA1* | rs79227686 | 9 | 107608845 | T/C | 0.03 | 0.08/0 | Intronic |
|  | *ABCA1* | rs114548000 | 9 | 107610890 | G/A | 0.03 | 0.08/0 | Intronic |
|  | *ABCA1* | rs144360419 | 9 | 107611279 | C/T | 0.03 | 0.08/0.001 | Intronic |
|  | *ABCA1* | rs146415421 | 9 | 107612459 | G/C | 0.01 | 0.05/0.001 | Intronic |
|  | *ABCA1* | rs200523782 | 9 | 107615653 | CCA/C | 0.02 | 0.06/0 | Intronic |
|  | *ABCA1* | rs1340352 | 9 | 107615839 | C/T | 0.01 | 0.02/0 | Intronic |
|  | *ABCA1* | rs149407634 | 9 | 107616334 | T/C | 0.01 | 0.04/0 | Intronic |
|  | *ABCA1* | rs75671121 | 9 | 107616778 | G/A | 0.02 | 0.03/0 | Intronic |
|  | *ABCA1* | rs144999512 | 9 | 107618456 | A/G | 0.01 | 0.01/0 | Intronic |
|  | *ABCA1* | rs144431307 | 9 | 107619670 | C/T | 0.03 | 0.07/0.01 | Intronic |
|  | *ABCA1* | rs9282538 | 9 | 107620872 | T/C | 0.01 | 0.01/0 | Syn |
|  | *ABCA1* | rs115216814 | 9 | 107620889 | A/T | 0.01 | 0.02/0 | NonSyn |
|  | *ABCA1* | rs116171343 | 9 | 107622883 | A/C | 0.01 | 0.04/0 | Intronic |
|  | *ABCA1* | rs139044915 | 9 | 107623024 | T/G | 0.01 | 0.01/0 | Intronic |
|  | *ABCA1* | rs114415472 | 9 | 107627648 | T/C | 0.03 | 0.03/0 | Intronic |
|  | *ABCA1* | rs116144254 | 9 | 107627974 | A/C | 0.03 | 0.08/0 | Intronic |
|  | *ABCA1* | rs114125287 | 9 | 107627987 | A/C | 0.03 | 0.08/0 | Intronic |
|  | *ABCA1* | rs140563773 | 9 | 107629034 | G/T | 0.03 | 0.07/0 | Intronic |
|  | *ABCA1* | rs61602310 | 9 | 107632615 | A/G | 0.02 | 0.03/0 | Intronic |
|  | *ABCA1* | rs114163629 | 9 | 107632661 | G/A | 0.09 | 0.16/0 | Intronic |
|  | *ABCA1* | rs115327636 | 9 | 107636807 | G/A | 0.03 | 0.05/0.001 | Intronic |
|  | *ABCA1* | rs150646225 | 9 | 107637492 | C/T | 0.01 | 0.02/0 | Intronic |
|  | *ABCA1* | rs140779255 | 9 | 107637608 | G/A | 0.02 | 0.06/0 | Intronic |
|  | *ABCA1* | rs75906492 | 9 | 107640625 | T/G | 0.07 | 0.07/0 | Intronic |
|  | *ABCA1* | rs140087151 | 9 | 107641205 | G/A | 0.01 | 0.04/0 | Intronic |
|  | *ABCA1* | rs78853242 | 9 | 107647973 | C/T | 0.07 | 0.07/0 | Intronic |
|  | *ABCA1* | rs7026408 | 9 | 107649758 | G/A | 0.02 | 0.04/0 | Intronic |
|  | *ABCA1* | rs116564772 | 9 | 107650282 | G/A | 0.02 | 0.04/0 | Intronic |
|  | *ABCA1* | rs142156695 | 9 | 107650533 | G/A | 0.003 | 0.01/0.02 | Intronic |
|  | *ABCA1* | rs138492267 | 9 | 107651030 | G/T | 0.003 | 0.01/0.02 | Intronic |
|  | *ABCA1* | rs115720069 | 9 | 107651164 | T/C | 0.02 | 0.04/0 | Intronic |
|  | *ABCA1* | rs145842792 | 9 | 107654006 | C/T | 0.03 | 0.06/0.003 | Intronic |
|  | *ABCA1* | rs150713429 | 9 | 107655199 | T/C | 0.003 | 0.002/0.02 | Intronic |
|  | *ABCA1* | rs76304226 | 9 | 107655953 | T/C | 0.02 | 0.02/0 | Intronic |
|  | *ABCA1* | rs79271694 | 9 | 107657986 | A/C | 0.09 | 0.11/0 | Intronic |
|  | *ABCA1* | rs75494727 | 9 | 107658339 | T/C | 0.07 | 0.07/0 | Intronic |
|  | *ABCA1* | rs56000254 | 9 | 107659978 | G/A | 0.01 | 0.02/0 | Intronic |
|  | *ABCA1* | rs201966762 | 9 | 107660431 | GATT/G | 0.03 | 0.04/0 | Intronic |
|  | *ABCA1* | rs79794931 | 9 | 107663644 | C/T | 0.03 | 0.04/0 | Intronic |
|  | *ABCA1* | rs200057994 | 9 | 107668246 | AG/A | 0.02 | 0.04/0 | Intronic |
|  | *ABCA1* | rs76316220 | 9 | 107669208 | C/G | 0.02 | 0.04/0 | Intronic |
|  | *ABCA1* | rs56848416 | 9 | 107669209 | G/A | 0.01 | 0.02/0 | Intronic |
|  | *ABCA1* | rs143427562 | 9 | 107670317 | C/T | 0.02 | 0.04/0 | Intronic |
|  | *ABCA1* | rs115896485 | 9 | 107677353 | C/T | 0.02 | 0.03/0 | Intronic |
|  | *ABCA1* | rs75684347 | 9 | 107678835 | G/A | 0.01 | 0.01/0 | Intronic |
|  | *ABCA1* | rs12343571 | 9 | 107684626 | C/A | 0.03 | 0.06/0.04 | Intronic |
|  | *ABCA1* | rs116671436 | 9 | 107684675 | T/G | 0.003 | 0.01/0 | Intronic |
|  | *ABCA1* | rs200931562 | 9 | 107687122 | TA/T | 0.01 | 0.02/0.02 | Intronic |
|  | *ABCA1* | rs79739357 | 9 | 107689865 | G/A | 0.03 | 0.04/0 | Intronic |
|  | *ABCA1* | rs79440945 | 9 | 107690039 | G/A | 0.03 | 0.04/0 | Intronic |
|  | *LPL* | 8_19841125 | 8 | 19796845 | G/A | 0.002 | -- | 5' |
|  | *LPL* | 8_19856055 | 8 | 19811775 | A/G | -- | -- | NonSyn |
|  | *LPL* | rs256 | 8 | 19811967 | C/T | 0.06 | 0.05/0.13 | Intronic |
|  | *LPL* | rs268 | 8 | 19813529 | A/G | -- | 0/0.02 | NonSyn |
|  | *LPL* | 8_19857854 | 8 | 19813574 | G/A | -- | -- | NonSyn |
|  | *LPL* | rs271 | 8 | 19813702 | G/A | 0.06 | 0.04/0.14 | Intronic |
|  | *LPL* | rs310 | 8 | 19817546 | C/T | 0.07 | 0.06/0.12 | Intronic |
|  | *LPL* | rs5934 | 8 | 19818551 | G/A | 0.02 | 0.03/0 | NonSyn |
|  | *LPL* | rs325 | 8 | 19819328 | T/C | 0.07 | 0.05/0.12 | Intronic |
|  | *LPL* | rs328 | 8 | 19819724 | C/G | 0.07 | 0.05/0.12 | NonSyn |
|  | *LPL* | rs12679834 | 8 | 19820433 | T/C | 0.08 | 0.09/0.12 | Intronic |
|  | *LPL* | rs117199990 | 8 | 19820916 | C/T | 0.07 | 0.05/0.12 | Intronic |
|  | *LPL* | rs145391587 | 8 | 19820933 | A/C | 0.07 | 0.05/0.12 | Intronic |
|  | *LPL* | rs75278536 | 8 | 19821425 | T/G | 0.07 | 0.05/0.13 | Intronic |
|  | *LPL* | rs77069344 | 8 | 19821782 | T/G | 0.07 | 0.05/0.13 | Intronic |
|  | *LPL* | rs11570891 | 8 | 19822810 | C/T | 0.07 | 0.05/0.13 | Intronic |
|  | *LPL* | rs4922115 | 8 | 19822830 | G/A | 0.12 | 0.1/0.14 | 3' |
|  | *LPL* | rs1803924 | 8 | 19823674 | C/G,T | 0.06 | 0.05/0.13 | 3' |
|  | *LPL* | rs150252331 | 8 | 19823988 | C/A | 0.001 | 0.002/0.004 | 3' |
|  | *LPL* | rs3735964 | 8 | 19824045 | C/A | 0.06 | 0.05/0.13 | 3' |
|  | *LPL* | rs3866471 | 8 | 19824669 | C/A | 0.22 | 0.24/0.14 | 3' |
|  | *LPL* | rs147116359 | 8 | 19824707 | G/A | -- | 0.02/0 | 3' |
|  | *PON1* | rs854550 | 7 | 94927242 | C/T | -- | 0.78/0.81 | 3' |
|  | *PON1* | rs367585159 | 7 | 94927864 | C/T | 0.007 | -- | 3' |
|  | *PON1* | rs3917552 | 7 | 94934259 | T/G | 0.06 | 0.11/0 | Intronic |
|  | *PON1* | rs2299258 | 7 | 94942917 | G/A | 0.06 | 0.04/0.12 | Intronic |
|  | *PON1* | 7_94791772 | 7 | 94953836 | G/C | -- | -- | 5' |
|  | *SERPINE1* | rs6092 | 7 | 100771717 | G/A | 0.02 | 0/0.09 | NonSyn |
|  | *SERPINE1* | 7_100558485 | 7 | 100771765 | G/A | -- | -- | NonSyn |
|  | *SERPINE1* | rs41334349 | 7 | 100780903 | C/T | 0.001 | 0.01/0.01 | 3' |
|  | *SERPINE1* | rs2227697 | 7 | 100781028 | G/A | -- | -- | 3' |
|  | *SERPINE1* | rs7241 | 7 | 100781413 | T/C | -- | 0/0.05 | 3' |
|  | *SERPINE1* | rs2227698 | 7 | 100781468 | C/A | 0.05 | 0.03/0 | 3' |

*Abbreviations: Chromosome (Chr), Minor Allele Frequency (MAF), High-density Lipoprotein Cholesterol (HDL-C), Howard University Family Study (HUFS), Log-transformed Triglycerides (logTG), Non-Synonymous Variant (NonSyn), Synonymous Variant (Syn); ^1^“Chromosome_Build 37 Position” used in the absence of rsid for this variant; ^2^Build 37 position; ^3^Minor Allele Frequency for those variants that were successfully genotyped or imputed in the full Howard University Family Study population; ^4^Individuals in this group were selected based on extremely high HDLC and low TG;^5^Individuals in this group were selected based on extremely low HDLC and high TG; ^6^rs141665560 is a missense variant in a gene upstream of ABCA1, NIPSNAP3B.*
