# Supplementary material for: Gene-Based Sequencing Identifies Lipid-Influencing Variants with Ethnicity-Specific Effects in African Americans
Source: PLoS Genet. 2014 Mar 6;10(3):e1004190. doi: 10.1371/journal.pgen.1004190 (PMC3945436; doi:10.1371/journal.pgen.1004190)
Supplement: Table S3 — Local ancestry interactions among serum lipids-associated LPL variants. Associations between Serum Lipids-associated LPL variants within Local Ancestry Strata. (DOCX) [file pgen.1004190.s003.docx]

**Table S3: Local Ancestry Interactions among Serum Lipids-associated *LPL* Variants**

| **SNP** | **Chr 8 Position^1^** | **Trait** | **Number of African Ancestry Alleles^2^** | | | | **P_interaction_^3^** |
| --- | --- | --- | --- | --- | --- | --- | --- |
|  |  |  | **0** | **1** | | **2** |  |
| rs256 | 19811967 | HDLC | 5.0 (-3.9, 13.9) | | -0.07 (-3.8, 3.7) | -0.1 (-3.2, 3.0) | 0.13 |
| rs271 | 19813702 | HDLC | 3.4 (-5..4, 12.2) | | -0.01 (-3.5, 3.5) | -0.2 (-3.2, 2.8) | 0.25 |
| rs325^4^ | 19819328 | HDLC | 11.5 (1.7, 21.2) | 1.5 (-1.9, 4.9) | | 0.3 (-2.3, 2.9) | 0.01 |
|  |  |  | -10.4% (-25.4, 4.6) | -10.4% (-15.3, -5.5) | | -2.6% (-6.1, 1.0) | 0.01 |
| rs328 | 19819724 | HDLC | 12.7 (3.0, 22.4) | 1.9 (-1.6, 5.3) | | 1.1 (-1.5, 3.6) | 0.008 |
|  |  | logTG | -10.5% (-25.3, 4.4) | -10.6% (-15.5, -5.6) | | -3.3% (-6.8, 0.1) | 0.01 |
| rs12679834^4^ | 19820433 | logTG | -10.4% (-25.4, 4.6) | -9.9% (-14.6, -5.3) | | -2.9% (-6.0, 0.3) | 0.01 |
| rs117199990^4^ | 19820916 | HDLC | 11.5 (1.7, 21.2) | 1.2 (-2.2, 4.7) | | 0.3 (-2.3, 2.9) | 0.009 |
|  |  | logTG | -10.4% (-25.4, 4.6) | -10.3% (-15.2, -5.4) | | -2.6% (-6.1, 1.0) | 0.01 |
| rs145391587^4^ | 19820933 | HDLC | 11.5 (1.7, 21.2) | 1.2 (-2.2, 4.7) | | 0.3 (-2.3, 2.9) | 0.009 |
|  |  | logTG | -10.4% (-25.4, 4.6) | -10.3% (-15.2, -5.4) | | -2.6% (-6.1, 1.0) | 0.01 |
| rs75278536^4^ | 19821425 | HDLC | 11.5 (1.7, 21.2) | 1.3 (-2.1, 4.6) | | 0.2 (-2.4, 2.9) | 0.009 |
|  |  | logTG | -10.4% (-25.4, 4.6) | -9.4% (-14.3, -4.5) | | -3.0% (-6.6, 0.6) | 0.03 |
| rs201109344 | 19821465 | HDLC | 0.4 (-2.9, 3.7) ^5^ | | | 3.7 (1.6, 5.8) | 0.12 |
| rs77069344^4^ | 19821782 | HDLC | 11.5 (1.7, 21.2) | 1.3 (-2.1, 4.6) | | 0.2 (-2.4, 2.9) | 0.009 |
|  |  | logTG | -10.4% (-25.4, 4.6) | -9.4% (-14.3, -4.5) | | -3.0% (-6.6, 0.6) | 0.03 |
| rs11570891^4^ | 19822810 | HDLC | 11.5 (1.7, 21.2) | 1.5 (-2.0, 4.9) | | 0.2 (-2.5, 2.8) | 0.009 |
|  |  | logTG | -10.4% (-25.4, 4.6) | -10.0% (-15.1, 5.0) | | -2.1% (-5.9, 1.7) | 0.01 |
| rs1803924^4^ | 19823674 | HDLC | 11.5 (1.7, 21.2) | 1.5 (-2.0, 5.0) | | 0.03 (-2.8, 2.8) | 0.009 |
|  |  | logTG | -10.4% (-25.4, 4.6) | -9.1% (-14.1, -4.1) | | -2.1% (-5.9, 1.7) | 0.02 |
| rs3735964^4^ | 19824045 | HDLC | 9.2 (-2.0, 20.3) | 2.3 (-1.4, 5.9) | | 0.7 (-2.2, 3.6) | 0.08 |
|  |  | logTG | -9.3% (-26.0, 7.4) | -10.6% (-15.9, -5.2) | | -2.6% (-6.5, 1.4) | 0.01 |
| rs1059611 | 19824563 | HDLC | 14.6 (5.3, 23.8) | 0.9 (-1.6, 3.4) | | 1.8 (-0.02, 3.5) | 0.004 |
| rs149865365 | 19824626 | HDLC | 14.6 (5.3, 23.8) | 0.8 (-1.8, 3.3) | | 1.8 (0.003, 3.5) | 0.004 |

*^1^ Build 37 Position; ^2^ Given is the ß (95% CI) for the SNP-Trait within the listed strata of local ancestry. ^3^P value for an interaction term between the SNP and local African ancestry. ^4^ R^2^>0.6 with rs328; ^5^ Given the fewness of individuals within ancestry strata (n<5), individuals with either 0 or 1 local African Ancestry allele were considered together. Note: for each individual in this analysis, local African ancestry was the same across all of these SNPs (i.e. there were no ancestry “switches” in this region).*
