# Supplementary material for: Gene-Based Sequencing Identifies Lipid-Influencing Variants with Ethnicity-Specific Effects in African Americans
Source: PLoS Genet. 2014 Mar 6;10(3):e1004190. doi: 10.1371/journal.pgen.1004190 (PMC3945436; doi:10.1371/journal.pgen.1004190)
Supplement: Table S4 — Primers used. Further details on the set of primers used for novel genotyping of variants discovered in the sequencing stage. (DOCX) [file pgen.1004190.s004.docx]

**Table S4.** Primers Used

| **Marker** | **5' Capture Primer sequence** | **3' Capture Primer sequence** | **Extend Mass (Da)** | **Extend Primer Sequence** |
| --- | --- | --- | --- | --- |
| 7.94927864 | ACGTTGGATGATGTGTGTATCGCTGCCTTG | ACGTTGGATGTCTGGCCTGTGAACTGGAG | 5236.4 | AAAGACACGTGAGCTAA |
| 9.107543967 | ACGTTGGATGTCTTTGGGACACCTCAGAAA | ACGTTGGATGCAGATAATGAAATACAGTAGTGTGAGG | 6516.2 | CTGGGCATTTATGTATAGAGG |
| rs1059611 | ACGTTGGATGTTGGCTCTGTGAGACCATCA | ACGTTGGATGTCTGGCTCCGAAAAACTTTG | 7825.1 | ctcaCTTTCCAATATGTACAAGCTCC |
| rs114719973 | ACGTTGGATGGCAAAGCTATCTCCTCTGAATGA | ACGTTGGATGGGGAACACATGCCCTTTTAT | 7104.7 | GAATGACATCTTAGAAATCAAGG |
| rs115216814 | ACGTTGGATGTGTGCAATGGATCAAAATCAG | ACGTTGGATGCAGGATTGGCTTCAGGATGT | 7457.9 | ggggcACTTGGTGACCAAGAAGTT |
| rs11542065 | ACGTTGGATGAGCCTCCCTTACCGTCCA | ACGTTGGATGGAGGACACTTGCCACCTCAT | 5865.8 | ggcTGAAGGTTTTGCTGCT |
| rs116034780 | ACGTTGGATGTGCCTCTCATGTACCCACAG | ACGTTGGATGGCCTGCTTATGGCTAAAGTGG | 5465.6 | GGGTTTCCTTCCATACAG |
| rs13702 | ACGTTGGATGCAGATAGCCACAATGACCTTTC | ACGTTGGATGTCATCAGGCTTGGAATGAAT | 6092 | TCTGGCTCCGAAAAACTTTG |
| rs139240067 | ACGTTGGATGGGACAACACACATGCAGAGC | ACGTTGGATGCAACTAATCAAGAGTGAGTGAACAAC | 6988.6 | cGTGTTCAGATATCACATACTTT |
| rs139457469 | ACGTTGGATGTCAAGGAGGAAATGGACCTG | ACGTTGGATGGGGTGGCTCTTCTCATCACT | 5474.6 | gaAATTCCGTACTGGCCT |
| rs139941675 | ACGTTGGATGCAGCTTGCCATTTGTTACCC | ACGTTGGATGAGCTACGGACTTGCAAAACA | 6715.4 | AGGCTGGTAACCTTTTTTACTA |
| rs145405273 | ACGTTGGATGAGGGCTTTGCTCTCCATCTC | ACGTTGGATGTAAGCAGCCTTGGCGTGAA | 5266.4 | gTTGGCGCTGAGCAAGT |
| rs147489607 | ACGTTGGATGCAGCTTGCCATTTGTTACCC | ACGTTGGATGAGCTACGGACTTGCAAAACA | 7662 | tAAGGCTGGTAACCTTTTTTACTAG |
| rs148911901 | ACGTTGGATGACCAAGGACAAGAGGCAACT | ACGTTGGATGAAACAGTAGCTGGGAATAAAGTCA | 8009.2 | gCTCTGAGTCATAGAAGAAGATTTTC |
| rs150252331 | ACGTTGGATGCAGTCGGGTTCCCAGCTAT | ACGTTGGATGTAAAAGGGTGGAGAGGTTCC | 7816.1 | ccccAAATAGTTGTTCACTCACTCTT |
| rs17091815 | ACGTTGGATGTTGGCTCTGTGAGACCATCA | ACGTTGGATGGCTGTAAATACATGTGTGGATGTG | 6339.1 | ccctTCATTGTGGCTATCTGC |
| rs1800590 | ACGTTGGATGGCCAAGGCTGCTTATGTGA | ACGTTGGATGATTTTTCCGTCTGCCCTTTC | 6837.5 | AACGTTTAGAAGTGAATTTAGG |
| rs187374932 | ACGTTGGATGCAAAATATGCTGAGTGAATCTGAC | ACGTTGGATGTGGAAAGGTCATTGTGGCTA | 6666.3 | ccttTTGTTTTGTTAAAGCCCA |
| rs188554527 | ACGTTGGATGAAAATATGCTGAGTGAATCTGACC | ACGTTGGATGCATATTGGAAAGGTCATTGTGG | 7852.1 | cctcCCATCACTGATAAAGACACATA |
| rs2066714 | ACGTTGGATGGCTTTGCTTTCAGGCCAGT | ACGTTGGATGCAGAGGCAGCAGCACTAGGT | 6924.5 | tttccACAGCACTTACTTTCTGA |
| rs2066715 | ACGTTGGATGTGCCCTTTTTGAGGAGCAG | ACGTTGGATGGATGCCCAAAGCAGTGTACC | 8709.7 | cccccGATGGCTTCAATCTCACCACTTCG |
| rs2066718 | ACGTTGGATGCATCTACTTCACGCTGTACCTG | ACGTTGGATGTCCAAAGGAAGGTCAAATGC | 4602 | GCATGGCAGGACTAC |
| rs2227696 | ACGTTGGATGTCCTGCAAAGGGCCTCAG | ACGTTGGATGTTCTGAAGGAAGAGAAGACATTTG | 6027.9 | cttaTCCAAGACCTTGGCCT |
| rs2227698 | ACGTTGGATGAGCACTCAAGGGCAAGGAT | ACGTTGGATGGTGCATGGGTTATTTTGGAGT | 4519.9 | GCGTGCCCAGCTCTT |
| rs2227714 | ACGTTGGATGGTCCACTGCTCACACACAGC | ACGTTGGATGGGGATCAAAAGGACGGAGTG | 6952.5 | ccGGACAGAACTTTCCCCAATTA |
| rs2230808 | ACGTTGGATGTGTTGCCCCTTATCTATGTGA | ACGTTGGATGCCCTGCCAACTTTACCATGA | 4571 | CAGGACTGGACACCA |
| rs268 | ACGTTGGATGCAGTCTCCAGCCTACCTTTGT | ACGTTGGATGCCATTCATCTCTTCATCGACTCT | 7275.7 | ccgaTTCTTTTGGCTCTGACTTTA |
| rs3200218 | ACGTTGGATGGGACAACACACATGCAGAGC | ACGTTGGATGCAACTAATCAAGAGTGAGTGAACAAC | 5802.8 | GCCTCTGAGATATTTGACA |
| rs328 | ACGTTGGATGTTAGGGTGCAAGCTCAGGAT | ACGTTGGATGGATCTTCTGTTCTAGGGAGAAAGTG | 4496.9 | AATGCTCACCAGCCT |
| rs3289 | ACGTTGGATGTCCAATAGCAAGAGGGAATCA | ACGTTGGATGTTTGAGCGCAGAGTAAAATAAGG | 7637 | gtTTGGTTAGAACCTCCTATTTTAA |
| rs33918808 | ACGTTGGATGACCTCCTTCTTTGCCATCAG | ACGTTGGATGCAGGCACCACCTGAATAAGA | 4931.2 | TGGGCAGCGACCATGA |
| rs34879708 | ACGTTGGATGCCAAGGACCAAAGTGATGATG | ACGTTGGATGCCCGTATGAACAGGATTCTTC | 6183.1 | AAAGATGTGAGAACTGCAAC |
| rs3735964 | ACGTTGGATGCAGTCGGGTTCCCAGCTAT | ACGTTGGATGCAACTAATCAAGAGTGAGTGAACAAC | 6626.3 | cCCTATTTTTCAGAATGCTCTT |
| rs3917577 | ACGTTGGATGCCAGTTCACAGGCCAGAAA | ACGTTGGATGGCAAGAGTACAAGGGGAGTTG | 5953.9 | cCCCATTTTCCCTTCGATAT |
| rs41436749 | ACGTTGGATGGACTACTCTCGTAATGTTGGTGCT | ACGTTGGATGGTGTGCCATTCTCCCTCAAG | 4426.9 | CAGCATCCACCCCAC |
| rs4149338 | ACGTTGGATGCCCTCTGATAAGCTGTTCTGGT | ACGTTGGATGTGGCTCGAAGCATAGGACA | 5386.5 | cCCAAGTTTCCCGTGCCT |
| rs4922115 | ACGTTGGATGTGAAACACCCCAAACACTGG | ACGTTGGATGGCGGGAATTGTAAAACACTCA | 5523.6 | agTTCTCAGAAACTGGGC |
| rs58998793 | ACGTTGGATGAATTCATTCCAAGCCTGATG | ACGTTGGATGAAAACAGTCGATCAAGGGATGT | 6487.2 | ggggCGAAGTTCTGGCACATC |
| rs5934 | ACGTTGGATGCATCAGGTGGGGGTCTAAAG | ACGTTGGATGTGAAGCTCAAATGGAAGAGTGA | 6706.4 | TTTACTCTGATCTTCTGAATGG |
| rs6090 | ACGTTGGATGACCTGCTGAAACACCCTCAC | ACGTTGGATGAGCCCTCTTTCCATTGCTCT | 4819.2 | GACCCTTCACCAAAGA |
| rs6092 | ACGTTGGATGACCTGCTGAAACACCCTCAC | ACGTTGGATGTTGCTCTAGGATGCAGATGTC | 4560 | CTAGTCCTGGGCCTG |
| rs662 | ACGTTGGATGCTGTGGGACCTGAGCACTTT | ACGTTGGATGCTTCTGCCACCACTCGAACT | 6298.1 | gTTTTCTTGACCCCTACTTAC |
| rs73504110 | ACGTTGGATGCCTTGTCCTTGTAATGGTGGT | ACGTTGGATGATGACGCCAGGTTGCATATT | 8374.5 | cacCTTCACTTAATTTTACTTCCCTATT |
| rs74316246 | ACGTTGGATGCTTTCCTTTGCACCATGTGA | ACGTTGGATGCACTTGAGAGCCATACAAGACA | 8161.3 | ggacGTATCCAGTTTACTTCTTCCCAC |
| rs75890454 | ACGTTGGATGGCCAAGGCTGCTTATGTGA | ACGTTGGATGGGGTTGATCCTCATTACTGTTTG | 7873.2 | caTCATCACCTATTGGCTATAAAATC |
| rs77877520 | ACGTTGGATGTGAAGCTGTTTTTGTGCTCT | ACGTTGGATGTTGTGAAACTTTTGTCTAGTTTTGC | 7041.6 | tgagCCCATTACAGACAGCGTAA |
| rs854560 | ACGTTGGATGGGATCCACATCCTGCAATAA | ACGTTGGATGTGGGCATGGGTATACAGAAA | 4890.2 | AACTGGCTCTGAAGAC |
