# Supplementary material for: Gene-Based Sequencing Identifies Lipid-Influencing Variants with Ethnicity-Specific Effects in African Americans
Source: PLoS Genet. 2014 Mar 6;10(3):e1004190. doi: 10.1371/journal.pgen.1004190 (PMC3945436; doi:10.1371/journal.pgen.1004190)
Supplement: Table S5 — Description of included variants. Description of all variants included in the genotyping stage of analysis. (DOCX) [file pgen.1004190.s005.docx]

**Table S5.** Description of Included Variants

| **Marker** | **Gene** | **Chr** | **Position^1^** | **Ref/Var**  **Allele** | **MAF** | **1000 Genomes^2^** | | **Type** |
| --- | --- | --- | --- | --- | --- | --- | --- | --- |
|  |  |  |  |  |  | **AFR** | **EUR** |  |
| rs3917582 | *PON1* | 7 | 94927097 | C/T | 0.04 | 0.04 | 0 | 3' |
| rs201585300 | *PON1* | 7 | 94927137 | T/C | 0.01 | - | - | 3' |
| rs112956781 | *PON1* | 7 | 94927207 | C/CAAAT | 0.05 | 0.07 | 0 | 3' |
| rs854550 | *PON1* | 7 | 94927242 | T/C | 0.21 | 0.78 | 0.81 | 3' |
| rs3832528 | *PON1* | 7 | 94927340 | CCTTT/C | 0.13 | 0.16 | 0.06 | 3' |
| rs3917579 | *PON1* | 7 | 94927343 | T/A | 0.02 | - | - | 3' |
| rs199700441 | *PON1* | 7 | 94927350 | A/AT | 0.01 | 0.01 | 0.003 | 3' |
| rs3735590 | *PON1* | 7 | 94927495 | G/A | 0.29 | 0.33 | 0.07 | 3' |
| rs854551 | *PON1* | 7 | 94927677 | A/G | 0.26 | 0.72 | 0.81 | 3' |
| rs3917577 | *PON1* | 7 | 94927708 | T/C | 0.1 | 0.09 | 0.11 | 3' |
| 7.949278641 | *PON1* | 7 | 94927864 | C/T | 0.01 | - | - | 3' |
| rs3917576 | *PON1* | 7 | 94927865 | C/T | 0.12 | 0.15 | 0.003 | 3' |
| rs854552 | *PON1* | 7 | 94927924 | C/T | 0.43 | 0.38 | 0.74 | 3' |
| rs3917575 | *PON1* | 7 | 94927996 | A/G | 0.04 | 0.03 | 0 | 3' |
| rs78414794 | *PON1* | 7 | 94928554 | AAC/A | 0.13 | 0.16 | 0.06 | Intron |
| rs854553 | *PON1* | 7 | 94928746 | A/G | 0.45 | 0.4 | 0.74 | Intron |
| rs184277527 | *PON1* | 7 | 94928796 | T/C | 0.01 | 0.01 | 0 | Intron |
| rs59744786 | *PON1* | 7 | 94928829 | A/C | 0.02 | 0.02 | 0 | Intron |
| rs854554 | *PON1* | 7 | 94928977 | A/G | 0.36 | 0.61 | 0.8 | Intron |
| rs148907362 | *PON1* | 7 | 94928985 | G/A | 0.01 | 0.01 | 0 | Intron |
| rs3917572 | *PON1* | 7 | 94929190 | A/G | 0.19 | 0.22 | 0.06 | Intron |
| rs3917571 | *PON1* | 7 | 94929341 | CAT/C | 0.13 | 0.15 | 0.0026 | Intron |
| rs3917569 | *PON1* | 7 | 94929439 | T/C | 0.15 | 0.17 | 0.06 | Intron |
| rs77399317 | *PON1* | 7 | 94929875 | T/C | 0.01 | 0.03 | 0 | Intron |
| rs3917568 | *PON1* | 7 | 94930012 | C/A | 0.03 | 0.03 | 0 | Intron |
| rs3917567 | *PON1* | 7 | 94930085 | T/C | 0.14 | 0.16 | 0.06 | Intron |
| rs2237580 | *PON1* | 7 | 94930108 | T/G | 0.07 | 0.09 | 0.11 | Intron |
| rs854555 | *PON1* | 7 | 94930391 | A/C | 0.32 | 0.25 | 0.64 | Intron |
| rs7792044 | *PON1* | 7 | 94931271 | T/C | 0.14 | 0.18 | 0.08 | Intron |
| rs7809060 | *PON1* | 7 | 94931387 | C/T | 0.14 | 0.17 | 0.07 | Intron |
| rs148911901 | *PON1* | 7 | 94931560 | A/T | 0.002 | - | - | NonSyn |
| rs3917564 | *PON1* | 7 | 94931712 | T/C | 0.14 | 0.17 | 0.01 | Intron |
| rs151197485 | *PON1* | 7 | 94931944 | G/GA | 0.29 | 0.31 | 0.16 | Intron |
| rs73433158 | *PON1* | 7 | 94932509 | A/G | 0.02 | 0.02 | 0 | Intron |
| rs116614917 | *PON1* | 7 | 94932550 | C/T | 0.02 | 0.02 | 0 | Intron |
| rs3917562 | *PON1* | 7 | 94932664 | ATGCT/A | 0.43 | 0.5 | 0.17 | Intron |
| rs202212568 | *PON1* | 7 | 94932669 | TGC/T | 0.28 | 0.3 | 0.15 | Intron |
| rs3917558 | *PON1* | 7 | 94932904 | T/C | 0.39 | 0.49 | 0.07 | Intron |
| rs3917556 | *PON1* | 7 | 94933189 | T/C | 0.14 | 0.17 | 0.06 | Intron |
| rs3917555 | *PON1* | 7 | 94933611 | T/C | 0.03 | 0.03 | 0 | Intron |
| rs2237581 | *PON1* | 7 | 94933833 | A/G | 0.1 | 0.1 | 0.11 | Intron |
| rs3917553 | *PON1* | 7 | 94934089 | C/T | 0.02 | 0.02 | 0.0026 | Intron |
| rs2237582 | *PON1* | 7 | 94934200 | G/A | 0.31 | 0.77 | 0.29 | Intron |
| rs3917552 | *PON1* | 7 | 94934259 | T/G | 0.14 | 0.11 | 0 | Intron |
| rs3917551 | *PON1* | 7 | 94934455 | C/T | 0.14 | 0.17 | 0.06 | Intron |
| rs3917550 | *PON1* | 7 | 94934573 | C/T | 0.13 | 0.1 | 0.12 | Intron |
| rs116700165 | *PON1* | 7 | 94934686 | G/A | 0.01 | 0.01 | 0 | Intron |
| rs3917549 | *PON1* | 7 | 94935198 | CA/C | 0.46 | 0.62 | 0.18 | Intron |
| rs73433162 | *PON1* | 7 | 94935378 | A/G | 0.02 | 0.02 | 0 | Intron |
| rs3917548 | *PON1* | 7 | 94935905 | A/G | 0.17 | 0.2 | 0.06 | Intron |
| rs2269829 | *PON1* | 7 | 94936129 | A/G | 0.46 | 0.58 | 0.29 | Intron |
| rs3917545 | *PON1* | 7 | 94936235 | A/C | 0.13 | 0.1 | 0.12 | Intron |
| rs3917543 | *PON1* | 7 | 94936569 | G/C | 0.15 | 0.17 | 0 | Intron |
| rs3917542 | *PON1* | 7 | 94936692 | C/T | 0.39 | 0.39 | 0.22 | Intron |
| rs662 | *PON1* | 7 | 94937446 | C/T | 0.32 | 0.75 | 0.29 | NonSyn |
| rs3917541 | *PON1* | 7 | 94937624 | C/T | 0.17 | 0.2 | 0.06 | Intron |
| rs74632005 | *PON1* | 7 | 94937696 | T/TAA | 0.33 | 0.75 | 0.29 | Intron |
| rs3917538 | *PON1* | 7 | 94937893 | G/A | 0.5 | 0.55 | 0.23 | Intron |
| rs2158155 | *PON1* | 7 | 94938176 | G/A | 0.13 | 0.17 | 0.06 | Intron |
| rs2057681 | *PON1* | 7 | 94938257 | C/T | 0.31 | 0.77 | 0.29 | Intron |
| rs3917537 | *PON1* | 7 | 94938361 | C/T | 0.12 | 0.17 | 0.01 | Intron |
| rs2299255 | *PON1* | 7 | 94938786 | T/C | 0.13 | 0.1 | 0.12 | Intron |
| rs2299256 | *PON1* | 7 | 94939032 | C/A | 0.48 | 0.55 | 0.23 | Intron |
| rs3917536 | *PON1* | 7 | 94939301 | C/A | 0.13 | 0.17 | 0.01 | Intron |
| rs141981682 | *PON1* | 7 | 94939587 | ATTAG/A | 0.04 | 0.06 | 0 | Intron |
| rs3917534 | *PON1* | 7 | 94939797 | T/C | 0.31 | 0.77 | 0.29 | Intron |
| rs3917533 | *PON1* | 7 | 94939830 | G/A | 0.39 | 0.38 | 0.22 | Intron |
| rs3917532 | *PON1* | 7 | 94940119 | A/T | 0.33 | 0.75 | 0.29 | Intron |
| rs3917531 | *PON1* | 7 | 94940122 | C/T | 0.26 | 0.28 | 0.11 | Intron |
| rs3917529 | *PON1* | 7 | 94940235 | A/G | 0.33 | 0.75 | 0.29 | Intron |
| rs3917528 | *PON1* | 7 | 94940240 | C/G | 0.26 | 0.28 | 0.11 | Intron |
| rs3917527 | *PON1* | 7 | 94940258 | T/C | 0.13 | 0.18 | 0.06 | Intron |
| rs3917526 | *PON1* | 7 | 94940281 | A/G | 0.03 | 0.04 | 0 | Intron |
| rs3917525 | *PON1* | 7 | 94940478 | TG/T | 0.38 | 0.39 | 0.3 | Intron |
| rs2074354 | *PON1* | 7 | 94940587 | G/A | 0.25 | 0.28 | 0.11 | Intron |
| rs116559253 | *PON1* | 7 | 94940590 | T/A | 0.02 | 0.03 | 0 | Intron |
| rs3917524 | *PON1* | 7 | 94940598 | G/T | 0.13 | 0.17 | 0.01 | Intron |
| rs1157745 | *PON1* | 7 | 94941038 | T/G | 0.34 | 0.75 | 0.29 | Intron |
| rs3917521 | *PON1* | 7 | 94941415 | G/A | 0.38 | 0.38 | 0.22 | Intron |
| rs3917519 | *PON1* | 7 | 94941622 | C/T | 0.02 | 0.03 | 0 | Intron |
| rs3917518 | *PON1* | 7 | 94941781 | C/A | 0.14 | 0.17 | 0.06 | Intron |
| rs3917517 | *PON1* | 7 | 94941820 | C/T | 0.13 | 0.17 | 0.01 | Intron |
| rs3917515 | *PON1* | 7 | 94942083 | G/A | 0.13 | 0.17 | 0.06 | Intron |
| rs3917514 | *PON1* | 7 | 94942479 | G/A | 0.11 | 0.15 | 0.01 | Intron |
| rs3917513 | *PON1* | 7 | 94942504 | A/T | 0.02 | 0.03 | 0 | Intron |
| rs143463533 | *PON1* | 7 | 94942739 | C/A | 0.01 | 0.01 | 0 | Intron |
| rs2299257 | *PON1* | 7 | 94942765 | A/C | 0.4 | 0.65 | 0.4 | Intron |
| rs2299258 | *PON1* | 7 | 94942917 | G/A | 0.06 | 0.04 | 0.12 | Intron |
| rs10593904 | *PON1* | 7 | 94942932 | G/GTA | 0.42 | - | - | Intron |
| rs3917510 | *PON1* | 7 | 94943120 | T/G | 0.12 | 0.15 | 0.05 | Intron |
| rs3917509 | *PON1* | 7 | 94943123 | T/G | 0.09 | 0.11 | 0.05 | Intron |
| rs2299259 | *PON1* | 7 | 94943397 | G/A | 0.18 | 0.14 | 0.29 | Intron |
| rs3917506 | *PON1* | 7 | 94944183 | GA/G | 0.18 | 0.14 | 0.28 | Intron |
| rs854556 | *PON1* | 7 | 94944923 | C/T | 0.17 | 0.15 | 0.35 | Intron |
| rs854557 | *PON1* | 7 | 94945215 | T/G | 0.17 | 0.15 | 0.35 | Intron |
| rs854558 | *PON1* | 7 | 94945374 | T/C | 0.33 | 0.67 | 0.7 | Intron |
| rs3917503 | *PON1* | 7 | 94945453 | C/T | 0.5 | 0.52 | 0.35 | Intron |
| rs2301711 | *PON1* | 7 | 94945659 | T/C | 0.31 | 0.39 | 0.06 | Intron |
| rs3917502 | *PON1* | 7 | 94945744 | C/T | 0.13 | 0.12 | 0 | Intron |
| rs3917501 | *PON1* | 7 | 94945829 | A/G | 0.04 | 0.06 | 0 | Intron |
| rs3917500 | *PON1* | 7 | 94945843 | A/G | 0.08 | 0.08 | 0 | Intron |
| rs854559 | *PON1* | 7 | 94945872 | G/A | 0.17 | 0.15 | 0.35 | Intron |
| rs3917499 | *PON1* | 7 | 94945887 | T/C | 0.08 | 0.08 | 0 | Intron |
| rs854560 | *PON1* | 7 | 94946084 | A/T | 0.16 | 0.15 | 0.35 | NonSyn |
| rs3917498 | *PON1* | 7 | 94946255 | T/G | 0.5 | 0.52 | 0.35 | Intron |
| rs705378 | *PON1* | 7 | 94946571 | G/T | 0.17 | 0.15 | 0.35 | Intron |
| rs62467349 | *PON1* | 7 | 94946795 | C/T | 0.12 | 0.08 | 0.11 | Intron |
| rs854561 | *PON1* | 7 | 94947017 | C/T | 0.17 | 0.15 | 0.35 | Intron |
| rs2074351 | *PON1* | 7 | 94947799 | G/A | 0.18 | 0.16 | 0.29 | Intron |
| rs3917494 | *PON1* | 7 | 94947925 | A/C | 0.02 | 0.02 | 0 | Intron |
| rs854562 | *PON1* | 7 | 94947969 | C/T | 0.09 | 0.04 | 0.3 | Intron |
| rs854563 | *PON1* | 7 | 94948009 | G/A | 0.16 | 0.13 | 0.35 | Intron |
| rs3917493 | *PON1* | 7 | 94948028 | T/C | 0.12 | 0.16 | 0.06 | Intron |
| rs3917492 | *PON1* | 7 | 94948114 | C/G | 0.02 | 0.03 | 0 | Intron |
| rs854564 | *PON1* | 7 | 94948182 | G/T | 0.34 | 0.67 | 0.7 | Intron |
| rs854565 | *PON1* | 7 | 94948344 | A/G | 0.34 | 0.67 | 0.7 | Intron |
| rs2272365 | *PON1* | 7 | 94948626 | A/C | 0.04 | 0.02 | 0.17 | Intron |
| rs854566 | *PON1* | 7 | 94948749 | A/G | 0.3 | 0.68 | 0.81 | Intron |
| rs854567 | *PON1* | 7 | 94948784 | A/G | 0.3 | 0.68 | 0.81 | Intron |
| rs3917490 | *PON1* | 7 | 94948841 | C/T | 0.47 | 0.53 | 0.5 | Intron |
| rs3917488 | *PON1* | 7 | 94949073 | A/G | 0.08 | 0.08 | 0 | Intron |
| rs2049649 | *PON1* | 7 | 94949329 | A/G | 0.41 | 0.46 | 0.36 | Intron |
| rs2299260 | *PON1* | 7 | 94949537 | T/C | 0.31 | 0.32 | 0.18 | Intron |
| rs2299261 | *PON1* | 7 | 94949663 | A/G | 0.35 | 0.42 | 0.35 | Intron |
| rs854568 | *PON1* | 7 | 94949801 | G/A | 0.48 | 0.5 | 0.78 | Intron |
| rs2299262 | *PON1* | 7 | 94949928 | C/T | 0.26 | 0.24 | 0.42 | Intron |
| rs854569 | *PON1* | 7 | 94950055 | T/G | 0.33 | 0.22 | 0.78 | Intron |
| rs2237583 | *PON1* | 7 | 94950177 | C/T | 0.12 | 0.05 | 0.3 | Intron |
| rs77317494 | *PON1* | 7 | 94950181 | G/A | 0.03 | 0.02 | 0 | Intron |
| rs78870097 | *PON1* | 7 | 94950606 | G/A | 0.01 | 0.02 | 0 | Intron |
| rs3917481 | *PON1* | 7 | 94950765 | C/T | 0.09 | 0.06 | 0.02 | Intron |
| rs2237584 | *PON1* | 7 | 94950837 | C/T | 0.02 | 0.01 | 0.07 | Intron |
| rs3917478 | *PON1* | 7 | 94951569 | T/C | 0.07 | 0.06 | 0.13 | Intron |
| rs5885907 | *PON1* | 7 | 94951617 | GT/G | 0.3 | 0.24 | 0.63 | Intron |
| rs201537582 | *PON1* | 7 | 94951638 | ATGTAT  GTG/A | 0.12 | 0.15 | 0.14 | Intron |
| rs3917477 | *PON1* | 7 | 94951966 | A/G | 0.13 | 0.17 | 0.03 | Intron |
| rs3917476 | *PON1* | 7 | 94952093 | G/T | 0.13 | 0.17 | 0.03 | Intron |
| rs854570 | *PON1* | 7 | 94952692 | C/A | 0.27 | 0.22 | 0.64 | Intron |
| rs3917469 | *PON1* | 7 | 94952816 | T/G | 0.06 | 0.03 | 0.13 | Intron |
| rs3917468 | *PON1* | 7 | 94952924 | T/G | 0.04 | 0.02 | 0.13 | Intron |
| rs113688396 | *PON1* | 7 | 94953274 | T/A | 0.02 | 0.01 | 0 | Intron |
| rs705379 | *PON1* | 7 | 94953895 | G/A | 0.15 | 0.08 | 0.49 | 5' |
| rs2227631 | *SERPINE1* | 7 | 100769538 | G/A | 0.27 | 0.76 | 0.39 | 5' |
| rs1799762 | *SERPINE1* | 7 | 100769706 | T/TG | 0.27 | - | - | 5' |
| rs2227632 | *SERPINE1* | 7 | 100769875 | G/A | 0.03 | 0.05 | 0 | 5' |
| rs2227636 | *SERPINE1* | 7 | 100770617 | G/A | 0.03 | 0.05 | 0 | Intron |
| rs2227637 | *SERPINE1* | 7 | 100770743 | G/A | 0.05 | 0.06 | 0 | Intron |
| rs2227638 | *SERPINE1* | 7 | 100770906 | T/C | 0.02 | 0.07 | 0 | Intron |
| rs2227639 | *SERPINE1* | 7 | 100770908 | A/T | 0.06 | 0.09 | 0.02 | Intron |
| rs2227640 | *SERPINE1* | 7 | 100771059 | G/A | 0.03 | 0.05 | 0 | Intron |
| rs145565109 | *SERPINE1* | 7 | 100771580 | CAAG/C | 0.03 | 0.06 | 0 | Intron |
| rs6092 | *SERPINE1* | 7 | 100771717 | G/A | 0.01 | 0.0041 | 0.09 | NonSyn |
| rs6090 | *SERPINE1* | 7 | 100771723 | G/A | 0.05 | 0.07 | 0.02 | NonSyn |
| rs2227648 | *SERPINE1* | 7 | 100772173 | A/T | 0.04 | 0.08 | 0 | Intron |
| rs2227649 | *SERPINE1* | 7 | 100772242 | CAA/C | 0.02 | 0.04 | 0 | Intron |
| rs2227650 | *SERPINE1* | 7 | 100772275 | A/C | 0.03 | 0.06 | 0 | Intron |
| rs2227651 | *SERPINE1* | 7 | 100772304 | C/T | 0.05 | 0.09 | 0.0026 | Intron |
| rs2227652 | *SERPINE1* | 7 | 100772305 | A/G | 0.05 | 0.09 | 0.01 | Intron |
| rs186618160 | *SERPINE1* | 7 | 100772322 | C/T | 0.03 | 0.05 | 0 | Intron |
| rs7788294 | *SERPINE1* | 7 | 100772630 | C/T | 0.14 | 0.82 | 0.86 | Intron |
| rs114725937 | *SERPINE1* | 7 | 100772794 | C/T | 0.08 | 0.14 | 0 | Intron |
| rs6959054 | *SERPINE1* | 7 | 100772817 | C/T | 0.09 | 0.12 | 0 | Intron |
| rs3020623 | *SERPINE1* | 7 | 100772845 | G/A | 0.24 | 0.22 | 0.39 | Intron |
| rs114742909 | *SERPINE1* | 7 | 100772886 | G/A | 0.07 | 0.1 | 0 | Intron |
| rs2227657 | *SERPINE1* | 7 | 100773459 | T/C | 0.1 | 0.88 | 0.87 | Intron |
| rs2227659 | *SERPINE1* | 7 | 100774144 | TA/T | 0.03 | 0.06 | 0 | Intron |
| rs2227660 | *SERPINE1* | 7 | 100774275 | G/A | 0.13 | 0.82 | 0.87 | Intron |
| rs2227663 | *SERPINE1* | 7 | 100774557 | A/G | 0.1 | 0.88 | 0.87 | Intron |
| rs2227667 | *SERPINE1* | 7 | 100774749 | A/G | 0.23 | 0.26 | 0.21 | Intron |
| rs2227668 | *SERPINE1* | 7 | 100774855 | A/G | 0.11 | 0.83 | 0.85 | Intron |
| rs145904319 | *SERPINE1* | 7 | 100774979 | C/T | 0.07 | 0.09 | 0 | Intron |
| rs41281004 | *SERPINE1* | 7 | 100775018 | C/T | 0.07 | 0.1 | 0.02 | Intron |
| rs2227672 | *SERPINE1* | 7 | 100775686 | G/T | 0.01 | 0.02 | 0.12 | Intron |
| rs2227673 | *SERPINE1* | 7 | 100775986 | A/G | 0.49 | 0.42 | 0.44 | Intron |
| rs2227674 | *SERPINE1* | 7 | 100776208 | A/G | 0.27 | 0.3 | 0.21 | Intron |
| rs2227676 | *SERPINE1* | 7 | 100776230 | A/T | 0.02 | 0.02 | 0.2 | Intron |
| rs2227677 | *SERPINE1* | 7 | 100776254 | G/T | 0.1 | 0.1 | 0 | Intron |
| rs2227679 | *SERPINE1* | 7 | 100776302 | C/T | 0.49 | 0.41 | 0.45 | Intron |
| rs2227682 | *SERPINE1* | 7 | 100776635 | C/T | 0.11 | 0.1 | 0 | Intron |
| rs2227683 | *SERPINE1* | 7 | 100776636 | G/A | 0.03 | 0.04 | 0.2 | Intron |
| rs1029528 | *SERPINE1* | 7 | 100776674 | C/T | 0.02 | 0.03 | 0.2 | Intron |
| rs72004331 | *SERPINE1* | 7 | 100776745 | CAGAG/C | 0.49 | 0.44 | 0.45 | Intron |
| rs2227684 | *SERPINE1* | 7 | 100776931 | G/A | 0.49 | 0.44 | 0.45 | Intron |
| rs2070682 | *SERPINE1* | 7 | 100777267 | T/C | 0.5 | 0.44 | 0.45 | Intron |
| rs2227686 | *SERPINE1* | 7 | 100778324 | A/G | 0.49 | 0.42 | 0.45 | Intron |
| rs2227687 | *SERPINE1* | 7 | 100778327 | C/T | 0.49 | 0.42 | 0.45 | Intron |
| rs2227690 | *SERPINE1* | 7 | 100778622 | A/G | 0.04 | 0.05 | 0.12 | Intron |
| rs2227692 | *SERPINE1* | 7 | 100779244 | C/T | 0.15 | 0.17 | 0.09 | Intron |
| rs2070683 | *SERPINE1* | 7 | 100779941 | A/T | 0.49 | 0.41 | 0.45 | Intron |
| rs2227694 | *SERPINE1* | 7 | 100780176 | A/G | 0.09 | 0.88 | 0.87 | Intron |
| rs2227696 | *SERPINE1* | 7 | 100780890 | C/T | 0.02 | 0.0041 | 0 | 3' |
| rs2227698 | *SERPINE1* | 7 | 100781468 | C/A | 0.06 | 0.03 | 0 | 3' |
| rs2227714 | *SERPINE1* | 7 | 100781909 | C/T | 0.01 | 0.0041 | 0.05 | 3' |
| rs1050955 | *SERPINE1* | 7 | 100782460 | C/T | 0.22 | 0.25 | 0.21 | 3' |
| rs1800590 | *LPL* | 8 | 19796671 | T/G | 0.36 | 0.44 | 0.01 | 5' |
| rs75890454 | *LPL* | 8 | 19796688 | T/A | 0.01 | 0.02 | 0 | 5' |
| rs145405273 | *LPL* | 8 | 19796845 | G/A | 0.002 | - | - | Syn |
| rs3779787 | *LPL* | 8 | 19797916 | G/T | 0.05 | 0.04 | 0.14 | Intron |
| rs146786419 | *LPL* | 8 | 19798339 | C/T | 0.03 | 0.04 | 0 | Intron |
| rs185670596 | *LPL* | 8 | 19798411 | G/C | 0.04 | 0.04 | 0 | Intron |
| rs150730448 | *LPL* | 8 | 19798438 | T/TA | 0.2 | - | - | Intron |
| rs34309063 | *LPL* | 8 | 19799139 | G/A | 0.11 | 0.11 | 0.24 | Intron |
| rs115668974 | *LPL* | 8 | 19799306 | C/T | 0.03 | 0.05 | 0.0013 | Intron |
| rs17410577 | *LPL* | 8 | 19799545 | G/C | 0.11 | 0.11 | 0.24 | Intron |
| rs1534649 | *LPL* | 8 | 19799641 | G/T | 0.18 | 0.89 | 0.45 | Intron |
| rs73667466 | *LPL* | 8 | 19799805 | G/A | 0.02 | 0.03 | 0 | Intron |
| rs13266204 | *LPL* | 8 | 19800005 | A/G | 0.06 | 0.05 | 0.24 | Intron |
| rs114624068 | *LPL* | 8 | 19800298 | G/T | 0.02 | 0.03 | 0.0026 | Intron |
| rs6997330 | *LPL* | 8 | 19800529 | G/C | 0.21 | 0.29 | 0.01 | Intron |
| rs1031045 | *LPL* | 8 | 19801112 | G/A | 0.34 | 0.4 | 0.01 | Intron |
| rs60633545 | *LPL* | 8 | 19801353 | A/G | 0.34 | 0.4 | 0.01 | Intron |
| rs112127208 | *LPL* | 8 | 19801530 | T/G | 0.05 | 0.05 | 0.14 | Intron |
| rs59254395 | *LPL* | 8 | 19802134 | C/T | 0.17 | 0.21 | 0.01 | Intron |
| rs56043715 | *LPL* | 8 | 19802135 | A/G | 0.22 | 0.3 | 0.01 | Intron |
| rs61274012 | *LPL* | 8 | 19802343 | T/C | 0.03 | 0.05 | 0 | Intron |
| rs10104051 | *LPL* | 8 | 19802402 | C/T | 0.18 | 0.88 | 0.44 | Intron |
| rs73667467 | *LPL* | 8 | 19802419 | G/A | 0.02 | 0.03 | 0 | Intron |
| rs28615996 | *LPL* | 8 | 19802711 | T/C | 0.25 | 0.33 | 0.01 | Intron |
| rs28645722 | *LPL* | 8 | 19802894 | G/A | 0.21 | 0.28 | 0.01 | Intron |
| rs28575919 | *LPL* | 8 | 19802969 | C/G | 0.19 | 0.25 | 0.01 | Intron |
| rs114437971 | *LPL* | 8 | 19802988 | T/G | 0.04 | 0.07 | 0 | Intron |
| rs6999612 | *LPL* | 8 | 19803084 | T/C | 0.2 | 0.25 | 0.01 | Intron |
| rs3779788 | *LPL* | 8 | 19803093 | C/T | 0.05 | 0.04 | 0.13 | Intron |
| rs59811201 | *LPL* | 8 | 19803137 | T/C | 0.12 | 0.13 | 0.01 | Intron |
| rs7000460 | *LPL* | 8 | 19803802 | A/C | 0.21 | 0.28 | 0.01 | Intron |
| rs56321069 | *LPL* | 8 | 19803996 | T/A | 0.38 | 0.66 | 0.21 | Intron |
| rs114186305 | *LPL* | 8 | 19804163 | C/T | 0.02 | 0.03 | 0 | Intron |
| rs73601656 | *LPL* | 8 | 19804584 | G/A | 0.08 | 0.07 | 0 | Intron |
| rs28445964 | *LPL* | 8 | 19804596 | A/G | 0.18 | 0.25 | 0.01 | Intron |
| rs145257746 | *LPL* | 8 | 19804813 | T/G | 0.02 | 0.03 | 0.0013 | Intron |
| rs28689946 | *LPL* | 8 | 19804992 | A/C | 0.2 | 0.27 | 0.01 | Intron |
| rs28582042 | *LPL* | 8 | 19804999 | G/A | 0.2 | 0.27 | 0.01 | Intron |
| rs73667468 | *LPL* | 8 | 19805277 | G/T | 0.2 | 0.27 | 0.01 | Intron |
| rs73667469 | *LPL* | 8 | 19805495 | T/G | 0.22 | 0.29 | 0.01 | Intron |
| rs77298438 | *LPL* | 8 | 19805536 | C/G | 0.03 | 0.04 | 0 | Intron |
| rs1801177 | *LPL* | 8 | 19805708 | G/A | 0.01 | 0.04 | 0.01 | NonSyn |
| rs11542065 | *LPL* | 8 | 19805815 | C/G | 0.01 | 0.02 | 0 | NonSyn |
| rs59054859 | *LPL* | 8 | 19805929 | A/G | 0.08 | 0.08 | 0 | Intron |
| rs6991305 | *LPL* | 8 | 19806381 | G/A | 0.03 | 0.04 | 0 | Intron |
| rs137999837 | *LPL* | 8 | 19806406 | A/T | 0.01 | 0.02 | 0 | Intron |
| rs140116605 | *LPL* | 8 | 19806512 | CTG/C | 0.11 | 0.11 | 0 | Intron |
| rs74377536 | *LPL* | 8 | 19806568 | C/A | 0.02 | 0.01 | 0.1 | Intron |
| rs7016529 | *LPL* | 8 | 19806631 | T/C | 0.21 | 0.28 | 0.01 | Intron |
| rs8176337 | *LPL* | 8 | 19806671 | C/G | 0.38 | 0.66 | 0.21 | Intron |
| rs73667470 | *LPL* | 8 | 19807341 | A/C | 0.35 | 0.4 | 0.01 | Intron |
| rs149017698 | *LPL* | 8 | 19807462 | G/GA | 0.3 | 0.34 | 0.01 | Intron |
| rs73667471 | *LPL* | 8 | 19807941 | A/C | 0.02 | 0.03 | 0 | Intron |
| rs74304285 | *LPL* | 8 | 19808030 | G/A | 0.09 | 0.1 | 0.13 | Intron |
| rs57357723 | *LPL* | 8 | 19808038 | C/T | 0.11 | 0.12 | 0.01 | Intron |
| rs113023641 | *LPL* | 8 | 19808131 | G/A | 0.05 | 0.05 | 0.13 | Intron |
| rs80181352 | *LPL* | 8 | 19808584 | G/T | 0.04 | 0.04 | 0.06 | Intron |
| rs79760154 | *LPL* | 8 | 19808683 | A/C | 0.04 | 0.08 | 0 | Intron |
| rs80341714 | *LPL* | 8 | 19808687 | C/T | 0.03 | 0.03 | 0 | Intron |
| rs7002728 | *LPL* | 8 | 19808737 | G/T | 0.1 | 0.11 | 0 | Intron |
| rs114236319 | *LPL* | 8 | 19808892 | G/A | 0.02 | 0.05 | 0 | Intron |
| rs1121923 | *LPL* | 8 | 19809435 | G/A | 0.11 | 0.15 | 0.04 | Syn |
| rs73667472 | *LPL* | 8 | 19809695 | T/C | 0.15 | 0.15 | 0.08 | Intron |
| rs58670071 | *LPL* | 8 | 19809794 | C/A | 0.05 | 0.04 | 0 | Intron |
| rs75026342 | *LPL* | 8 | 19809822 | C/T | 0.08 | 0.08 | 0 | Intron |
| rs57345602 | *LPL* | 8 | 19810696 | A/C | 0.02 | 0.03 | 0 | Intron |
| rs247 | *LPL* | 8 | 19810791 | A/C | 0.06 | 0.04 | 0 | Intron |
| rs248 | *LPL* | 8 | 19810826 | G/A | 0.02 | 0.04 | 0.06 | Syn |
| rs249 | *LPL* | 8 | 19811006 | T/C | 0.13 | 0.14 | 0.07 | Intron |
| rs142517359 | *LPL* | 8 | 19811023 | A/ATG | 0.36 | 0.4 | 0.13 | Intron |
| rs251 | *LPL* | 8 | 19811160 | T/C | 0.13 | 0.16 | 0 | Intron |
| rs252 | *LPL* | 8 | 19811231 | TA/T | 0.19 | 0.87 | 0.47 | Intron |
| rs202085673 | *LPL* | 8 | 19811234 | AT/A | 0.3 | 0.76 | 0.4 | Intron |
| rs253 | *LPL* | 8 | 19811417 | C/T | 0.28 | 0.76 | 0.47 | Intron |
| rs144254368 | *LPL* | 8 | 19811501 | C/A | 0.01 | 0.01 | 0 | Intron |
| rs254 | *LPL* | 8 | 19811897 | C/G | 0.22 | 0.21 | 0.14 | Intron |
| rs255 | *LPL* | 8 | 19811901 | T/C | 0.22 | 0.21 | 0.14 | Intron |
| rs80143795 | *LPL* | 8 | 19811944 | A/G | 0.08 | 0.08 | 0 | Intron |
| rs256 | *LPL* | 8 | 19811967 | C/T | 0.07 | 0.05 | 0.13 | Intron |
| rs257 | *LPL* | 8 | 19812228 | A/C | 0.03 | 0.06 | 0 | Intron |
| rs258 | *LPL* | 8 | 19812252 | G/C | 0.15 | 0.91 | 0.47 | Intron |
| rs259 | *LPL* | 8 | 19812437 | A/T | 0.04 | 0.06 | 0 | Intron |
| rs260 | *LPL* | 8 | 19812509 | C/G | 0.08 | 0.1 | 0 | Intron |
| rs261 | *LPL* | 8 | 19812620 | A/G | 0.17 | 0.19 | 0 | Intron |
| rs262 | *LPL* | 8 | 19812642 | A/T | 0.02 | 0.03 | 0 | Intron |
| rs263 | *LPL* | 8 | 19812812 | C/T | 0.34 | 0.37 | 0.18 | Intron |
| rs264 | *LPL* | 8 | 19813180 | G/A | 0.13 | 0.09 | 0.13 | Intron |
| rs266 | *LPL* | 8 | 19813299 | A/G | 0.12 | 0.15 | 0 | Intron |
| rs59184895 | *LPL* | 8 | 19813610 | T/C | 0.03 | 0.05 | 0 | Intron |
| rs269 | *LPL* | 8 | 19813667 | T/G | 0.4 | 0.45 | 0.19 | Intron |
| rs270 | *LPL* | 8 | 19813676 | C/A | 0.09 | 0.1 | 0.18 | Intron |
| rs271 | *LPL* | 8 | 19813702 | C/T | 0.07 | 0.04 | 0.14 | Intron |
| rs272 | *LPL* | 8 | 19813928 | C/G | 0.07 | 0.06 | 0 | Intron |
| rs147728402 | *LPL* | 8 | 19813969 | G/GT | 0.14 | 0.11 | 0.14 | Intron |
| rs58935878 | *LPL* | 8 | 19813976 | T/TA | 0.1 | 0.08 | 0.07 | Intron |
| rs276 | *LPL* | 8 | 19814289 | T/C | 0.14 | 0.16 | 0.02 | Intron |
| rs277 | *LPL* | 8 | 19814403 | T/C | 0.1 | 0.09 | 0.2 | Intron |
| rs278 | *LPL* | 8 | 19814523 | G/A | 0.08 | 0.08 | 0.2 | Intron |
| rs279 | *LPL* | 8 | 19814696 | C/G | 0.1 | 0.1 | 0.0013 | Intron |
| rs280 | *LPL* | 8 | 19814882 | G/A | 0.04 | 0.06 | 0.0013 | Intron |
| rs17091775 | *LPL* | 8 | 19814988 | G/A | 0.04 | 0.06 | 0 | Intron |
| rs281 | *LPL* | 8 | 19815023 | A/T | 0.42 | 0.46 | 0.31 | Intron |
| rs282 | *LPL* | 8 | 19815026 | C/G | 0.06 | 0.04 | 0.14 | Intron |
| rs283 | *LPL* | 8 | 19815098 | C/T | 0.45 | 0.52 | 0.23 | Intron |
| rs284 | *LPL* | 8 | 19815106 | T/C | 0.12 | 0.12 | 0.0013 | Intron |
| rs285 | *LPL* | 8 | 19815189 | C/T | 0.14 | 0.91 | 0.53 | Intron |
| rs287 | *LPL* | 8 | 19815556 | A/G | 0.29 | 0.27 | 0.26 | Intron |
| rs289 | *LPL* | 8 | 19815619 | T/C | 0.28 | 0.27 | 0.25 | Intron |
| rs140991223 | *LPL* | 8 | 19815623 | C/T | 0.08 | 0.08 | 0 | Intron |
| rs290 | *LPL* | 8 | 19815769 | G/A | 0.11 | 0.1 | 0.0013 | Intron |
| rs291 | *LPL* | 8 | 19815852 | T/C | 0.29 | 0.28 | 0.25 | Intron |
| rs149553676 | *LPL* | 8 | 19816078 | T/TA | 0.31 | 0.28 | 0.26 | Intron |
| rs294 | *LPL* | 8 | 19816125 | T/C | 0.14 | 0.14 | 0.13 | Intron |
| rs295 | *LPL* | 8 | 19816238 | A/C | 0.35 | 0.35 | 0.25 | Intron |
| rs297 | *LPL* | 8 | 19816371 | T/C | 0.3 | 0.28 | 0.25 | Intron |
| rs73601683 | *LPL* | 8 | 19816717 | G/A | 0.02 | 0.03 | 0 | Intron |
| rs300 | *LPL* | 8 | 19816887 | A/G | 0.02 | 0.01 | 0 | NonSyn |
| rs301 | *LPL* | 8 | 19816934 | T/C | 0.31 | 0.39 | 0.43 | Intron |
| rs303 | *LPL* | 8 | 19817279 | G/C | 0.1 | 0.09 | 0.12 | Intron |
| rs304 | *LPL* | 8 | 19817361 | T/G | 0.3 | 0.26 | 0.25 | Intron |
| rs305 | *LPL* | 8 | 19817401 | A/G | 0.3 | 0.26 | 0.25 | Intron |
| rs306 | *LPL* | 8 | 19817443 | G/A | 0.05 | 0.07 | 0 | Intron |
| rs307 | *LPL* | 8 | 19817455 | A/G | 0.11 | 0.12 | 0.0013 | Intron |
| rs310 | *LPL* | 8 | 19817546 | C/T | 0.07 | 0.06 | 0.12 | Intron |
| rs312 | *LPL* | 8 | 19817997 | G/C | 0.23 | 0.23 | 0.12 | Intron |
| rs313 | *LPL* | 8 | 19818026 | A/G | 0.11 | 0.12 | 0.0013 | Intron |
| rs314 | *LPL* | 8 | 19818042 | G/A | 0.34 | 0.7 | 0.31 | Intron |
| rs77434393 | *LPL* | 8 | 19818355 | G/A | 0.01 | 0.02 | 0 | Intron |
| rs316 | *LPL* | 8 | 19818436 | C/A | 0.21 | 0.21 | 0.12 | Syn |
| rs5934 | *LPL* | 8 | 19818551 | G/A | 0.02 | 0.03 | 0 | NonSyn |
| rs200854139 | *LPL* | 8 | 19818769 | GCA/G | 0.3 | 0.74 | 0.5 | Intron |
| rs318 | *LPL* | 8 | 19818969 | C/G | 0.11 | 0.13 | 0.04 | Intron |
| rs319 | *LPL* | 8 | 19818976 | A/C | 0.1 | 0.09 | 0.24 | Intron |
| rs320 | *LPL* | 8 | 19819077 | T/G | 0.31 | 0.27 | 0.27 | Intron |
| rs77243948 | *LPL* | 8 | 19819154 | T/C | 0.02 | 0.03 | 0.04 | Intron |
| rs322 | *LPL* | 8 | 19819217 | A/C | 0.39 | 0.39 | 0.27 | Intron |
| rs325 | *LPL* | 8 | 19819328 | T/C | 0.07 | 0.05 | 0.12 | Intron |
| rs326 | *LPL* | 8 | 19819439 | C/T | 0.46 | 0.58 | 0.32 | Intron |
| rs7005541 | *LPL* | 8 | 19819447 | C/G | 0.03 | 0.03 | 0.0013 | Intron |
| rs327 | *LPL* | 8 | 19819536 | A/C | 0.4 | 0.4 | 0.28 | Intron |
| rs328 | *LPL* | 8 | 19819724 | C/G | 0.07 | 0.05 | 0.12 | Stop |
| rs329 | *LPL* | 8 | 19820086 | A/G | 0.07 | 0.08 | 0.04 | Intron |
| rs330 | *LPL* | 8 | 19820396 | G/A | 0.1 | 0.09 | 0.15 | Intron |
| rs331 | *LPL* | 8 | 19820405 | G/A | 0.38 | 0.35 | 0.27 | Intron |
| rs138285812 | *LPL* | 8 | 19820410 | G/A | 0.03 | 0.03 | 0 | Intron |
| rs12679834 | *LPL* | 8 | 19820433 | T/C | 0.09 | 0.09 | 0.12 | Intron |
| rs76423146 | *LPL* | 8 | 19820480 | C/T | 0.13 | 0.17 | 0.03 | Intron |
| rs28681081 | *LPL* | 8 | 19820661 | G/A | 0.12 | 0.1 | 0.0013 | Intron |
| rs150647190 | *LPL* | 8 | 19820853 | G/A | 0.06 | 0.07 | 0 | Intron |
| rs117199990 | *LPL* | 8 | 19820916 | C/T | 0.07 | 0.05 | 0.12 | Intron |
| rs145391587 | *LPL* | 8 | 19820933 | A/C | 0.07 | 0.05 | 0.12 | Intron |
| rs28599962 | *LPL* | 8 | 19820995 | T/C | 0.11 | 0.1 | 0.0013 | Intron |
| rs17116619 | *LPL* | 8 | 19821233 | A/G | 0.08 | 0.12 | 0 | Intron |
| rs28439839 | *LPL* | 8 | 19821247 | G/A | 0.12 | 0.1 | 0.0013 | Intron |
| rs28424158 | *LPL* | 8 | 19821386 | C/T | 0.12 | 0.12 | 0.0013 | Intron |
| rs75278536 | *LPL* | 8 | 19821425 | T/G | 0.07 | 0.05 | 0.13 | Intron |
| rs201109344 | *LPL* | 8 | 19821465 | TG/T | 0.09 | 0.08 | 0.0013 | Intron |
| rs28716400 | *LPL* | 8 | 19821467 | G/T | 0.1 | 0.09 | 0.0013 | Intron |
| rs115078054 | *LPL* | 8 | 19821635 | G/A | 0.06 | 0.05 | 0 | Intron |
| rs77069344 | *LPL* | 8 | 19821782 | T/G | 0.07 | 0.05 | 0.13 | Intron |
| rs10099160 | *LPL* | 8 | 19821815 | T/G | 0.11 | 0.1 | 0.24 | Intron |
| rs75946927 | *LPL* | 8 | 19822041 | T/G | 0.07 | 0.07 | 0 | Intron |
| rs147750521 | *LPL* | 8 | 19822459 | T/TC | 0.12 | 0.12 | 0.0013 | Intron |
| rs147900112 | *LPL* | 8 | 19822613 | GC/G | 0.12 | 0.1 | 0.0013 | Intron |
| rs10283151 | *LPL* | 8 | 19822694 | A/G | 0.13 | 0.12 | 0.0013 | Intron |
| rs11570891 | *LPL* | 8 | 19822810 | C/T | 0.07 | 0.05 | 0.13 | Intron |
| rs4922115 | *LPL* | 8 | 19822830 | G/A | 0.11 | 0.1 | 0.14 | 3' |
| rs7818177 | *LPL* | 8 | 19822850 | G/A | 0.01 | 0.02 | 0 | 3' |
| rs3289 | *LPL* | 8 | 19823192 | T/C | 0.07 | 0.09 | 0.03 | 3' |
| rs11570892 | *LPL* | 8 | 19823617 | A/G | 0.22 | 0.26 | 0.14 | 3' |
| rs3208305 | *LPL* | 8 | 19823648 | A/T | 0.49 | 0.54 | 0.31 | 3' |
| rs1803924 | *LPL* | 8 | 19823674 | C/T | 0.06 | 0.05 | 0.13 | 3' |
| rs1059507 | *LPL* | 8 | 19823963 | C/T | 0.12 | 0.11 | 0.14 | 3' |
| rs150252331 | *LPL* | 8 | 19823988 | C/A | 0.0008 | 0.002 | 0.004 | 3' |
| rs3735964 | *LPL* | 8 | 19824045 | C/A | 0.06 | 0.05 | 0.13 | 3' |
| rs3200218 | *LPL* | 8 | 19824071 | A/G | 0.08 | 0.09 | 0.24 | 3' |
| rs139240067 | *LPL* | 8 | 19824112 | G/A | 0.02 | 0.01 | 0 | 3' |
| rs58998793 | *LPL* | 8 | 19824237 | T/C | 0.06 | 0.05 | 0 | 3' |
| rs13702 | *LPL* | 8 | 19824492 | T/C | 0.49 | 0.53 | 0.31 | 3' |
| rs1059611 | *LPL* | 8 | 19824563 | T/C | 0.18 | 0.16 | 0.13 | 3' |
| rs17091815 | *LPL* | 8 | 19824604 | A/T | 0.06 | 0.11 | 0 | 3' |
| rs149865365 | *LPL* | 8 | 19824626 | C/CTT | 0.18 | 0.17 | 0.13 | 3' |
| rs188554527 | *LPL* | 8 | 19824628 | G/T | 0.01 | 0.002 | 0 | 3' |
| rs15285 | *LPL* | 8 | 19824667 | C/T | 0.5 | 0.53 | 0.31 | 3' |
| rs3866471 | *LPL* | 8 | 19824669 | C/A | 0.22 | 0.24 | 0.14 | 3' |
| rs187374932 | *LPL* | 8 | 19824679 | A/G | 0.003 | 0.002 | 0 | 3' |
| rs3916027 | *LPL* | 8 | 19824868 | G/A | 0.44 | 0.41 | 0.27 | 3' |
| rs112644588 | *ABCA1* | 9 | 107526304 | A/G | 0.02 | 0.01 | 0 | 3' |
| rs116660941 | *ABCA1* | 9 | 107526427 | G/C | 0.03 | 0.06 | 0 | 3' |
| rs62565988 | *ABCA1* | 9 | 107526619 | G/A | 0.02 | 0.04 | 0.12 | 3' |
| rs147942567 | *ABCA1* | 9 | 107526663 | G/T | 0.03 | 0.01 | 0 | 3' |
| rs60974677 | *ABCA1* | 9 | 107526695 | CG/C | 0.11 | 0.1 | 0.0026 | 3' |
| rs10820725 | *ABCA1* | 9 | 107526769 | G/T | 0.05 | 0.03 | 0.18 | 3' |
| rs12552611 | *ABCA1* | 9 | 107527830 | T/C | 0.17 | 0.25 | 0.08 | 3' |
| rs2487714 | *ABCA1* | 9 | 107527916 | G/A | 0.2 | 0.21 | 0.51 | 3' |
| rs78973029 | *ABCA1* | 9 | 107528266 | G/A | 0.03 | 0.06 | 0 | 3' |
| rs141665560 | *ABCA1* | 9 | 107528655 | A/T | 0.02 | 0.04 | 0 | 3' |
| rs1559711 | *ABCA1* | 9 | 107529048 | T/G | 0.04 | 0.03 | 0.0013 | 3' |
| rs35350211 | *ABCA1* | 9 | 107529105 | T/C | 0.05 | 0.07 | 0.08 | 3' |
| rs2482426 | *ABCA1* | 9 | 107529411 | C/T | 0.13 | 0.12 | 0.43 | 3' |
| rs2162116 | *ABCA1* | 9 | 107529413 | C/T | 0.05 | 0.03 | 0.0013 | 3' |
| rs10991376 | *ABCA1* | 9 | 107529689 | T/C | 0.05 | 0.04 | 0.18 | 3' |
| rs114587458 | *ABCA1* | 9 | 107529957 | T/G | 0.03 | 0.06 | 0 | 3' |
| rs4743760 | *ABCA1* | 9 | 107530409 | A/C | 0.23 | 0.28 | 0.1 | 3' |
| rs10761083 | *ABCA1* | 9 | 107531005 | T/C | 0.05 | 0.03 | 0.18 | 3' |
| rs12351524 | *ABCA1* | 9 | 107531127 | C/T | 0.23 | 0.28 | 0.1 | 3' |
| rs10761084 | *ABCA1* | 9 | 107531152 | C/G | 0.05 | 0.03 | 0.18 | 3' |
| rs2472478 | *ABCA1* | 9 | 107531361 | T/C | 0.31 | 0.77 | 0.61 | 3' |
| rs2472477 | *ABCA1* | 9 | 107531658 | C/T | 0.33 | 0.72 | 0.58 | 3' |
| rs147905769 | *ABCA1* | 9 | 107531662 | A/G | 0.05 | 0.04 | 0.01 | 3' |
| rs2472476 | *ABCA1* | 9 | 107531956 | C/T | 0.31 | 0.77 | 0.61 | 3' |
| rs10820726 | *ABCA1* | 9 | 107532317 | A/G | 0.05 | 0.03 | 0.18 | 3' |
| rs73517826 | *ABCA1* | 9 | 107532369 | C/T | 0.19 | 0.2 | 0.0013 | 3' |
| rs2487715 | *ABCA1* | 9 | 107532394 | A/G | 0.16 | 0.9 | 0.61 | 3' |
| rs2482427 | *ABCA1* | 9 | 107532425 | C/G | 0.31 | 0.77 | 0.61 | 3' |
| rs139927095 | *ABCA1* | 9 | 107532481 | T/C | 0.03 | 0.01 | 0 | 3' |
| rs2472475 | *ABCA1* | 9 | 107532641 | T/C | 0.34 | 0.68 | 0.79 | 3' |
| rs141115300 | *ABCA1* | 9 | 107532775 | T/C | 0.03 | 0.03 | 0.0013 | 3' |
| rs3739740 | *ABCA1* | 9 | 107533159 | A/G | 0.05 | 0.03 | 0.0013 | 3' |
| rs3739741 | *ABCA1* | 9 | 107533175 | C/G | 0.05 | 0.03 | 0.18 | 3' |
| rs148201182 | *ABCA1* | 9 | 107533267 | G/A | 0.03 | 0.01 | 0 | 3' |
| rs114955285 | *ABCA1* | 9 | 107533433 | T/C | 0.02 | - | - | 3' |
| rs13439932 | *ABCA1* | 9 | 107533518 | C/T | 0.05 | 0.03 | 0.0013 | 3' |
| rs2487716 | *ABCA1* | 9 | 107533726 | G/A | 0.27 | 0.8 | 0.79 | 3' |
| rs10120882 | *ABCA1* | 9 | 107533808 | G/A | 0.03 | 0.03 | 0.0013 | 3' |
| rs2487717 | *ABCA1* | 9 | 107533906 | G/A | 0.36 | 0.41 | 0.58 | 3' |
| rs79734318 | *ABCA1* | 9 | 107533907 | C/A | 0.36 | 0.41 | 0.58 | 3' |
| rs10118769 | *ABCA1* | 9 | 107533911 | T/C | 0.07 | 0.08 | 0.01 | 3' |
| rs58746182 | *ABCA1* | 9 | 107534262 | A/C | 0.04 | 0.06 | 0 | 3' |
| rs137948744 | *ABCA1* | 9 | 107534465 | T/C | 0.03 | 0.03 | 0.0026 | 3' |
| rs140396016 | *ABCA1* | 9 | 107534588 | AATTAA  TATC/A | 0.42 | 0.63 | 0.58 | 3' |
| rs2472474 | *ABCA1* | 9 | 107534787 | T/C | 0.34 | 0.73 | 0.6 | 3' |
| rs2487718 | *ABCA1* | 9 | 107534906 | A/G | 0.41 | 0.45 | 0.78 | 3' |
| rs56270385 | *ABCA1* | 9 | 107535285 | A/G | 0.1 | 0.09 | 0 | 3' |
| rs28379689 | *ABCA1* | 9 | 107535492 | A/G | 0.08 | 0.07 | 0.01 | 3' |
| rs2482429 | *ABCA1* | 9 | 107535864 | C/G | 0.13 | 0.9 | 0.79 | 3' |
| rs10820730 | *ABCA1* | 9 | 107536022 | A/C | 0.3 | 0.35 | 0.34 | 3' |
| rs114615662 | *ABCA1* | 9 | 107536378 | G/A | 0.02 | 0.03 | 0 | 3' |
| rs2515606 | *ABCA1* | 9 | 107536895 | A/G | 0.12 | 0.12 | 0.44 | 3' |
| rs112148734 | *ABCA1* | 9 | 107536939 | C/T | 0.03 | 0.02 | 0 | 3' |
| rs10117535 | *ABCA1* | 9 | 107537021 | C/T | 0.38 | 0.71 | 0.35 | 3' |
| rs113533802 | *ABCA1* | 9 | 107537064 | A/T | 0.03 | 0.02 | 0 | 3' |
| rs35805725 | *ABCA1* | 9 | 107537087 | A/G | 0.03 | 0.02 | 0.07 | 3' |
| rs57365304 | *ABCA1* | 9 | 107537395 | C/A | 0.12 | 0.16 | 0.08 | 3' |
| rs74997200 | *ABCA1* | 9 | 107537574 | A/C | 0.04 | 0.03 | 0 | 3' |
| rs2472473 | *ABCA1* | 9 | 107537681 | G/A | 0.11 | 0.93 | 0.79 | 3' |
| rs114721211 | *ABCA1* | 9 | 107537851 | T/A | 0.03 | 0.06 | 0 | 3' |
| rs78492621 | *ABCA1* | 9 | 107538082 | A/G | 0.02 | 0.03 | 0 | 3' |
| rs78751332 | *ABCA1* | 9 | 107538677 | T/TC | 0.19 | 0.81 | 0.78 | 3' |
| rs56888257 | *ABCA1* | 9 | 107538913 | G/C | 0.18 | 0.21 | 0.0013 | 3' |
| rs75871264 | *ABCA1* | 9 | 107539308 | T/C | 0.04 | 0.03 | 0 | 3' |
| rs79900774 | *ABCA1* | 9 | 107539341 | T/C | 0.21 | 0.22 | 0.0013 | 3' |
| rs2482430 | *ABCA1* | 9 | 107539352 | T/C | 0.1 | 0.95 | 0.79 | 3' |
| rs144351111 | *ABCA1* | 9 | 107539450 | C/G | 0.04 | 0.02 | 0 | 3' |
| rs187954178 | *ABCA1* | 9 | 107539997 | A/G | 0.04 | 0.02 | 0 | 3' |
| rs34935948 | *ABCA1* | 9 | 107540095 | A/AT | 0.36 | 0.41 | 0.34 | 3' |
| rs2515605 | *ABCA1* | 9 | 107540189 | G/T | 0.1 | 0.95 | 0.79 | 3' |
| rs2482431 | *ABCA1* | 9 | 107540284 | C/T | 0.07 | 0.96 | 0.79 | 3' |
| rs73663521 | *ABCA1* | 9 | 107540366 | C/G | 0.12 | 0.19 | 0.08 | 3' |
| rs138639263 | *ABCA1* | 9 | 107540380 | T/TCC | 0.04 | 0.04 | 0.01 | 3' |
| rs10739906 | *ABCA1* | 9 | 107540484 | G/A | 0.33 | 0.74 | 0.35 | 3' |
| rs143998148 | *ABCA1* | 9 | 107540542 | G/A | 0.04 | 0.06 | 0 | 3' |
| rs146418615 | *ABCA1* | 9 | 107540731 | C/T | 0.03 | 0.03 | 0 | 3' |
| rs12338431 | *ABCA1* | 9 | 107540745 | T/G | 0.12 | 0.12 | 0.0013 | 3' |
| rs10820731 | *ABCA1* | 9 | 107540809 | A/G | 0.25 | 0.34 | 0.27 | 3' |
| rs12683154 | *ABCA1* | 9 | 107540983 | A/G | 0.13 | 0.19 | 0.08 | 3' |
| rs10124469 | *ABCA1* | 9 | 107541242 | C/T | 0.09 | 0.09 | 0.0013 | 3' |
| rs28408753 | *ABCA1* | 9 | 107541618 | C/G | 0.12 | 0.12 | 0.0013 | 3' |
| rs148641980 | *ABCA1* | 9 | 107541732 | AAG/A | 0.22 | 0.31 | 0.26 | 3' |
| rs201637768 | *ABCA1* | 9 | 107541899 | T/TG | 0.25 | 0.33 | 0.26 | 3' |
| rs10761085 | *ABCA1* | 9 | 107542068 | G/T | 0.2 | 0.27 | 0.26 | 3' |
| rs138217135 | *ABCA1* | 9 | 107542636 | G/C | 0.03 | 0.02 | 0 | 3' |
| rs76480790 | *ABCA1* | 9 | 107542807 | G/A | 0.06 | 0.13 | 0 | 3' |
| rs2482432 | *ABCA1* | 9 | 107543172 | T/C | 0.25 | 0.23 | 0.44 | 3' |
| rs148080589 | *ABCA1* | 9 | 107543345 | A/G | 0.01 | 0.01 | 0 | 3' |
| rs77877520 | *ABCA1* | 9 | 107543513 | A/G | 0.03 | 0.03 | 0 | 3' |
| rs199849057 | *ABCA1* | 9 | 107543691 | GATA/G | 0.11 | 0.18 | 0.07 | 3' |
| rs139196614 | *ABCA1* | 9 | 107543696 | TAAC/T | 0.11 | 0.18 | 0.07 | 3' |
| rs75141626 | *ABCA1* | 9 | 107543891 | C/T | 0.03 | 0.05 | 0 | 3' |
| 9.106583788 | *ABCA1* | 9 | 107543967 | G/T | 0.0008 | - | - | 3' |
| rs4149341 | *ABCA1* | 9 | 107544285 | T/C | 0.04 | 0.03 | 0.18 | 3' |
| rs4149340 | *ABCA1* | 9 | 107544685 | G/A | 0.08 | 0.1 | 0.0013 | 3' |
| rs363717 | *ABCA1* | 9 | 107544700 | C/T | 0.07 | 0.96 | 0.79 | 3' |
| rs41432545 | *ABCA1* | 9 | 107544943 | A/T | 0.18 | 0.21 | 0.0013 | 3' |
| rs4149339 | *ABCA1* | 9 | 107545156 | G/A | 0.34 | 0.42 | 0.26 | 3' |
| rs4149338 | *ABCA1* | 9 | 107545903 | G/A | 0.35 | 0.42 | 0.27 | 3' |
| rs73517870 | *ABCA1* | 9 | 107546201 | A/T | 0.16 | 0.2 | 0.0013 | 3' |
| rs74316246 | *ABCA1* | 9 | 107546500 | A/G | 0.02 | 0.03 | 0 | 3' |
| rs34879708 | *ABCA1* | 9 | 107546653 | G/T | 0.02 | 0.02 | 0 | NonSyn |
| rs2482433 | *ABCA1* | 9 | 107546901 | A/G | 0.05 | 0.04 | 0.28 | Intron |
| rs2472470 | *ABCA1* | 9 | 107547024 | G/A | 0.12 | 0.91 | 0.76 | Intron |
| rs112590861 | *ABCA1* | 9 | 107547047 | C/T | 0.02 | 0.01 | 0 | Intron |
| rs143224478 | *ABCA1* | 9 | 107547060 | G/A | 0.02 | 0.03 | 0 | Intron |
| rs111856857 | *ABCA1* | 9 | 107547081 | T/C | 0.03 | 0.04 | 0 | Intron |
| rs142026994 | *ABCA1* | 9 | 107547113 | C/T | 0.03 | 0.05 | 0 | Intron |
| rs10820732 | *ABCA1* | 9 | 107547215 | A/G | 0.12 | 0.15 | 0.23 | Intron |
| rs10820733 | *ABCA1* | 9 | 107547217 | G/A | 0.11 | 0.15 | 0.2 | Intron |
| rs11789818 | *ABCA1* | 9 | 107547230 | C/T | 0.27 | 0.25 | 0.22 | Intron |
| rs61741359 | *ABCA1* | 9 | 107547872 | C/T | 0.02 | 0.04 | 0.0013 | Syn |
| rs73517872 | *ABCA1* | 9 | 107548158 | G/T | 0.03 | 0.03 | 0 | Intron |
| rs62566031 | *ABCA1* | 9 | 107548250 | T/A | 0.04 | 0.04 | 0.11 | Intron |
| rs201153783 | *ABCA1* | 9 | 107548299 | A/AT | 0.02 | 0.03 | 0 | Intron |
| rs2777797 | *ABCA1* | 9 | 107548375 | C/A | 0.06 | 0.93 | 0.89 | Intron |
| rs2066882 | *ABCA1* | 9 | 107548462 | T/C | 0.15 | 0.14 | 0.06 | Intron |
| rs2740485 | *ABCA1* | 9 | 107548493 | A/C | 0.06 | 0.93 | 0.89 | Intron |
| rs2066881 | *ABCA1* | 9 | 107548566 | T/C | 0.02 | 0.01 | 0.04 | Intron |
| rs4149337 | *ABCA1* | 9 | 107548818 | A/G | 0.02 | 0.01 | 0.04 | Intron |
| rs116184208 | *ABCA1* | 9 | 107548886 | A/C | 0.02 | 0.03 | 0 | Intron |
| rs114821402 | *ABCA1* | 9 | 107549065 | G/A | 0.03 | 0.05 | 0 | Intron |
| rs73517878 | *ABCA1* | 9 | 107549291 | G/A | 0.11 | 0.09 | 0.01 | Intron |
| rs73517880 | *ABCA1* | 9 | 107549560 | A/G | 0.01 | 0.03 | 0 | Intron |
| rs2274871 | *ABCA1* | 9 | 107549593 | C/G | 0.02 | 0.01 | 0.04 | Intron |
| rs62566032 | *ABCA1* | 9 | 107549827 | G/A | 0.03 | 0.02 | 0.12 | Intron |
| rs9282537 | *ABCA1* | 9 | 107550222 | G/A | 0.19 | 0.23 | 0.01 | Syn |
| rs73517881 | *ABCA1* | 9 | 107550404 | A/G | 0.07 | 0.1 | 0 | Intron |
| rs116416613 | *ABCA1* | 9 | 107550632 | C/T | 0.02 | 0.03 | 0 | Intron |
| rs4149336 | *ABCA1* | 9 | 107550639 | A/G | 0.17 | 0.13 | 0.18 | Intron |
| rs146814933 | *ABCA1* | 9 | 107550645 | CA/C | 0.04 | 0.05 | 0 | Intron |
| rs41422749 | *ABCA1* | 9 | 107550683 | C/G | 0.04 | 0.05 | 0 | Intron |
| rs2740484 | *ABCA1* | 9 | 107551180 | C/T | 0.13 | 0.1 | 0.35 | Intron |
| rs2297405 | *ABCA1* | 9 | 107551247 | A/C | 0.02 | 0.02 | 0.05 | Intron |
| rs10116972 | *ABCA1* | 9 | 107551529 | C/T | 0.29 | 0.38 | 0.02 | Intron |
| rs2297406 | *ABCA1* | 9 | 107551538 | C/T | 0.06 | 0.03 | 0.31 | Intron |
| rs80300843 | *ABCA1* | 9 | 107551659 | C/T | 0.09 | 0.15 | 0 | Intron |
| rs4149335 | *ABCA1* | 9 | 107551726 | T/A | 0.36 | 0.46 | 0.08 | Intron |
| rs79848584 | *ABCA1* | 9 | 107551746 | C/T | 0.07 | 0.06 | 0 | Intron |
| rs113307134 | *ABCA1* | 9 | 107552052 | C/  CCACT | 0.37 | 0.46 | 0.08 | Intron |
| rs115963220 | *ABCA1* | 9 | 107552061 | T/C | 0.09 | 0.18 | 0.01 | Intron |
| rs116383884 | *ABCA1* | 9 | 107552097 | G/A | 0.02 | 0.02 | 0.0026 | Intron |
| rs150725758 | *ABCA1* | 9 | 107552170 | C/G | 0.11 | 0.15 | 0 | Intron |
| rs73663522 | *ABCA1* | 9 | 107552501 | A/G | 0.04 | 0.05 | 0 | Intron |
| rs12003698 | *ABCA1* | 9 | 107552620 | T/C | 0.16 | 0.21 | 0.02 | Intron |
| rs4149334 | *ABCA1* | 9 | 107552695 | G/A | 0.45 | 0.57 | 0.09 | Intron |
| rs4149333 | *ABCA1* | 9 | 107552924 | A/G | 0.43 | 0.57 | 0.09 | Intron |
| rs4149332 | *ABCA1* | 9 | 107552939 | A/G | 0.45 | 0.57 | 0.1 | Intron |
| rs75799425 | *ABCA1* | 9 | 107553041 | T/C | 0.1 | 0.09 | 0 | Intron |
| rs2020927 | *ABCA1* | 9 | 107553185 | A/G | 0.48 | 0.6 | 0.1 | Intron |
| rs4149331 | *ABCA1* | 9 | 107553478 | A/T | 0.48 | 0.6 | 0.1 | Intron |
| rs10121901 | *ABCA1* | 9 | 107553524 | A/G | 0.48 | 0.6 | 0.1 | Intron |
| rs10124686 | *ABCA1* | 9 | 107553621 | G/A | 0.04 | 0.02 | 0.01 | Intron |
| rs112654966 | *ABCA1* | 9 | 107553784 | G/GGCT  CACT | 0.48 | 0.6 | 0.09 | Intron |
| rs2066720 | *ABCA1* | 9 | 107554069 | C/T | 0.49 | 0.6 | 0.1 | Intron |
| rs34078184 | *ABCA1* | 9 | 107554274 | A/G | 0.1 | 0.1 | 0 | Syn |
| rs74712259 | *ABCA1* | 9 | 107554416 | G/A | 0.08 | 0.08 | 0 | Intron |
| rs146800165 | *ABCA1* | 9 | 107554439 | C/T | 0.02 | 0.02 | 0 | Intron |
| rs2297407 | *ABCA1* | 9 | 107554521 | T/C | 0.48 | 0.6 | 0.1 | Intron |
| rs2150867 | *ABCA1* | 9 | 107554534 | T/C | 0.36 | 0.29 | 0.79 | Intron |
| rs4149330 | *ABCA1* | 9 | 107554744 | A/G | 0.07 | 0.08 | 0.06 | Intron |
| rs4149329 | *ABCA1* | 9 | 107554745 | A/T | 0.49 | 0.6 | 0.1 | Intron |
| rs4149328 | *ABCA1* | 9 | 107554828 | T/G | 0.18 | 0.24 | 0.02 | Intron |
| rs114719973 | *ABCA1* | 9 | 107555077 | T/G | 0.002 | 0.01 | 0 | NonSyn |
| rs2297408 | *ABCA1* | 9 | 107555306 | G/A | 0.18 | 0.24 | 0.02 | Intron |
| rs78072322 | *ABCA1* | 9 | 107555606 | G/A | 0.1 | 0.13 | 0 | Intron |
| rs4149327 | *ABCA1* | 9 | 107555711 | C/G | 0.32 | 0.4 | 0.08 | Intron |
| rs4149326 | *ABCA1* | 9 | 107556349 | T/C | 0.32 | 0.45 | 0.03 | Intron |
| rs55803446 | *ABCA1* | 9 | 107556416 | G/A | 0.45 | 0.33 | 0.78 | Intron |
| rs2740481 | *ABCA1* | 9 | 107556417 | C/A | 0.45 | 0.33 | 0.78 | Intron |
| rs78268193 | *ABCA1* | 9 | 107556958 | T/G | 0.03 | 0.06 | 0 | Intron |
| rs76996768 | *ABCA1* | 9 | 107557177 | T/C | 0.07 | 0.07 | 0.06 | Intron |
| rs10115901 | *ABCA1* | 9 | 107557574 | G/A | 0.03 | 0.02 | 0.01 | Intron |
| rs60791648 | *ABCA1* | 9 | 107557816 | G/A | 0.08 | 0.14 | 0 | Intron |
| rs2777798 | *ABCA1* | 9 | 107557865 | A/G | 0.07 | 0.93 | 0.89 | Intron |
| rs73519807 | *ABCA1* | 9 | 107558249 | A/G | 0.08 | 0.14 | 0 | Intron |
| rs73519810 | *ABCA1* | 9 | 107558766 | A/G | 0.23 | 0.17 | 0.01 | Intron |
| rs73519813 | *ABCA1* | 9 | 107558937 | C/A | 0.19 | 0.13 | 0 | Intron |
| rs4149325 | *ABCA1* | 9 | 107558983 | C/A | 0.02 | 0.0041 | 0.06 | Intron |
| rs2777799 | *ABCA1* | 9 | 107559059 | T/C | 0.22 | 0.82 | 0.89 | Intron |
| rs74378049 | *ABCA1* | 9 | 107559306 | A/G | 0.06 | 0.07 | 0 | Intron |
| rs73519816 | *ABCA1* | 9 | 107559468 | T/C | 0.08 | 0.13 | 0 | Intron |
| rs4149324 | *ABCA1* | 9 | 107559479 | T/C | 0.02 | 0.0041 | 0.06 | Intron |
| rs4149349 | *ABCA1* | 9 | 107559855 | C/CG | 0.15 | 0.07 | 0.68 | Intron |
| rs141575869 | *ABCA1* | 9 | 107559857 | G/GA | 0.15 | 0.07 | 0.67 | Intron |
| rs73519818 | *ABCA1* | 9 | 107559895 | C/T | 0.18 | 0.13 | 0 | Intron |
| rs116570904 | *ABCA1* | 9 | 107560130 | T/A | 0.03 | 0.05 | 0 | Intron |
| rs4149323 | *ABCA1* | 9 | 107560291 | G/A | 0.04 | 0.04 | 0.06 | Intron |
| rs116060323 | *ABCA1* | 9 | 107560396 | G/C | 0.03 | 0.03 | 0 | Intron |
| rs61240896 | *ABCA1* | 9 | 107560424 | G/C | 0.08 | 0.12 | 0 | Intron |
| rs75866150 | *ABCA1* | 9 | 107560454 | T/C | 0.2 | 0.17 | 0.01 | Intron |
| rs113456348 | *ABCA1* | 9 | 107560513 | T/C | 0.02 | 0.01 | 0 | Intron |
| rs60690601 | *ABCA1* | 9 | 107560647 | A/G | 0.32 | 0.3 | 0.01 | Intron |
| rs28690796 | *ABCA1* | 9 | 107561452 | T/C | 0.03 | 0.02 | 0.01 | Intron |
| rs2740480 | *ABCA1* | 9 | 107562557 | G/A | 0.22 | 0.17 | 0.79 | Intron |
| rs2230808 | *ABCA1* | 9 | 107562804 | T/C | 0.21 | 0.17 | 0.79 | NonSyn |
| rs112161989 | *ABCA1* | 9 | 107562939 | T/A | 0.03 | 0.03 | 0 | Intron |
| rs58902766 | *ABCA1* | 9 | 107563227 | T/C | 0.02 | 0.04 | 0.06 | Intron |
| rs2777800 | *ABCA1* | 9 | 107563269 | T/C | 0.4 | 0.59 | 0.82 | Intron |
| rs115559561 | *ABCA1* | 9 | 107563330 | T/C | 0.03 | 0.02 | 0 | Intron |
| rs2740479 | *ABCA1* | 9 | 107563437 | G/A | 0.16 | 0.09 | 0.68 | Intron |
| rs60707048 | *ABCA1* | 9 | 107563593 | C/T | 0.04 | 0.04 | 0.06 | Intron |
| rs2245793 | *ABCA1* | 9 | 107563603 | T/C | 0.09 | 0.88 | 0.87 | Intron |
| rs141925740 | *ABCA1* | 9 | 107563673 | CTA/C | 0.14 | 0.14 | 0.02 | Intron |
| rs2740478 | *ABCA1* | 9 | 107563687 | G/A | 0.09 | 0.04 | 0.36 | Intron |
| rs12004453 | *ABCA1* | 9 | 107563752 | C/T | 0.23 | 0.26 | 0.01 | Intron |
| rs200716170 | *ABCA1* | 9 | 107563833 | G/GT | 0.06 | 0.06 | 0 | Intron |
| rs60065600 | *ABCA1* | 9 | 107563880 | C/T | 0.04 | 0.04 | 0.06 | Intron |
| rs60401607 | *ABCA1* | 9 | 107564014 | G/A | 0.04 | 0.04 | 0.06 | Intron |
| rs2740477 | *ABCA1* | 9 | 107564077 | G/A | 0.35 | 0.29 | 0.79 | Intron |
| rs2297404 | *ABCA1* | 9 | 107564466 | C/G | 0.02 | 0.04 | 0.06 | Intron |
| rs200776670 | *ABCA1* | 9 | 107564569 | TCA/T | 0.21 | 0.16 | 0.72 | Intron |
| rs3831227 | *ABCA1* | 9 | 107564570 | CAG/C | 0.22 | 0.17 | 0.78 | Intron |
| rs72275993 | *ABCA1* | 9 | 107564573 | AG/A | 0.21 | 0.15 | 0.72 | Intron |
| rs78101606 | *ABCA1* | 9 | 107564584 | G/A | 0.03 | 0.03 | 0.0013 | Intron |
| rs1999431 | *ABCA1* | 9 | 107564676 | T/C | 0.17 | 0.18 | 0.03 | Intron |
| rs914545 | *ABCA1* | 9 | 107564741 | T/G | 0.02 | 0.99 | 0.89 | Intron |
| rs914546 | *ABCA1* | 9 | 107564839 | C/T | 0.13 | 0.15 | 0.02 | Intron |
| rs1883023 | *ABCA1* | 9 | 107564846 | T/C | 0.22 | 0.17 | 0.78 | Intron |
| rs55683502 | *ABCA1* | 9 | 107564867 | T/C | 0.09 | 0.13 | 0 | Intron |
| rs4149321 | *ABCA1* | 9 | 107564939 | G/A | 0.04 | 0.05 | 0.06 | Intron |
| rs73663550 | *ABCA1* | 9 | 107565111 | G/A | 0.04 | 0.05 | 0 | Intron |
| rs111840495 | *ABCA1* | 9 | 107565515 | C/T | 0.03 | 0.03 | 0 | Intron |
| rs78689349 | *ABCA1* | 9 | 107565919 | G/A | 0.04 | 0.05 | 0.06 | Intron |
| rs3780538 | *ABCA1* | 9 | 107565993 | A/G | 0.04 | 0.05 | 0.06 | Intron |
| rs12351534 | *ABCA1* | 9 | 107566049 | C/T | 0.04 | 0.02 | 0.01 | Intron |
| rs112397456 | *ABCA1* | 9 | 107566111 | C/G | 0.08 | 0.13 | 0 | Intron |
| rs4149348 | *ABCA1* | 9 | 107566515 | C/CA | 0.04 | 0.05 | 0.07 | Intron |
| rs2777801 | *ABCA1* | 9 | 107566877 | C/A | 0.22 | 0.83 | 0.88 | Intron |
| rs57533788 | *ABCA1* | 9 | 107567416 | C/T | 0.08 | 0.13 | 0 | Intron |
| rs181469381 | *ABCA1* | 9 | 107567472 | C/T | 0.02 | 0.01 | 0 | Intron |
| rs56083958 | *ABCA1* | 9 | 107567474 | G/A | 0.08 | 0.13 | 0 | Intron |
| rs12340337 | *ABCA1* | 9 | 107567482 | A/G | 0.04 | 0.03 | 0.01 | Intron |
| rs58491344 | *ABCA1* | 9 | 107567517 | G/T | 0.08 | 0.13 | 0 | Intron |
| rs115169883 | *ABCA1* | 9 | 107567964 | T/C | 0.03 | 0.04 | 0 | Intron |
| rs112599817 | *ABCA1* | 9 | 107568111 | C/T | 0.11 | 0.15 | 0 | Intron |
| rs12344798 | *ABCA1* | 9 | 107568207 | G/A | 0.25 | 0.28 | 0.01 | Intron |
| rs41494750 | *ABCA1* | 9 | 107568546 | C/A | 0.06 | 0.05 | 0 | Syn |
| rs2066716 | *ABCA1* | 9 | 107568705 | C/T | 0.01 | 0.02 | 0.1 | Syn |
| rs41412244 | *ABCA1* | 9 | 107568802 | C/T | 0.04 | 0.05 | 0 | Intron |
| rs79617159 | *ABCA1* | 9 | 107568936 | T/G | 0.03 | 0.03 | 0.01 | Intron |
| rs58583337 | *ABCA1* | 9 | 107569309 | A/T | 0.04 | 0.05 | 0 | Intron |
| rs2777802 | *ABCA1* | 9 | 107569337 | T/C | 0.34 | 0.36 | 0.78 | Intron |
| rs7862756 | *ABCA1* | 9 | 107569367 | C/T | 0.06 | 0.04 | 0 | Intron |
| rs112335062 | *ABCA1* | 9 | 107569386 | G/A | 0.02 | 0.03 | 0 | Intron |
| rs7855185 | *ABCA1* | 9 | 107569774 | T/C | 0.06 | 0.04 | 0 | Intron |
| rs150763760 | *ABCA1* | 9 | 107570099 | A/T | 0.03 | 0.06 | 0 | Intron |
| rs192759510 | *ABCA1* | 9 | 107570103 | T/A | 0.16 | 0.17 | 0.04 | Intron |
| rs199734367 | *ABCA1* | 9 | 107570148 | CACACA  CACAT/C | 0.49 | 0.51 | 0.73 | Intron |
| rs149134723 | *ABCA1* | 9 | 107570150 | CACAC  ACAT/C | 0.39 | 0.64 | 0.75 | Intron |
| rs201728177 | *ABCA1* | 9 | 107570154 | CACA  T/C | 0.36 | 0.65 | 0.77 | Intron |
| rs199941615 | *ABCA1* | 9 | 107570192 | CTTA/C | 0.16 | 0.18 | 0.07 | Intron |
| rs142282792 | *ABCA1* | 9 | 107570195 | ATTG/A | 0.17 | 0.2 | 0.08 | Intron |
| rs61548263 | *ABCA1* | 9 | 107570241 | A/C | 0.25 | 0.28 | 0.01 | Intron |
| rs141185177 | *ABCA1* | 9 | 107570403 | A/AT | 0.08 | 0.13 | 0 | Intron |
| rs114992777 | *ABCA1* | 9 | 107570479 | G/A | 0.03 | 0.03 | 0 | Intron |
| rs115551229 | *ABCA1* | 9 | 107570547 | C/G | 0.03 | 0.03 | 0 | Intron |
| rs78933698 | *ABCA1* | 9 | 107570693 | C/T | 0.03 | 0.03 | 0 | Intron |
| rs2740476 | *ABCA1* | 9 | 107570714 | C/G | 0.5 | 0.45 | 0.17 | Intron |
| rs74441447 | *ABCA1* | 9 | 107570803 | G/A | 0.08 | 0.1 | 0 | Intron |
| rs2777803 | *ABCA1* | 9 | 107571208 | G/A | 0.02 | 1 | 0.89 | Intron |
| rs2777804 | *ABCA1* | 9 | 107571241 | C/T | 0.14 | 0.07 | 0.68 | Intron |
| rs2777805 | *ABCA1* | 9 | 107571375 | G/A | 0.49 | 0.53 | 0.79 | Intron |
| rs113132415 | *ABCA1* | 9 | 107572024 | G/A | 0.07 | 0.06 | 0.01 | Intron |
| rs2777806 | *ABCA1* | 9 | 107572119 | T/G | 0.02 | 0.0041 | 0.07 | Intron |
| rs7863889 | *ABCA1* | 9 | 107572134 | T/A | 0.1 | 0.08 | 0 | Intron |
| rs190236868 | *ABCA1* | 9 | 107572463 | A/T | 0.1 | 0.14 | 0.0026 | Intron |
| rs41354653 | *ABCA1* | 9 | 107573050 | C/T | 0.03 | 0.05 | 0 | Intron |
| rs115666991 | *ABCA1* | 9 | 107573239 | G/A | 0.03 | 0.03 | 0 | Intron |
| rs58695865 | *ABCA1* | 9 | 107573529 | T/C | 0.11 | 0.09 | 0 | Intron |
| rs4149319 | *ABCA1* | 9 | 107573757 | G/A | 0.09 | 0.13 | 0.02 | Intron |
| rs78634827 | *ABCA1* | 9 | 107573823 | T/C | 0.03 | 0.03 | 0 | Intron |
| rs114286452 | *ABCA1* | 9 | 107574510 | C/T | 0.03 | 0.03 | 0 | Intron |
| rs200915607 | *ABCA1* | 9 | 107574753 | GGC/G | 0.04 | 0.04 | 0 | Intron |
| rs201857140 | *ABCA1* | 9 | 107574757 | TCC/T | 0.04 | 0.04 | 0 | Intron |
| rs35545593 | *ABCA1* | 9 | 107574960 | C/T | 0.07 | 0.1 | 0 | Syn |
| rs73519840 | *ABCA1* | 9 | 107575281 | G/T | 0.18 | 0.23 | 0 | Intron |
| rs2297409 | *ABCA1* | 9 | 107576246 | G/A | 0.27 | 0.27 | 0.16 | Intron |
| rs41410048 | *ABCA1* | 9 | 107576648 | A/G | 0.02 | 0.04 | 0 | Intron |
| rs75488496 | *ABCA1* | 9 | 107576656 | A/G | 0.07 | 0.04 | 0 | Intron |
| rs111337110 | *ABCA1* | 9 | 107576665 | A/G | 0.02 | 0.05 | 0.01 | Intron |
| rs9282540 | *ABCA1* | 9 | 107576822 | G/A | 0.19 | 0.25 | 0 | Intron |
| rs58694166 | *ABCA1* | 9 | 107577200 | T/C | 0.03 | 0.03 | 0.01 | Intron |
| rs147236645 | *ABCA1* | 9 | 107577218 | G/A | 0.08 | 0.1 | 0 | Intron |
| rs140853355 | *ABCA1* | 9 | 107577221 | A/G | 0.08 | 0.1 | 0 | Intron |
| rs61349465 | *ABCA1* | 9 | 107577299 | A/T | 0.09 | 0.08 | 0 | Intron |
| rs73519841 | *ABCA1* | 9 | 107577534 | G/T | 0.17 | 0.22 | 0 | Intron |
| rs149792445 | *ABCA1* | 9 | 107577575 | A/T | 0.02 | 0.03 | 0 | Intron |
| rs76512079 | *ABCA1* | 9 | 107577986 | G/A | 0.02 | 0.03 | 0 | Intron |
| rs3780540 | *ABCA1* | 9 | 107578048 | C/G | 0.11 | 0.12 | 0.02 | Intron |
| rs71511786 | *ABCA1* | 9 | 107578059 | A/T | 0.02 | 0.01 | 0.07 | Intron |
| rs3780541 | *ABCA1* | 9 | 107578103 | A/C | 0.02 | 0.0041 | 0.06 | Intron |
| rs41513148 | *ABCA1* | 9 | 107578301 | T/C | 0.07 | 0.11 | 0 | Intron |
| rs2234885 | *ABCA1* | 9 | 107578401 | G/A | 0.12 | 0.13 | 0.01 | Intron |
| rs2230807 | *ABCA1* | 9 | 107578478 | C/T | 0.18 | 0.23 | 0 | Syn |
| rs34788556 | *ABCA1* | 9 | 107578529 | T/C | 0.12 | 0.11 | 0.01 | Syn |
| rs112338016 | *ABCA1* | 9 | 107578676 | G/A | 0.02 | 0.02 | 0 | Intron |
| rs78103745 | *ABCA1* | 9 | 107578749 | T/G | 0.05 | 0.06 | 0.0026 | Intron |
| rs73663553 | *ABCA1* | 9 | 107578888 | A/G | 0.04 | 0.05 | 0 | Intron |
| rs111975414 | *ABCA1* | 9 | 107579252 | C/G | 0.17 | 0.16 | 0.01 | Intron |
| rs41456650 | *ABCA1* | 9 | 107579464 | G/A | 0.13 | 0.14 | 0.0013 | Intron |
| rs2297401 | *ABCA1* | 9 | 107579472 | A/C | 0.03 | 0.02 | 0.04 | Intron |
| rs33918808 | *ABCA1* | 9 | 107579632 | C/G | 0.18 | 0.16 | 0.03 | NonSyn |
| rs28652716 | *ABCA1* | 9 | 107579818 | T/C | 0.04 | 0.03 | 0.01 | Intron |
| rs2297402 | *ABCA1* | 9 | 107579880 | C/T | 0.02 | 0.05 | 0.02 | Intron |
| rs73663554 | *ABCA1* | 9 | 107580203 | T/C | 0.04 | 0.05 | 0 | Intron |
| rs9299383 | *ABCA1* | 9 | 107580210 | G/C | 0.07 | 0.07 | 0.01 | Intron |
| rs2472371 | *ABCA1* | 9 | 107580427 | A/G | 0.05 | 0.06 | 0.01 | Intron |
| rs58866705 | *ABCA1* | 9 | 107580480 | C/T | 0.05 | 0.08 | 0.01 | Intron |
| rs2020926 | *ABCA1* | 9 | 107580842 | A/G | 0.02 | 0.01 | 0.05 | Intron |
| rs35204915 | *ABCA1* | 9 | 107581042 | G/A | 0.12 | 0.18 | 0 | Syn |
| rs4149316 | *ABCA1* | 9 | 107581307 | C/T | 0.02 | 0.01 | 0.06 | Intron |
| rs77368189 | *ABCA1* | 9 | 107581423 | G/A | 0.07 | 0.11 | 0.0013 | Intron |
| rs114817285 | *ABCA1* | 9 | 107581536 | C/T | 0.05 | 0.08 | 0 | Intron |
| rs2254884 | *ABCA1* | 9 | 107581749 | A/C | 0.09 | 0.09 | 0.26 | Intron |
| rs78329992 | *ABCA1* | 9 | 107582360 | G/A | 0.01 | 0.03 | 0 | Intron |
| rs3780542 | *ABCA1* | 9 | 107582780 | G/A | 0.08 | 0.11 | 0.06 | Intron |
| rs115139126 | *ABCA1* | 9 | 107582787 | T/G | 0.04 | 0.04 | 0.01 | Intron |
| rs55993392 | *ABCA1* | 9 | 107582998 | C/G | 0.02 | 0.06 | 0.09 | Intron |
| rs60913410 | *ABCA1* | 9 | 107583638 | C/T | 0.05 | 0.1 | 0 | Intron |
| rs35561837 | *ABCA1* | 9 | 107583748 | G/A | 0.02 | 0.05 | 0 | Syn |
| rs139397024 | *ABCA1* | 9 | 107583915 | C/T | 0.02 | 0.01 | 0 | Intron |
| rs73519850 | *ABCA1* | 9 | 107583954 | G/A | 0.02 | 0.02 | 0 | Intron |
| rs4149315 | *ABCA1* | 9 | 107584309 | T/C | 0.02 | 0.02 | 0.05 | Intron |
| rs60274038 | *ABCA1* | 9 | 107584319 | G/A | 0.05 | 0.07 | 0 | Intron |
| rs4149314 | *ABCA1* | 9 | 107584351 | T/C | 0.02 | 0.07 | 0.1 | Intron |
| rs116092725 | *ABCA1* | 9 | 107584442 | G/A | 0.05 | 0.09 | 0 | Intron |
| rs73663556 | *ABCA1* | 9 | 107584497 | C/A | 0.03 | 0.04 | 0 | Intron |
| rs9282546 | *ABCA1* | 9 | 107584791 | C/T | 0.01 | 0.02 | 0 | Syn |
| rs41445345 | *ABCA1* | 9 | 107585000 | G/C | 0.04 | 0.07 | 0 | Intron |
| rs3818689 | *ABCA1* | 9 | 107585016 | G/C | 0.03 | 0.02 | 0.06 | Intron |
| rs112853430 | *ABCA1* | 9 | 107585213 | C/T | 0.05 | 0.11 | 0.03 | Intron |
| rs7025776 | *ABCA1* | 9 | 107585316 | T/G | 0.3 | 0.32 | 0.06 | Intron |
| rs184186942 | *ABCA1* | 9 | 107585602 | C/T | 0.01 | 0.02 | 0 | Intron |
| rs145060803 | *ABCA1* | 9 | 107585727 | G/A | 0.01 | 0.04 | 0.0013 | Intron |
| rs75003539 | *ABCA1* | 9 | 107585841 | G/A | 0.02 | 0.02 | 0.05 | Intron |
| rs35093463 | *ABCA1* | 9 | 107586238 | C/A | 0.03 | 0.02 | 0.06 | Intron |
| rs137994801 | *ABCA1* | 9 | 107586520 | CAAA  G/C | 0.02 | 0.02 | 0.05 | Intron |
| rs9282544 | *ABCA1* | 9 | 107586686 | A/G | 0.03 | 0.03 | 0 | Intron |
| rs2066714 | *ABCA1* | 9 | 107586753 | T/C | 0.48 | 0.5 | 0.12 | NonSyn |
| rs35207495 | *ABCA1* | 9 | 107586800 | C/T | 0.01 | 0.01 | 0 | NonSyn |
| rs139457469 | *ABCA1* | 9 | 107586862 | G/A | 0.01 | 0.01 | 0 | Intron |
| rs61648534 | *ABCA1* | 9 | 107586896 | T/A | 0.01 | 0.04 | 0 | Intron |
| rs74471062 | *ABCA1* | 9 | 107587171 | C/T | 0.05 | 0.08 | 0 | Intron |
| rs115169715 | *ABCA1* | 9 | 107587766 | T/A | 0.03 | 0.03 | 0 | Intron |
| rs77755970 | *ABCA1* | 9 | 107587780 | T/A | 0.02 | 0.05 | 0 | Intron |
| rs145248363 | *ABCA1* | 9 | 107587835 | A/C | 0.02 | 0.01 | 0 | Intron |
| rs2066715 | *ABCA1* | 9 | 107588033 | C/T | 0.02 | 0.0041 | 0.06 | NonSyn |
| rs78103171 | *ABCA1* | 9 | 107588262 | T/C | 0.03 | 0.03 | 0 | Intron |
| rs3824477 | *ABCA1* | 9 | 107588328 | G/A | 0.05 | 0.11 | 0.02 | Intron |
| rs3780543 | *ABCA1* | 9 | 107588572 | A/G | 0.43 | 0.47 | 0.11 | Intron |
| rs74821460 | *ABCA1* | 9 | 107588613 | T/G | 0.02 | 0.05 | 0 | Intron |
| rs4149311 | *ABCA1* | 9 | 107588777 | C/T | 0.43 | 0.47 | 0.11 | Intron |
| rs77796588 | *ABCA1* | 9 | 107588993 | C/T | 0.05 | 0.07 | 0 | Intron |
| rs57753200 | *ABCA1* | 9 | 107589074 | G/T | 0.15 | 0.21 | 0 | Intron |
| rs4149310 | *ABCA1* | 9 | 107589134 | A/T | 0.32 | 0.74 | 0.14 | Intron |
| rs2066718 | *ABCA1* | 9 | 107589255 | C/T | 0.09 | 0.12 | 0.02 | NonSyn |
| rs7024300 | *ABCA1* | 9 | 107589567 | C/T | 0.05 | 0.11 | 0.02 | Intron |
| rs4149309 | *ABCA1* | 9 | 107589580 | A/T | 0.43 | 0.47 | 0.11 | Intron |
| rs4149308 | *ABCA1* | 9 | 107589630 | C/T | 0.41 | 0.47 | 0.11 | Intron |
| rs73519870 | *ABCA1* | 9 | 107589710 | C/T | 0.14 | 0.16 | 0 | Intron |
| rs4149307 | *ABCA1* | 9 | 107589744 | C/T | 0.32 | 0.74 | 0.14 | Intron |
| rs10991383 | *ABCA1* | 9 | 107589795 | C/G | 0.02 | 0.02 | 0.05 | Intron |
| rs4149306 | *ABCA1* | 9 | 107589815 | C/T | 0.04 | 0.03 | 0.01 | Intron |
| rs10820736 | *ABCA1* | 9 | 107589947 | G/C | 0.02 | 0.01 | 0.05 | Intron |
| rs78794757 | *ABCA1* | 9 | 107590446 | C/T | 0.02 | 0.02 | 0.05 | Intron |
| rs73519874 | *ABCA1* | 9 | 107590627 | C/T | 0.17 | 0.19 | 0 | Intron |
| rs148364457 | *ABCA1* | 9 | 107590658 | G/C | 0.02 | 0.01 | 0 | Intron |
| rs73663557 | *ABCA1* | 9 | 107590769 | C/T | 0.03 | 0.04 | 0 | Intron |
| rs73663558 | *ABCA1* | 9 | 107590957 | G/A | 0.06 | 0.1 | 0 | Intron |
| rs13306069 | *ABCA1* | 9 | 107591024 | C/T | 0.03 | 0.02 | 0.06 | Intron |
| rs10820737 | *ABCA1* | 9 | 107591082 | G/T | 0.03 | 0.03 | 0.05 | Intron |
| rs10820738 | *ABCA1* | 9 | 107591116 | T/C | 0.03 | 0.03 | 0.05 | Intron |
| rs2853579 | *ABCA1* | 9 | 107591272 | G/T | 0.42 | 0.46 | 0.11 | Syn |
| rs2066717 | *ABCA1* | 9 | 107591478 | G/A | 0.01 | 0.02 | 0.06 | Intron |
| rs114427540 | *ABCA1* | 9 | 107591623 | T/C | 0.03 | 0.03 | 0 | Intron |
| rs116572835 | *ABCA1* | 9 | 107591779 | A/G | 0.02 | 0.01 | 0 | Intron |
| rs77120011 | *ABCA1* | 9 | 107591807 | C/T | 0.02 | 0.02 | 0.05 | Intron |
| rs80246211 | *ABCA1* | 9 | 107591908 | A/C | 0.09 | 0.13 | 0 | Intron |
| rs4743762 | *ABCA1* | 9 | 107592189 | C/A | 0.43 | 0.47 | 0.11 | Intron |
| rs77477349 | *ABCA1* | 9 | 107592358 | G/A | 0.05 | 0.08 | 0 | Intron |
| rs73663560 | *ABCA1* | 9 | 107592365 | G/A | 0.03 | 0.04 | 0 | Intron |
| rs72607108 | *ABCA1* | 9 | 107592641 | T/G | 0.02 | 0.06 | 0.1 | Intron |
| rs58725938 | *ABCA1* | 9 | 107593002 | T/C | 0.05 | 0.08 | 0 | Intron |
| rs4743763 | *ABCA1* | 9 | 107593182 | T/A | 0.24 | 0.85 | 0.25 | Intron |
| rs41504247 | *ABCA1* | 9 | 107593411 | T/C | 0.01 | 0.02 | 0 | Intron |
| rs150237889 | *ABCA1* | 9 | 107593620 | T/C | 0.02 | 0.07 | 0.1 | Intron |
| rs139365336 | *ABCA1* | 9 | 107593624 | G/A | 0.02 | 0.01 | 0 | Intron |
| rs112953925 | *ABCA1* | 9 | 107593687 | T/C | 0.1 | 0.14 | 0 | Intron |
| rs9282539 | *ABCA1* | 9 | 107593983 | G/A | 0.02 | 0.02 | 0 | Syn |
| rs116581454 | *ABCA1* | 9 | 107594286 | T/C | 0.05 | 0.08 | 0 | Intron |
| rs2515629 | *ABCA1* | 9 | 107594364 | A/G | 0.14 | 0.18 | 0.17 | Intron |
| rs4149304 | *ABCA1* | 9 | 107594474 | T/C | 0.02 | 0.02 | 0.05 | Intron |
| rs4149303 | *ABCA1* | 9 | 107594515 | A/G | 0.28 | 0.3 | 0.11 | Intron |
| rs77437185 | *ABCA1* | 9 | 107594698 | G/A | 0.04 | 0.1 | 0.03 | Intron |
| rs55659052 | *ABCA1* | 9 | 107594770 | CT/C | 0.02 | 0.07 | 0.1 | Intron |
| rs13306066 | *ABCA1* | 9 | 107595210 | G/A | 0.04 | 0.1 | 0.03 | Intron |
| rs7873387 | *ABCA1* | 9 | 107595602 | A/C | 0.28 | 0.3 | 0.11 | Intron |
| rs79153526 | *ABCA1* | 9 | 107595684 | A/G | 0.03 | 0.02 | 0.05 | Intron |
| rs115351407 | *ABCA1* | 9 | 107595725 | A/G | 0.03 | 0.03 | 0 | Intron |
| rs12351480 | *ABCA1* | 9 | 107595978 | C/A | 0.32 | 0.34 | 0.12 | Intron |
| rs73663561 | *ABCA1* | 9 | 107596087 | G/A | 0.03 | 0.03 | 0 | Intron |
| rs7019569 | *ABCA1* | 9 | 107596101 | T/C | 0.26 | 0.28 | 0.06 | Intron |
| rs4149302 | *ABCA1* | 9 | 107596205 | G/A | 0.27 | 0.29 | 0.06 | Intron |
| rs4149301 | *ABCA1* | 9 | 107596285 | T/C | 0.31 | 0.32 | 0.06 | Intron |
| rs4149300 | *ABCA1* | 9 | 107596411 | C/G | 0.29 | 0.35 | 0.13 | Intron |
| rs79983614 | *ABCA1* | 9 | 107596590 | C/T | 0.03 | 0.04 | 0 | Intron |
| rs2472390 | *ABCA1* | 9 | 107596602 | T/C | 0.23 | 0.3 | 0.1 | Intron |
| rs77776122 | *ABCA1* | 9 | 107596728 | C/T | 0.03 | 0.03 | 0.01 | Intron |
| rs2472389 | *ABCA1* | 9 | 107596743 | T/C | 0.2 | 0.24 | 0.1 | Intron |
| rs146189769 | *ABCA1* | 9 | 107596846 | AC/A | 0.04 | 0.05 | 0.01 | Intron |
| rs7034361 | *ABCA1* | 9 | 107596854 | G/A | 0.26 | 0.27 | 0.06 | Intron |
| rs115040046 | *ABCA1* | 9 | 107596931 | G/C | 0.04 | 0.03 | 0.01 | Intron |
| rs57993463 | *ABCA1* | 9 | 107596995 | G/A | 0.05 | 0.06 | 0 | Intron |
| rs114312617 | *ABCA1* | 9 | 107597252 | G/A | 0.05 | 0.08 | 0 | Intron |
| rs2472388 | *ABCA1* | 9 | 107597504 | G/A | 0.19 | 0.24 | 0.1 | Intron |
| rs59470590 | *ABCA1* | 9 | 107597592 | T/C | 0.05 | 0.08 | 0 | Intron |
| rs2482418 | *ABCA1* | 9 | 107597722 | A/G | 0.23 | 0.83 | 0.31 | Intron |
| rs79325047 | *ABCA1* | 9 | 107598399 | A/T | 0.04 | 0.03 | 0.01 | Intron |
| rs73663562 | *ABCA1* | 9 | 107598439 | C/T | 0.03 | 0.03 | 0 | Intron |
| rs115905886 | *ABCA1* | 9 | 107598619 | G/A | 0.02 | 0.03 | 0.01 | NonSyn |
| rs2065412 | *ABCA1* | 9 | 107598740 | T/C | 0.09 | 0.96 | 0.57 | Intron |
| rs60070164 | *ABCA1* | 9 | 107598843 | G/C | 0.04 | 0.07 | 0 | Intron |
| rs2297398 | *ABCA1* | 9 | 107598884 | A/C | 0.04 | 0.03 | 0.05 | Intron |
| rs2297399 | *ABCA1* | 9 | 107599390 | G/A | 0.02 | 0.0041 | 0.06 | Intron |
| rs73663563 | *ABCA1* | 9 | 107599405 | G/A | 0.03 | 0.03 | 0 | Intron |
| rs5899622 | *ABCA1* | 9 | 107599412 | AAGG/A | 0.19 | 0.24 | 0.1 | Intron |
| rs72548382 | *ABCA1* | 9 | 107599438 | G/GC | 0.34 | - | - | Intron |
| rs41391445 | *ABCA1* | 9 | 107599937 | T/C | 0.09 | 0.13 | 0 | Intron |
| rs115176936 | *ABCA1* | 9 | 107600128 | C/T | 0.03 | 0.03 | 0 | Intron |
| rs73663564 | *ABCA1* | 9 | 107600164 | T/C | 0.03 | 0.03 | 0 | Intron |
| rs2515602 | *ABCA1* | 9 | 107600168 | G/A | 0.27 | 0.22 | 0.31 | Intron |
| rs2515601 | *ABCA1* | 9 | 107600193 | A/G | 0.19 | 0.24 | 0.1 | Intron |
| rs4149299 | *ABCA1* | 9 | 107600251 | T/C | 0.06 | 0.11 | 0.1 | Intron |
| rs35545100 | *ABCA1* | 9 | 107600404 | C/G | 0.02 | 0.0041 | 0.06 | Intron |
| rs2989944 | *ABCA1* | 9 | 107600579 | T/C | 0.36 | 0.69 | 0.3 | Intron |
| rs2472387 | *ABCA1* | 9 | 107600815 | G/A | 0.22 | 0.24 | 0.1 | Intron |
| rs7020957 | *ABCA1* | 9 | 107600881 | G/A | 0.03 | 0.04 | 0 | Intron |
| rs115243263 | *ABCA1* | 9 | 107601222 | C/A | 0.02 | 0.03 | 0 | Intron |
| rs111516175 | *ABCA1* | 9 | 107601418 | T/C | 0.02 | 0.03 | 0 | Intron |
| rs73519894 | *ABCA1* | 9 | 107601436 | G/T | 0.02 | 0.03 | 0 | Intron |
| rs2472386 | *ABCA1* | 9 | 107601541 | G/A | 0.27 | 0.77 | 0.31 | Intron |
| rs12235875 | *ABCA1* | 9 | 107601625 | T/C | 0.04 | 0.03 | 0.05 | Intron |
| rs12342847 | *ABCA1* | 9 | 107601703 | G/A | 0.04 | 0.02 | 0.01 | Intron |
| rs199703683 | *ABCA1* | 9 | 107601800 | TC/T | 0.06 | 0.13 | 0.09 | Intron |
| rs200096286 | *ABCA1* | 9 | 107601933 | AC/A | 0.03 | 0.03 | 0.01 | Intron |
| rs55874167 | *ABCA1* | 9 | 107602342 | T/G | 0.03 | 0.08 | 0.09 | Intron |
| rs2246841 | *ABCA1* | 9 | 107602666 | C/T | 0.2 | 0.25 | 0.1 | Syn |
| rs2274873 | *ABCA1* | 9 | 107602678 | G/A | 0.06 | 0.11 | 0.09 | Syn |
| rs4149298 | *ABCA1* | 9 | 107603043 | G/A | 0.06 | 0.11 | 0.1 | Intron |
| rs2472450 | *ABCA1* | 9 | 107603174 | C/T | 0.2 | 0.25 | 0.1 | Intron |
| rs143072975 | *ABCA1* | 9 | 107603206 | TAA/T | 0.2 | 0.25 | 0.1 | Intron |
| rs2472385 | *ABCA1* | 9 | 107603278 | T/C | 0.06 | 0.12 | 0.1 | Intron |
| rs2515600 | *ABCA1* | 9 | 107603284 | A/G | 0.2 | 0.25 | 0.1 | Intron |
| rs73519901 | *ABCA1* | 9 | 107603304 | G/A | 0.04 | 0.08 | 0.01 | Intron |
| rs2487062 | *ABCA1* | 9 | 107603439 | G/C | 0.19 | 0.25 | 0.1 | Intron |
| rs12335579 | *ABCA1* | 9 | 107603454 | C/T | 0.03 | 0.02 | 0.01 | Intron |
| rs115106108 | *ABCA1* | 9 | 107603569 | A/G | 0.03 | 0.03 | 0 | Intron |
| rs36098089 | *ABCA1* | 9 | 107603924 | GC/G | 0.14 | 0.17 | 0.05 | Intron |
| rs2487065 | *ABCA1* | 9 | 107603963 | G/C | 0.11 | 0.18 | 0.1 | Intron |
| rs4149297 | *ABCA1* | 9 | 107604104 | A/G | 0.06 | 0.11 | 0.1 | Intron |
| rs2472449 | *ABCA1* | 9 | 107604197 | T/G | 0.24 | 0.16 | 0.69 | Intron |
| rs3824478 | *ABCA1* | 9 | 107604385 | C/T | 0.02 | 0.02 | 0.05 | Intron |
| rs3824479 | *ABCA1* | 9 | 107604394 | T/A | 0.02 | 0.0041 | 0.05 | Intron |
| rs78733415 | *ABCA1* | 9 | 107604485 | C/A | 0.01 | 0.03 | 0 | Intron |
| rs3780546 | *ABCA1* | 9 | 107604582 | G/T | 0.04 | 0.03 | 0.05 | Intron |
| rs58219156 | *ABCA1* | 9 | 107604722 | C/A | 0.19 | 0.13 | 0.63 | Intron |
| rs114893478 | *ABCA1* | 9 | 107604842 | C/T | 0.02 | 0.01 | 0 | Intron |
| rs56274651 | *ABCA1* | 9 | 107604918 | T/C | 0.05 | 0.11 | 0.06 | Intron |
| rs73663566 | *ABCA1* | 9 | 107605112 | C/T | 0.04 | 0.07 | 0 | Intron |
| rs28599759 | *ABCA1* | 9 | 107605344 | C/T | 0.15 | 0.19 | 0.16 | Intron |
| rs73521808 | *ABCA1* | 9 | 107605521 | C/G | 0.05 | 0.1 | 0.0026 | Intron |
| rs78294949 | *ABCA1* | 9 | 107605638 | G/T | 0.05 | 0.1 | 0 | Intron |
| rs114851717 | *ABCA1* | 9 | 107606009 | G/C | 0.06 | 0.11 | 0 | Intron |
| rs34390877 | *ABCA1* | 9 | 107606044 | C/T | 0.03 | 0.07 | 0.09 | Intron |
| rs138618449 | *ABCA1* | 9 | 107606074 | ATCT/A | 0.06 | 0.11 | 0 | Intron |
| rs144258418 | *ABCA1* | 9 | 107606228 | C/T | 0.02 | 0.01 | 0 | Intron |
| rs148714575 | *ABCA1* | 9 | 107606383 | C/T | 0.06 | 0.11 | 0 | Intron |
| rs115761095 | *ABCA1* | 9 | 107606447 | G/C | 0.06 | 0.11 | 0 | Intron |
| rs76729624 | *ABCA1* | 9 | 107606514 | G/A | 0.06 | 0.11 | 0 | Intron |
| rs147489607 | *ABCA1* | 9 | 107606582 | C/A | 0.02 | 0.03 | 0 | Intron |
| rs139941675 | *ABCA1* | 9 | 107606583 | C/G | 0.03 | 0.03 | 0 | Intron |
| rs4149295 | *ABCA1* | 9 | 107607061 | T/A | 0.38 | 0.71 | 0.11 | Intron |
| rs143471798 | *ABCA1* | 9 | 107607105 | CAA/C | 0.07 | 0.11 | 0 | Intron |
| rs914544 | *ABCA1* | 9 | 107607212 | T/C | 0.04 | 0.03 | 0.03 | Intron |
| rs59862631 | *ABCA1* | 9 | 107607226 | T/C | 0.06 | 0.1 | 0 | Intron |
| rs41483745 | *ABCA1* | 9 | 107607420 | C/G | 0.43 | 0.49 | 0.004 | Intron |
| rs116728780 | *ABCA1* | 9 | 107607741 | G/A | 0.03 | 0.08 | 0 | Intron |
| rs80324148 | *ABCA1* | 9 | 107608144 | A/G | 0.03 | 0.06 | 0 | Intron |
| rs77257670 | *ABCA1* | 9 | 107608319 | T/C | 0.03 | 0.08 | 0 | Intron |
| rs114512051 | *ABCA1* | 9 | 107608654 | G/A | 0.03 | 0.08 | 0 | Intron |
| rs79227686 | *ABCA1* | 9 | 107608845 | T/C | 0.03 | 0.08 | 0 | Intron |
| rs4149292 | *ABCA1* | 9 | 107609065 | G/A | 0.03 | 0.07 | 0.09 | Intron |
| rs139257838 | *ABCA1* | 9 | 107609089 | CAGA  T/C | 0.02 | 0.07 | 0 | Intron |
| rs76258722 | *ABCA1* | 9 | 107609444 | G/A | 0.01 | 0.04 | 0 | Intron |
| rs4149291 | *ABCA1* | 9 | 107609476 | C/G | 0.34 | 0.75 | 0.11 | Intron |
| rs116606062 | *ABCA1* | 9 | 107610009 | C/T | 0.05 | 0.05 | 0.0026 | Intron |
| rs113337137 | *ABCA1* | 9 | 107610152 | T/C | 0.05 | 0.05 | 0.0026 | Intron |
| rs147453422 | *ABCA1* | 9 | 107610647 | A/C | 0.05 | 0.05 | 0.0026 | Intron |
| rs114548000 | *ABCA1* | 9 | 107610890 | G/A | 0.03 | 0.08 | 0 | Intron |
| rs76314320 | *ABCA1* | 9 | 107611132 | A/G | 0.16 | 0.2 | 0 | Intron |
| rs144360419 | *ABCA1* | 9 | 107611279 | C/T | 0.03 | 0.08 | 0.0013 | Intron |
| rs115763221 | *ABCA1* | 9 | 107611337 | G/T | 0.1 | 0.17 | 0.0026 | Intron |
| rs4149290 | *ABCA1* | 9 | 107611827 | T/C | 0.03 | 0.07 | 0.09 | Intron |
| rs4149289 | *ABCA1* | 9 | 107611830 | T/A | 0.11 | 0.18 | 0.09 | Intron |
| rs146415421 | *ABCA1* | 9 | 107612459 | G/C | 0.01 | 0.05 | 0.0013 | Intron |
| rs140025957 | *ABCA1* | 9 | 107612785 | TAAA  AG/T | 0.03 | 0.07 | 0.09 | Intron |
| rs60326642 | *ABCA1* | 9 | 107612889 | T/A | 0.02 | 0.07 | 0 | Intron |
| rs115231467 | *ABCA1* | 9 | 107612922 | A/G | 0.05 | 0.06 | 0.0026 | Intron |
| rs115883100 | *ABCA1* | 9 | 107613082 | C/G | 0.05 | 0.09 | 0.0026 | Intron |
| rs114404472 | *ABCA1* | 9 | 107613115 | C/T | 0.07 | 0.1 | 0.0026 | Intron |
| rs76153796 | *ABCA1* | 9 | 107613364 | C/A | 0.07 | 0.1 | 0.0026 | Intron |
| rs73521824 | *ABCA1* | 9 | 107613602 | A/G | 0.02 | 0.07 | 0 | Intron |
| rs115467372 | *ABCA1* | 9 | 107613678 | G/A | 0.05 | 0.06 | 0.0026 | Intron |
| rs73521828 | *ABCA1* | 9 | 107613849 | T/A | 0.08 | 0.18 | 0 | Intron |
| rs73663570 | *ABCA1* | 9 | 107614114 | T/A | 0.02 | 0.04 | 0 | Intron |
| rs113483939 | *ABCA1* | 9 | 107614132 | C/T | 0.06 | 0.12 | 0.01 | Intron |
| rs55864734 | *ABCA1* | 9 | 107615552 | C/A | 0.02 | 0.04 | 0 | Intron |
| rs200523782 | *ABCA1* | 9 | 107615653 | CCA/C | 0.02 | 0.06 | 0 | Intron |
| rs1340352 | *ABCA1* | 9 | 107615839 | C/T | 0.01 | 0.02 | 0 | Intron |
| rs1340353 | *ABCA1* | 9 | 107615852 | G/A | 0.06 | 0.08 | 0.0026 | Intron |
| rs112813864 | *ABCA1* | 9 | 107615915 | A/AT | 0.04 | 0.09 | 0 | Intron |
| rs73521831 | *ABCA1* | 9 | 107615958 | C/G | 0.04 | 0.09 | 0 | Intron |
| rs1340354 | *ABCA1* | 9 | 107616084 | G/A | 0.07 | 0.11 | 0.0026 | Intron |
| rs73521832 | *ABCA1* | 9 | 107616267 | G/C | 0.04 | 0.09 | 0 | Intron |
| rs73663571 | *ABCA1* | 9 | 107616516 | C/A | 0.02 | 0.04 | 0 | Intron |
| rs73663572 | *ABCA1* | 9 | 107616759 | G/A | 0.02 | 0.04 | 0 | Intron |
| rs75671121 | *ABCA1* | 9 | 107616778 | G/A | 0.02 | 0.03 | 0 | Intron |
| rs114432666 | *ABCA1* | 9 | 107617281 | G/C | 0.07 | 0.08 | 0.0026 | Intron |
| rs2487037 | *ABCA1* | 9 | 107617337 | C/T | 0.05 | 0.07 | 0.13 | Intron |
| rs2487039 | *ABCA1* | 9 | 107617433 | C/T | 0.05 | 0.07 | 0.13 | Intron |
| rs57634286 | *ABCA1* | 9 | 107617434 | G/A | 0.29 | 0.36 | 0 | Intron |
| rs140180943 | *ABCA1* | 9 | 107618006 | C/T | 0.02 | 0.03 | 0 | Intron |
| rs144999512 | *ABCA1* | 9 | 107618456 | A/G | 0.01 | 0.01 | 0 | Intron |
| rs73663573 | *ABCA1* | 9 | 107618535 | A/T | 0.22 | 0.25 | 0 | Intron |
| rs2472384 | *ABCA1* | 9 | 107618681 | T/C | 0.4 | 0.72 | 0.24 | Intron |
| rs73521841 | *ABCA1* | 9 | 107618734 | C/T | 0.34 | 0.44 | 0.0013 | Intron |
| rs2253304 | *ABCA1* | 9 | 107619063 | G/A | 0.27 | 0.85 | 0.25 | Intron |
| rs4506285 | *ABCA1* | 9 | 107619194 | C/T | 0.03 | 0.07 | 0.09 | Intron |
| rs2472383 | *ABCA1* | 9 | 107619266 | T/C | 0.4 | 0.72 | 0.24 | Intron |
| rs115756482 | *ABCA1* | 9 | 107619267 | G/A | 0.03 | 0.08 | 0 | Intron |
| rs2472445 | *ABCA1* | 9 | 107619350 | C/A | 0.05 | 0.07 | 0.13 | Intron |
| rs2472444 | *ABCA1* | 9 | 107619510 | A/G | 0.4 | 0.72 | 0.24 | Intron |
| rs113894174 | *ABCA1* | 9 | 107619516 | G/A | 0.34 | 0.43 | 0.0013 | Intron |
| rs140166525 | *ABCA1* | 9 | 107619539 | G/A | 0.22 | 0.25 | 0 | Intron |
| rs2487055 | *ABCA1* | 9 | 107619620 | C/T | 0.32 | 0.8 | 0.25 | Intron |
| rs144431307 | *ABCA1* | 9 | 107619670 | C/T | 0.03 | 0.07 | 0.01 | Intron |
| rs2472382 | *ABCA1* | 9 | 107619684 | T/G | 0.05 | 0.07 | 0.13 | Intron |
| rs57450049 | *ABCA1* | 9 | 107619746 | C/T | 0.21 | 0.24 | 0 | Intron |
| rs114231421 | *ABCA1* | 9 | 107619763 | G/A | 0.06 | 0.08 | 0.0026 | Intron |
| rs2472441 | *ABCA1* | 9 | 107619808 | T/A | 0.4 | 0.72 | 0.24 | Intron |
| rs2487057 | *ABCA1* | 9 | 107619812 | C/T | 0.4 | 0.72 | 0.24 | Intron |
| 9.107619985 | *ABCA1* | 9 | 107619985 | C/CAA | 0.07 | - | - | Intron |
| rs2253182 | *ABCA1* | 9 | 107620124 | C/G | 0.29 | 0.82 | 0.24 | Intron |
| rs2253175 | *ABCA1* | 9 | 107620299 | C/T | 0.28 | 0.84 | 0.24 | Intron |
| rs2253174 | *ABCA1* | 9 | 107620332 | G/A | 0.32 | 0.8 | 0.24 | Intron |
| rs2230806 | *ABCA1* | 9 | 107620867 | C/T | 0.4 | 0.72 | 0.24 | NonSyn |
| rs9282538 | *ABCA1* | 9 | 107620872 | T/C | 0.01 | 0.01 | 0 | Syn |
| rs115216814 | *ABCA1* | 9 | 107620889 | A/T | 0.01 | 0.02 | 0 | NonSyn |
| rs2243313 | *ABCA1* | 9 | 107621140 | A/C | 0.4 | 0.72 | 0.24 | Intron |
| rs7860348 | *ABCA1* | 9 | 107621457 | C/T | 0.09 | 0.18 | 0 | Intron |
| rs2482420 | *ABCA1* | 9 | 107621638 | A/G | 0.4 | 0.72 | 0.24 | Intron |
| rs4149287 | *ABCA1* | 9 | 107621672 | G/A | 0.02 | 0.01 | 0.03 | Intron |
| rs2472440 | *ABCA1* | 9 | 107621921 | T/C | 0.4 | 0.73 | 0.24 | Intron |
| rs2482421 | *ABCA1* | 9 | 107621977 | A/G | 0.4 | 0.73 | 0.24 | Intron |
| rs2472381 | *ABCA1* | 9 | 107622098 | A/G | 0.4 | 0.73 | 0.24 | Intron |
| rs2472439 | *ABCA1* | 9 | 107622483 | A/G | 0.4 | 0.73 | 0.24 | Intron |
| rs2472438 | *ABCA1* | 9 | 107622498 | A/C | 0.4 | 0.73 | 0.24 | Intron |
| rs141574863 | *ABCA1* | 9 | 107622714 | A/C | 0.04 | 0.09 | 0 | Intron |
| rs2472437 | *ABCA1* | 9 | 107622832 | C/T | 0.4 | 0.72 | 0.24 | Intron |
| rs116171343 | *ABCA1* | 9 | 107622883 | A/C | 0.01 | 0.04 | 0 | Intron |
| rs79872480 | *ABCA1* | 9 | 107622901 | A/G | 0.04 | 0.09 | 0 | Intron |
| rs139044915 | *ABCA1* | 9 | 107623024 | T/G | 0.01 | 0.01 | 0 | Intron |
| rs2482422 | *ABCA1* | 9 | 107623094 | A/G | 0.4 | 0.69 | 0.21 | Intron |
| rs2472436 | *ABCA1* | 9 | 107623100 | T/C | 0.4 | 0.69 | 0.21 | Intron |
| rs2472435 | *ABCA1* | 9 | 107623109 | C/T | 0.4 | 0.7 | 0.21 | Intron |
| rs144449874 | *ABCA1* | 9 | 107623124 | G/A | 0.02 | 0.02 | 0.0013 | Intron |
| rs2472434 | *ABCA1* | 9 | 107623249 | A/C | 0.4 | 0.71 | 0.25 | Intron |
| rs142678973 | *ABCA1* | 9 | 107623295 | C/T | 0.06 | 0.07 | 0.0026 | Intron |
| rs2472433 | *ABCA1* | 9 | 107623326 | C/T | 0.4 | 0.73 | 0.24 | Intron |
| rs74526913 | *ABCA1* | 9 | 107623378 | C/T | 0.06 | 0.07 | 0.0026 | Intron |
| rs73663575 | *ABCA1* | 9 | 107623514 | G/A | 0.03 | 0.03 | 0 | Intron |
| rs2472378 | *ABCA1* | 9 | 107623570 | G/T | 0.4 | 0.73 | 0.23 | Intron |
| rs2487058 | *ABCA1* | 9 | 107623626 | C/T | 0.4 | 0.73 | 0.23 | Intron |
| rs10114399 | *ABCA1* | 9 | 107623653 | T/A | 0.39 | 0.47 | 0.0013 | Intron |
| rs73504110 | *ABCA1* | 9 | 107623807 | G/C | 0.11 | 0.16 | 0 | Intron |
| rs2230805 | *ABCA1* | 9 | 107624029 | C/T | 0.42 | 0.49 | 0.23 | Syn |
| rs2249891 | *ABCA1* | 9 | 107624242 | A/G | 0.41 | 0.7 | 0.11 | Intron |
| rs2487060 | *ABCA1* | 9 | 107624383 | T/C | 0.16 | 0.21 | 0.11 | Intron |
| rs2487061 | *ABCA1* | 9 | 107624389 | T/C | 0.16 | 0.21 | 0.11 | Intron |
| rs189151148 | *ABCA1* | 9 | 107624923 | G/C | 0.08 | 0.1 | 0.07 | Intron |
| rs182548880 | *ABCA1* | 9 | 107624928 | G/C | 0.04 | 0.07 | 0.02 | Intron |
| rs1831554 | *ABCA1* | 9 | 107625312 | G/A | 0.06 | 0.04 | 0.03 | Intron |
| rs4149286 | *ABCA1* | 9 | 107625803 | G/A | 0.06 | 0.04 | 0.03 | Intron |
| rs4149285 | *ABCA1* | 9 | 107625932 | A/G | 0.1 | 0.1 | 0.03 | Intron |
| rs4149284 | *ABCA1* | 9 | 107626108 | G/A | 0.04 | 0.03 | 0.03 | Intron |
| rs4149283 | *ABCA1* | 9 | 107626189 | G/T | 0.06 | 0.04 | 0.03 | Intron |
| rs4149282 | *ABCA1* | 9 | 107626284 | A/G | 0.09 | 0.09 | 0.03 | Intron |
| rs4149281 | *ABCA1* | 9 | 107626389 | C/T | 0.47 | 0.51 | 0.12 | Intron |
| rs55666820 | *ABCA1* | 9 | 107626489 | A/G | 0.05 | 0.09 | 0 | Intron |
| rs4149280 | *ABCA1* | 9 | 107626542 | C/T | 0.09 | 0.11 | 0.02 | Intron |
| rs61340780 | *ABCA1* | 9 | 107626900 | G/A | 0.02 | 0.07 | 0.01 | Intron |
| rs114415472 | *ABCA1* | 9 | 107627648 | T/C | 0.03 | 0.03 | 0 | Intron |
| rs116144254 | *ABCA1* | 9 | 107627974 | A/C | 0.03 | 0.08 | 0 | Intron |
| rs114125287 | *ABCA1* | 9 | 107627987 | A/C | 0.03 | 0.08 | 0 | Intron |
| rs75232950 | *ABCA1* | 9 | 107628375 | G/C | 0.03 | 0.07 | 0 | Intron |
| rs80272611 | *ABCA1* | 9 | 107628403 | C/T | 0.03 | 0.04 | 0 | Intron |
| rs77182112 | *ABCA1* | 9 | 107628436 | T/C | 0.03 | 0.07 | 0 | Intron |
| rs75400479 | *ABCA1* | 9 | 107628712 | T/C | 0.06 | 0.16 | 0.0026 | Intron |
| rs72732692 | *ABCA1* | 9 | 107628733 | A/C | 0.07 | 0.17 | 0.04 | Intron |
| rs72732693 | *ABCA1* | 9 | 107628779 | T/A | 0.07 | 0.16 | 0.05 | Intron |
| rs111373539 | *ABCA1* | 9 | 107628892 | T/A | 0.1 | 0.2 | 0.03 | Intron |
| rs140563773 | *ABCA1* | 9 | 107629034 | G/T | 0.03 | 0.07 | 0 | Intron |
| rs12004282 | *ABCA1* | 9 | 107629058 | C/T | 0.04 | 0.1 | 0.16 | Intron |
| rs4743764 | *ABCA1* | 9 | 107629104 | T/C | 0.2 | 0.83 | 0.39 | Intron |
| rs13301006 | *ABCA1* | 9 | 107629336 | C/T | 0.05 | 0.1 | 0.14 | Intron |
| rs72732694 | *ABCA1* | 9 | 107629601 | C/A | 0.07 | 0.14 | 0.08 | Intron |
| rs117860433 | *ABCA1* | 9 | 107630430 | C/T | 0.04 | 0.07 | 0.05 | Intron |
| rs10991386 | *ABCA1* | 9 | 107630433 | A/G | 0.24 | 0.81 | 0.41 | Intron |
| rs141743020 | *ABCA1* | 9 | 107630452 | C/T | 0.03 | 0.05 | 0 | Intron |
| rs139665032 | *ABCA1* | 9 | 107630512 | T/C | 0.03 | 0.05 | 0 | Intron |
| rs112460067 | *ABCA1* | 9 | 107630570 | T/C | 0.09 | 0.1 | 0.03 | Intron |
| rs146744108 | *ABCA1* | 9 | 107630582 | G/A | 0.04 | 0.07 | 0.05 | Intron |
| rs13291032 | *ABCA1* | 9 | 107630620 | C/T | 0.07 | 0.14 | 0.18 | Intron |
| rs10118061 | *ABCA1* | 9 | 107631134 | G/A | 0.19 | 0.25 | 0.18 | Intron |
| rs34650643 | *ABCA1* | 9 | 107631312 | G/GA | 0.07 | 0.13 | 0.18 | Intron |
| rs111962753 | *ABCA1* | 9 | 107631907 | G/A | 0.03 | 0.05 | 0 | Intron |
| rs10991387 | *ABCA1* | 9 | 107632157 | A/G | 0.1 | 0.12 | 0 | Intron |
| rs28714441 | *ABCA1* | 9 | 107632321 | T/C | 0.1 | 0.12 | 0 | Intron |
| rs10583466 | *ABCA1* | 9 | 107632401 | CAG/C | 0.07 | 0.13 | 0.18 | Intron |
| rs61602310 | *ABCA1* | 9 | 107632615 | A/G | 0.02 | 0.03 | 0 | Intron |
| rs74955922 | *ABCA1* | 9 | 107632623 | C/T | 0.11 | 0.18 | 0 | Intron |
| rs11791258 | *ABCA1* | 9 | 107632644 | G/A | 0.07 | 0.13 | 0.18 | Intron |
| rs4149276 | *ABCA1* | 9 | 107632660 | C/G | 0.14 | 0.18 | 0.17 | Intron |
| rs114163629 | *ABCA1* | 9 | 107632661 | G/A | 0.09 | 0.16 | 0 | Intron |
| rs201670638 | *ABCA1* | 9 | 107632932 | A/AG | 0.03 | 0.05 | 0 | Intron |
| rs200587029 | *ABCA1* | 9 | 107632935 | T/TC | 0.03 | 0.05 | 0 | Intron |
| rs61337787 | *ABCA1* | 9 | 107632953 | C/T | 0.07 | 0.1 | 0 | Intron |
| rs1929842 | *ABCA1* | 9 | 107633623 | G/C | 0.38 | 0.68 | 0.22 | Intron |
| rs1929841 | *ABCA1* | 9 | 107633725 | A/C | 0.17 | 0.21 | 0.18 | Intron |
| rs1340355 | *ABCA1* | 9 | 107633777 | C/A | 0.04 | 0.1 | 0.0013 | Intron |
| rs57353097 | *ABCA1* | 9 | 107634102 | G/C | 0.08 | 0.11 | 0.05 | Intron |
| rs143281719 | *ABCA1* | 9 | 107634324 | AT/A | 0.04 | 0.07 | 0 | Intron |
| rs111822745 | *ABCA1* | 9 | 107634371 | T/A | 0.04 | 0.07 | 0 | Intron |
| rs80291013 | *ABCA1* | 9 | 107634401 | A/G | 0.07 | 0.09 | 0 | Intron |
| rs4742927 | *ABCA1* | 9 | 107634640 | G/A | 0.4 | 0.68 | 0.22 | Intron |
| rs76749456 | *ABCA1* | 9 | 107634691 | A/G | 0.04 | 0.07 | 0 | Intron |
| rs28558214 | *ABCA1* | 9 | 107634783 | A/G | 0.24 | 0.27 | 0.05 | Intron |
| rs80022781 | *ABCA1* | 9 | 107634831 | G/C | 0.05 | 0.08 | 0 | Intron |
| rs73504136 | *ABCA1* | 9 | 107635507 | T/C | 0.1 | 0.1 | 0.03 | Intron |
| rs115257506 | *ABCA1* | 9 | 107635708 | C/T | 0.02 | 0.03 | 0 | Intron |
| rs80192261 | *ABCA1* | 9 | 107635724 | G/A | 0.03 | 0.08 | 0 | Intron |
| rs2000069 | *ABCA1* | 9 | 107635869 | C/T | 0.18 | 0.92 | 0.42 | Intron |
| rs111919605 | *ABCA1* | 9 | 107636559 | G/A | 0.14 | 0.14 | 0.18 | Intron |
| rs55785018 | *ABCA1* | 9 | 107636676 | C/T | 0.02 | 0.03 | 0 | Intron |
| rs13285112 | *ABCA1* | 9 | 107636707 | C/A | 0.18 | 0.92 | 0.42 | Intron |
| rs141765508 | *ABCA1* | 9 | 107636736 | T/C | 0.03 | 0.07 | 0.01 | Intron |
| rs115327636 | *ABCA1* | 9 | 107636807 | G/A | 0.03 | 0.05 | 0.0013 | Intron |
| rs138153498 | *ABCA1* | 9 | 107636845 | C/T | 0.03 | 0.08 | 0 | Intron |
| rs182499621 | *ABCA1* | 9 | 107636968 | G/A | 0.03 | 0.07 | 0.0026 | Intron |
| rs191098995 | *ABCA1* | 9 | 107637069 | C/T | 0.04 | 0.08 | 0.03 | Intron |
| rs142663568 | *ABCA1* | 9 | 107637169 | G/A | 0.11 | 0.16 | 0.05 | Intron |
| rs59237458 | *ABCA1* | 9 | 107637306 | A/G | 0.18 | 0.91 | 0.42 | Intron |
| rs148509765 | *ABCA1* | 9 | 107637370 | G/T | 0.03 | 0.08 | 0.004 | Intron |
| rs150646225 | *ABCA1* | 9 | 107637492 | C/T | 0.01 | 0.02 | 0 | Intron |
| rs140779255 | *ABCA1* | 9 | 107637608 | G/A | 0.02 | 0.06 | 0 | Intron |
| rs144856495 | *ABCA1* | 9 | 107637625 | T/A | 0.02 | 0.03 | 0 | Intron |
| rs12346461 | *ABCA1* | 9 | 107637863 | A/C | 0.16 | 0.21 | 0.05 | Intron |
| rs76244174 | *ABCA1* | 9 | 107637937 | A/T | 0.08 | 0.11 | 0.05 | Intron |
| rs12350745 | *ABCA1* | 9 | 107638062 | G/C | 0.06 | 0.09 | 0.05 | Intron |
| rs4149275 | *ABCA1* | 9 | 107638507 | A/G | 0.11 | 0.07 | 0.17 | Intron |
| rs4149343 | *ABCA1* | 9 | 107638969 | AG/A | 0.16 | 0.23 | 0.11 | Intron |
| rs67740686 | *ABCA1* | 9 | 107639180 | G/A | 0.06 | 0.09 | 0.1 | Intron |
| rs75516297 | *ABCA1* | 9 | 107639297 | A/C | 0.05 | 0.05 | 0.0026 | Intron |
| rs4149274 | *ABCA1* | 9 | 107639414 | G/A | 0.24 | 0.27 | 0.29 | Intron |
| rs116596238 | *ABCA1* | 9 | 107640307 | G/A | 0.03 | 0.02 | 0 | Intron |
| rs75906492 | *ABCA1* | 9 | 107640625 | T/G | 0.07 | 0.07 | 0 | Intron |
| rs1999430 | *ABCA1* | 9 | 107640688 | G/A | 0.19 | 0.22 | 0.03 | Intron |
| rs1999429 | *ABCA1* | 9 | 107640786 | G/T | 0.27 | 0.33 | 0.03 | Intron |
| rs114067526 | *ABCA1* | 9 | 107640810 | A/G | 0.03 | 0.07 | 0 | Intron |
| rs80233998 | *ABCA1* | 9 | 107641741 | G/T | 0.12 | 0.12 | 0.0013 | Intron |
| rs56948527 | *ABCA1* | 9 | 107642019 | A/G | 0.27 | 0.33 | 0.03 | Intron |
| rs4149273 | *ABCA1* | 9 | 107642211 | C/T | 0.29 | 0.73 | 0.34 | Intron |
| rs4149272 | *ABCA1* | 9 | 107642287 | C/T | 0.25 | 0.81 | 0.34 | Intron |
| rs12001061 | *ABCA1* | 9 | 107643080 | T/A | 0.3 | 0.75 | 0.34 | Intron |
| rs12006437 | *ABCA1* | 9 | 107643120 | C/A | 0.26 | 0.3 | 0.01 | Intron |
| rs12002435 | *ABCA1* | 9 | 107643299 | G/C | 0.26 | 0.31 | 0.01 | Intron |
| rs148281698 | *ABCA1* | 9 | 107643361 | C/A | 0.06 | 0.06 | 0.02 | Intron |
| rs139025219 | *ABCA1* | 9 | 107643590 | T/C | 0.05 | 0.06 | 0.02 | Intron |
| rs200260433 | *ABCA1* | 9 | 107643755 | CA/C | 0.05 | 0.06 | 0.02 | Intron |
| rs62568179 | *ABCA1* | 9 | 107643786 | G/A | 0.2 | 0.22 | 0.12 | Intron |
| rs11790326 | *ABCA1* | 9 | 107643810 | T/C | 0.21 | 0.18 | 0.2 | Intron |
| rs7859265 | *ABCA1* | 9 | 107644124 | G/C | 0.22 | 0.22 | 0.01 | Intron |
| rs115950766 | *ABCA1* | 9 | 107644138 | A/T | 0.06 | 0.06 | 0.02 | Intron |
| rs4149271 | *ABCA1* | 9 | 107644287 | G/A | 0.17 | 0.15 | 0.2 | Intron |
| rs79252500 | *ABCA1* | 9 | 107644551 | C/T | 0.03 | 0.07 | 0 | Intron |
| rs76658744 | *ABCA1* | 9 | 107644705 | T/C | 0.03 | 0.07 | 0 | Intron |
| rs3858074 | *ABCA1* | 9 | 107644737 | A/C | 0.17 | 0.15 | 0.2 | Intron |
| rs3858075 | *ABCA1* | 9 | 107644830 | C/T | 0.17 | 0.15 | 0.2 | Intron |
| rs115568881 | *ABCA1* | 9 | 107645059 | G/T | 0.01 | 0.02 | 0 | Intron |
| rs2275542 | *ABCA1* | 9 | 107645080 | C/T | 0.24 | 0.24 | 0.29 | Intron |
| rs76838353 | *ABCA1* | 9 | 107645210 | C/T | 0.09 | 0.07 | 0.0013 | Intron |
| rs41419649 | *ABCA1* | 9 | 107645244 | A/C | 0.05 | 0.06 | 0.02 | Intron |
| rs143894099 | *ABCA1* | 9 | 107645295 | G/A | 0.03 | 0.07 | 0 | Intron |
| rs12003906 | *ABCA1* | 9 | 107645477 | G/T | 0.21 | 0.2 | 0.004 | Intron |
| rs62568181 | *ABCA1* | 9 | 107645674 | T/C | 0.07 | 0.09 | 0.09 | Intron |
| rs7040990 | *ABCA1* | 9 | 107645906 | C/G | 0.19 | 0.16 | 0.004 | Intron |
| rs13283515 | *ABCA1* | 9 | 107645922 | T/C | 0.07 | 0.09 | 0.09 | Intron |
| rs35204953 | *ABCA1* | 9 | 107645972 | C/CT | 0.17 | 0.15 | 0.2 | Intron |
| rs3904997 | *ABCA1* | 9 | 107646009 | G/A | 0.17 | 0.15 | 0.2 | Intron |
| rs7041412 | *ABCA1* | 9 | 107646224 | C/A | 0.05 | 0.04 | 0.0013 | Intron |
| rs3904998 | *ABCA1* | 9 | 107646260 | T/C | 0.17 | 0.15 | 0.2 | Intron |
| rs3904999 | *ABCA1* | 9 | 107646289 | C/T | 0.17 | 0.15 | 0.2 | Intron |
| rs11789603 | *ABCA1* | 9 | 107647019 | C/T | 0.17 | 0.14 | 0.09 | Intron |
| rs4149270 | *ABCA1* | 9 | 107647077 | C/T | 0.24 | 0.24 | 0.3 | Intron |
| rs75602357 | *ABCA1* | 9 | 107647106 | C/T | 0.06 | 0.06 | 0.02 | Intron |
| rs4149269 | *ABCA1* | 9 | 107647121 | A/G | 0.46 | 0.59 | 0.34 | Intron |
| rs4149268 | *ABCA1* | 9 | 107647220 | C/T | 0.33 | 0.71 | 0.35 | Intron |
| rs145562918 | *ABCA1* | 9 | 107647252 | C/T | 0.03 | 0.03 | 0 | Intron |
| rs7046079 | *ABCA1* | 9 | 107647504 | C/T | 0.19 | 0.18 | 0.02 | Intron |
| rs3890182 | *ABCA1* | 9 | 107647655 | G/A | 0.13 | 0.14 | 0.12 | Intron |
| rs143210459 | *ABCA1* | 9 | 107647760 | AAC/A | 0.13 | 0.14 | 0.12 | Intron |
| rs78853242 | *ABCA1* | 9 | 107647973 | C/T | 0.07 | 0.07 | 0 | Intron |
| rs76549234 | *ABCA1* | 9 | 107648328 | G/C | 0.03 | 0.07 | 0 | Intron |
| rs72732702 | *ABCA1* | 9 | 107648409 | G/A | 0.17 | 0.15 | 0.2 | Intron |
| rs3847300 | *ABCA1* | 9 | 107648430 | G/A | 0.13 | 0.14 | 0.12 | Intron |
| rs2417565 | *ABCA1* | 9 | 107648458 | G/A | 0.23 | 0.27 | 0.12 | Intron |
| rs201371747 | *ABCA1* | 9 | 107648476 | TC/T | 0.17 | 0.15 | 0.2 | Intron |
| rs202138068 | *ABCA1* | 9 | 107648478 | CT/C | 0.17 | 0.15 | 0.2 | Intron |
| rs3847301 | *ABCA1* | 9 | 107648519 | T/C | 0.23 | 0.27 | 0.12 | Intron |
| rs114757628 | *ABCA1* | 9 | 107648555 | C/A | 0.02 | 0.05 | 0.0013 | Intron |
| rs3847302 | *ABCA1* | 9 | 107648595 | A/G | 0.12 | 0.14 | 0.12 | Intron |
| rs3847303 | *ABCA1* | 9 | 107648652 | C/T | 0.23 | 0.27 | 0.12 | Intron |
| rs13290420 | *ABCA1* | 9 | 107648965 | T/C | 0.26 | 0.29 | 0.12 | Intron |
| rs79648177 | *ABCA1* | 9 | 107649095 | T/C | 0.03 | 0.07 | 0 | Intron |
| rs144006067 | *ABCA1* | 9 | 107649152 | G/C | 0.02 | - | - | Intron |
| rs13286991 | *ABCA1* | 9 | 107649380 | A/G | 0.26 | 0.29 | 0.12 | Intron |
| rs13286395 | *ABCA1* | 9 | 107649419 | C/T | 0.26 | 0.29 | 0.12 | Intron |
| rs112238361 | *ABCA1* | 9 | 107649557 | A/AAAAC | 0.26 | 0.29 | 0.12 | Intron |
| rs7027196 | *ABCA1* | 9 | 107649606 | C/T | 0.26 | 0.29 | 0.12 | Intron |
| rs7043081 | *ABCA1* | 9 | 107649611 | A/T | 0.26 | 0.29 | 0.12 | Intron |
| 9.107649731 | *ABCA1* | 9 | 107649731 | C/CA | 0.26 | - | - | Intron |
| rs7026408 | *ABCA1* | 9 | 107649758 | G/A | 0.02 | 0.04 | 0 | Intron |
| rs77546802 | *ABCA1* | 9 | 107649898 | C/T | 0.03 | 0.07 | 0 | Intron |
| rs7043581 | *ABCA1* | 9 | 107649984 | A/G | 0.26 | 0.3 | 0.12 | Intron |
| rs113206627 | *ABCA1* | 9 | 107650033 | A/G | 0.07 | 0.09 | 0.09 | Intron |
| rs116564772 | *ABCA1* | 9 | 107650282 | G/A | 0.02 | 0.04 | 0 | Intron |
| rs34171271 | *ABCA1* | 9 | 107650317 | T/C | 0.26 | 0.29 | 0.12 | Intron |
| rs201879057 | *ABCA1* | 9 | 107650341 | T/TGG | 0.23 | 0.27 | 0.09 | Intron |
| rs62568183 | *ABCA1* | 9 | 107650345 | T/G | 0.13 | 0.15 | 0.12 | Intron |
| rs62568184 | *ABCA1* | 9 | 107650355 | T/G | 0.12 | 0.14 | 0.12 | Intron |
| rs13292582 | *ABCA1* | 9 | 107650430 | A/G | 0.27 | 0.3 | 0.12 | Intron |
| rs7047523 | *ABCA1* | 9 | 107650575 | T/C | 0.31 | 0.73 | 0.35 | Intron |
| rs2791948 | *ABCA1* | 9 | 107650578 | G/C | 0.03 | 0.01 | 0.18 | Intron |
| rs35699471 | *ABCA1* | 9 | 107650820 | C/T | 0.07 | 0.1 | 0.11 | Intron |
| rs10115928 | *ABCA1* | 9 | 107650843 | C/T | 0.27 | 0.77 | 0.35 | Intron |
| rs115720069 | *ABCA1* | 9 | 107651164 | T/C | 0.02 | 0.04 | 0 | Intron |
| rs2275543 | *ABCA1* | 9 | 107651174 | T/C | 0.07 | 0.09 | 0.09 | Intron |
| rs2275544 | *ABCA1* | 9 | 107651212 | T/C | 0.15 | 0.23 | 0.13 | Intron |
| rs10512334 | *ABCA1* | 9 | 107651232 | T/C | 0.09 | 0.07 | 0.0013 | Intron |
| rs7341705 | *ABCA1* | 9 | 107651348 | T/C | 0.06 | 0.08 | 0 | Intron |
| rs2275545 | *ABCA1* | 9 | 107651526 | A/G | 0.28 | 0.35 | 0.14 | Intron |
| rs41537052 | *ABCA1* | 9 | 107651534 | C/T | 0.06 | 0.08 | 0 | Intron |
| rs4149267 | *ABCA1* | 9 | 107651945 | T/C | 0.27 | 0.77 | 0.35 | Intron |
| rs73504175 | *ABCA1* | 9 | 107652341 | G/C | 0.19 | 0.16 | 0.004 | Intron |
| rs115496950 | *ABCA1* | 9 | 107652474 | C/A | 0.04 | 0.04 | 0 | Intron |
| rs111295834 | *ABCA1* | 9 | 107652492 | C/T | 0.06 | 0.08 | 0 | Intron |
| rs80197000 | *ABCA1* | 9 | 107652980 | A/G | 0.03 | 0.07 | 0 | Intron |
| rs12686004 | *ABCA1* | 9 | 107653426 | G/A | 0.03 | 0.02 | 0.11 | Intron |
| rs111733792 | *ABCA1* | 9 | 107653677 | C/T | 0.06 | 0.08 | 0 | Intron |
| rs58065385 | *ABCA1* | 9 | 107653957 | C/G | 0.13 | 0.11 | 0.0026 | Intron |
| rs145842792 | *ABCA1* | 9 | 107654006 | C/T | 0.03 | 0.06 | 0.0026 | Intron |
| rs138405364 | *ABCA1* | 9 | 107654103 | G/A | 0.12 | 0.14 | 0.02 | Intron |
| rs142914080 | *ABCA1* | 9 | 107654225 | G/A | 0.06 | 0.08 | 0.0026 | Intron |
| rs115464783 | *ABCA1* | 9 | 107654293 | C/T | 0.03 | 0.08 | 0.01 | Intron |
| rs145406734 | *ABCA1* | 9 | 107654358 | ACT/A | 0.03 | 0.07 | 0 | Intron |
| rs57877911 | *ABCA1* | 9 | 107654765 | G/A | 0.06 | 0.08 | 0 | Intron |
| rs58724784 | *ABCA1* | 9 | 107654810 | C/T | 0.06 | 0.08 | 0 | Intron |
| rs58091028 | *ABCA1* | 9 | 107654956 | A/G | 0.06 | 0.08 | 0 | Intron |
| rs59501244 | *ABCA1* | 9 | 107655008 | GT/G | 0.06 | 0.08 | 0 | Intron |
| rs79674744 | *ABCA1* | 9 | 107655149 | A/T | 0.06 | 0.06 | 0.02 | Intron |
| rs76764024 | *ABCA1* | 9 | 107655154 | T/C | 0.03 | 0.04 | 0 | Intron |
| rs10125482 | *ABCA1* | 9 | 107655426 | G/T | 0.12 | 0.14 | 0.11 | Intron |
| rs113352206 | *ABCA1* | 9 | 107655540 | G/A | 0.07 | 0.09 | 0 | Intron |
| rs112263248 | *ABCA1* | 9 | 107655680 | G/A | 0.06 | 0.08 | 0 | Intron |
| rs3847304 | *ABCA1* | 9 | 107655848 | T/C | 0.17 | 0.15 | 0.2 | Intron |
| rs76304226 | *ABCA1* | 9 | 107655953 | T/C | 0.02 | 0.02 | 0 | Intron |
| rs77258145 | *ABCA1* | 9 | 107656014 | C/A | 0.06 | 0.08 | 0 | Intron |
| rs3858076 | *ABCA1* | 9 | 107656122 | A/C | 0.34 | 0.41 | 0.14 | Intron |
| rs2740494 | *ABCA1* | 9 | 107656150 | G/C | 0.27 | 0.77 | 0.35 | Intron |
| rs79433302 | *ABCA1* | 9 | 107656293 | C/T | 0.06 | 0.06 | 0.02 | Intron |
| rs11304201 | *ABCA1* | 9 | 107656409 | GC/G | 0.2 | 0.26 | 0.13 | Intron |
| rs78727327 | *ABCA1* | 9 | 107656573 | G/A | 0.04 | 0.04 | 0 | Intron |
| rs112145009 | *ABCA1* | 9 | 107656783 | C/T | 0.06 | 0.08 | 0 | Intron |
| rs3905000 | *ABCA1* | 9 | 107657070 | G/A | 0.16 | 0.23 | 0.13 | Intron |
| rs3847305 | *ABCA1* | 9 | 107657253 | G/C | 0.49 | 0.58 | 0.14 | Intron |
| rs7848844 | *ABCA1* | 9 | 107657356 | A/G | 0.28 | 0.26 | 0.004 | Intron |
| rs138960174 | *ABCA1* | 9 | 107657647 | T/C | 0.01 | 0.02 | 0 | Intron |
| rs2740493 | *ABCA1* | 9 | 107657825 | C/T | 0.27 | 0.77 | 0.34 | Intron |
| rs79271694 | *ABCA1* | 9 | 107657986 | A/C | 0.09 | 0.11 | 0 | Intron |
| rs115365004 | *ABCA1* | 9 | 107657990 | T/C | 0.03 | 0.07 | 0 | Intron |
| rs2740492 | *ABCA1* | 9 | 107658248 | C/T | 0.41 | 0.36 | 0.2 | Intron |
| rs75494727 | *ABCA1* | 9 | 107658339 | T/C | 0.07 | 0.07 | 0 | Intron |
| rs2740491 | *ABCA1* | 9 | 107658385 | G/A | 0.27 | 0.77 | 0.34 | Intron |
| rs12341993 | *ABCA1* | 9 | 107658910 | C/A | 0.25 | 0.34 | 0.11 | Intron |
| rs80170476 | *ABCA1* | 9 | 107659084 | T/A | 0.08 | 0.11 | 0.02 | Intron |
| rs3983644 | *ABCA1* | 9 | 107659093 | C/T | 0.26 | 0.24 | 0.2 | Intron |
| rs2027403 | *ABCA1* | 9 | 107659105 | G/A | 0.35 | 0.73 | 0.35 | Intron |
| rs200295108 | *ABCA1* | 9 | 107659409 | GA/G | 0.02 | 0.03 | 0 | Intron |
| rs56000254 | *ABCA1* | 9 | 107659978 | G/A | 0.01 | 0.02 | 0 | Intron |
| rs201966762 | *ABCA1* | 9 | 107660431 | GATT/G | 0.03 | 0.04 | 0 | Intron |
| rs114489744 | *ABCA1* | 9 | 107660434 | T/G | 0.03 | 0.04 | 0 | Intron |
| rs2099544 | *ABCA1* | 9 | 107660780 | A/T | 0.3 | 0.76 | 0.36 | Intron |
| rs73504185 | *ABCA1* | 9 | 107660938 | T/C | 0.05 | 0.05 | 0 | Intron |
| rs2777784 | *ABCA1* | 9 | 107661059 | A/G | 0.29 | 0.77 | 0.36 | Intron |
| rs60554907 | *ABCA1* | 9 | 107661086 | T/C | 0.06 | 0.07 | 0 | Intron |
| rs2777785 | *ABCA1* | 9 | 107661122 | A/G | 0.29 | 0.78 | 0.36 | Intron |
| rs2244278 | *ABCA1* | 9 | 107661129 | C/A | 0.03 | 0.01 | 0.12 | Intron |
| rs10120087 | *ABCA1* | 9 | 107661150 | C/A | 0.33 | 0.31 | 0.1 | Intron |
| rs199941380 | *ABCA1* | 9 | 107661520 | G/GA | 0.03 | 0.03 | 0 | Intron |
| rs2777786 | *ABCA1* | 9 | 107661561 | C/G | 0.24 | 0.81 | 0.36 | Intron |
| rs2777787 | *ABCA1* | 9 | 107661735 | A/G | 0.26 | 0.78 | 0.33 | Intron |
| rs2740488 | *ABCA1* | 9 | 107661742 | A/C | 0.38 | 0.46 | 0.24 | Intron |
| rs147115468 | *ABCA1* | 9 | 107661953 | C/T | 0.07 | 0.07 | 0 | Intron |
| rs200997916 | *ABCA1* | 9 | 107662303 | AG/A | 0.08 | 0.09 | 0.06 | Intron |
| rs73664373 | *ABCA1* | 9 | 107662304 | G/C | 0.08 | 0.09 | 0.07 | Intron |
| rs73664374 | *ABCA1* | 9 | 107662305 | C/G | 0.08 | 0.09 | 0.07 | Intron |
| rs2575875 | *ABCA1* | 9 | 107662494 | G/A | 0.33 | 0.74 | 0.35 | Intron |
| rs2791950 | *ABCA1* | 9 | 107662691 | C/T | 0.3 | 0.77 | 0.36 | Intron |
| rs2777788 | *ABCA1* | 9 | 107662701 | G/A | 0.3 | 0.78 | 0.35 | Intron |
| rs4149266 | *ABCA1* | 9 | 107662705 | G/A | 0.3 | 0.78 | 0.35 | Intron |
| rs2777789 | *ABCA1* | 9 | 107662708 | T/C | 0.3 | 0.78 | 0.35 | Intron |
| rs61319799 | *ABCA1* | 9 | 107662812 | A/G | 0.28 | 0.26 | 0.1 | Intron |
| rs2777790 | *ABCA1* | 9 | 107663039 | C/T | 0.29 | 0.78 | 0.35 | Intron |
| rs2777791 | *ABCA1* | 9 | 107663065 | C/G | 0.29 | 0.78 | 0.35 | Intron |
| rs2777792 | *ABCA1* | 9 | 107663094 | C/T | 0.29 | 0.78 | 0.35 | Intron |
| rs79794931 | *ABCA1* | 9 | 107663644 | C/T | 0.03 | 0.04 | 0 | Intron |
| rs77083720 | *ABCA1* | 9 | 107663762 | G/A | 0.06 | 0.1 | 0.01 | Intron |
| rs1883025 | *ABCA1* | 9 | 107664301 | C/T | 0.31 | 0.38 | 0.24 | Intron |
| rs73664375 | *ABCA1* | 9 | 107664373 | C/T | 0.06 | 0.06 | 0.07 | Intron |
| rs73506109 | *ABCA1* | 9 | 107664475 | A/T | 0.04 | 0.03 | 0.0013 | Intron |
| rs3758294 | *ABCA1* | 9 | 107664815 | T/C | 0.41 | 0.4 | 0.2 | Intron |
| rs2740487 | *ABCA1* | 9 | 107664961 | A/G | 0.23 | 0.82 | 0.52 | Intron |
| rs2777793 | *ABCA1* | 9 | 107665511 | C/T | 0.38 | 0.7 | 0.45 | Intron |
| rs2575876 | *ABCA1* | 9 | 107665739 | G/A | 0.18 | 0.25 | 0.24 | Intron |
| rs1800978 | *ABCA1* | 9 | 107665978 | C/G | 0.03 | 0.01 | 0.13 | Intron |
| rs1799777 | *ABCA1* | 9 | 107666035 | G/GC | 0.03 | - | - | Intron |
| rs2254819 | *ABCA1* | 9 | 107666414 | T/C | 0.37 | 0.71 | 0.46 | Intron |
| rs2740486 | *ABCA1* | 9 | 107666513 | T/G | 0.31 | 0.75 | 0.46 | Intron |
| rs2575877 | *ABCA1* | 9 | 107666772 | T/C | 0.23 | 0.81 | 0.53 | Intron |
| rs2575878 | *ABCA1* | 9 | 107667079 | T/C | 0.22 | 0.82 | 0.53 | Intron |
| rs2254708 | *ABCA1* | 9 | 107667147 | T/C | 0.1 | 0.13 | 0.16 | Intron |
| rs138844114 | *ABCA1* | 9 | 107667215 | A/AAGGG | 0.08 | 0.11 | 0.02 | Intron |
| rs3905001 | *ABCA1* | 9 | 107668064 | G/C | 0.4 | 0.42 | 0.27 | Intron |
| rs80253084 | *ABCA1* | 9 | 107668067 | C/T | 0.04 | 0.06 | 0.02 | Intron |
| rs200057994 | *ABCA1* | 9 | 107668246 | AG/A | 0.02 | 0.04 | 0 | Intron |
| rs2575879 | *ABCA1* | 9 | 107668825 | G/C | 0.24 | 0.82 | 0.54 | Intron |
| rs13284054 | *ABCA1* | 9 | 107669073 | T/C | 0.1 | 0.19 | 0.12 | Intron |
| rs76316220 | *ABCA1* | 9 | 107669208 | C/G | 0.02 | 0.04 | 0 | Intron |
| rs56848416 | *ABCA1* | 9 | 107669209 | G/A | 0.01 | 0.02 | 0 | Intron |
| rs4100654 | *ABCA1* | 9 | 107669241 | A/G | 0.08 | 0.11 | 0.09 | Intron |
| rs78174930 | *ABCA1* | 9 | 107669299 | G/C | 0.07 | 0.11 | 0.02 | Intron |
| rs76682344 | *ABCA1* | 9 | 107669610 | G/A | 0.01 | 0.04 | 0 | Intron |
| rs41426948 | *ABCA1* | 9 | 107669732 | A/G | 0.08 | 0.11 | 0.02 | Intron |
| rs76365901 | *ABCA1* | 9 | 107670115 | C/T | 0.03 | 0.07 | 0 | Intron |
| rs143427562 | *ABCA1* | 9 | 107670317 | C/T | 0.02 | 0.04 | 0 | Intron |
| rs113310792 | *ABCA1* | 9 | 107670866 | T/C | 0.04 | 0.07 | 0.0026 | Intron |
| rs10820743 | *ABCA1* | 9 | 107671659 | A/G | 0.27 | 0.31 | 0.28 | Intron |
| rs2777795 | *ABCA1* | 9 | 107672365 | G/A | 0.03 | 0.01 | 0.11 | Intron |
| rs4149265 | *ABCA1* | 9 | 107672498 | G/A | 0.17 | 0.17 | 0.28 | Intron |
| rs62568199 | *ABCA1* | 9 | 107672526 | T/G | 0.05 | 0.06 | 0.08 | Intron |
| rs116021025 | *ABCA1* | 9 | 107672688 | T/C | 0.02 | 0.03 | 0 | Intron |
| rs79678596 | *ABCA1* | 9 | 107673309 | T/C | 0.02 | 0.06 | 0.0013 | Intron |
| rs78425153 | *ABCA1* | 9 | 107674169 | G/A | 0.04 | 0.07 | 0 | Intron |
| rs73506129 | *ABCA1* | 9 | 107674433 | A/T | 0.05 | 0.07 | 0.0013 | Intron |
| rs60718644 | *ABCA1* | 9 | 107674710 | C/A | 0.05 | 0.07 | 0.0013 | Intron |
| rs10521071 | *ABCA1* | 9 | 107674890 | A/G | 0.1 | 0.23 | 0.03 | Intron |
| rs2437818 | *ABCA1* | 9 | 107675422 | G/C | 0.16 | 0.22 | 0.06 | Intron |
| rs34165419 | *ABCA1* | 9 | 107676026 | C/T | 0.09 | 0.08 | 0.06 | Intron |
| rs62568200 | *ABCA1* | 9 | 107676577 | A/C | 0.01 | 0.002 | 0.07 | Intron |
| rs1175453 | *ABCA1* | 9 | 107676706 | G/A | 0.09 | 0.06 | 0.25 | Intron |
| rs2575874 | *ABCA1* | 9 | 107676758 | A/G | 0.21 | 0.83 | 0.78 | Intron |
| rs12237054 | *ABCA1* | 9 | 107676940 | A/G | 0.02 | 0.002 | 0.07 | Intron |
| rs150414107 | *ABCA1* | 9 | 107677049 | T/G | 0.01 | 0.02 | 0 | Intron |
| rs4149264 | *ABCA1* | 9 | 107677211 | G/C | 0.15 | 0.14 | 0.2 | Intron |
| rs4149263 | *ABCA1* | 9 | 107677289 | A/G | 0.15 | 0.14 | 0.21 | Intron |
| rs115896485 | *ABCA1* | 9 | 107677353 | C/T | 0.02 | 0.03 | 0 | Intron |
| rs10991404 | *ABCA1* | 9 | 107677482 | G/A | 0.17 | 0.2 | 0.07 | Intron |
| rs10991405 | *ABCA1* | 9 | 107677512 | G/C | 0.1 | 0.1 | 0.07 | Intron |
| rs4149262 | *ABCA1* | 9 | 107677932 | A/T | 0.02 | 0.01 | 0.07 | Intron |
| rs10991406 | *ABCA1* | 9 | 107677935 | T/C | 0.13 | 0.2 | 0.07 | Intron |
| rs149413390 | *ABCA1* | 9 | 107677938 | A/G | 0.02 | 0.02 | 0 | Intron |
| rs10991407 | *ABCA1* | 9 | 107678011 | T/G | 0.13 | 0.2 | 0.07 | Intron |
| rs4149261 | *ABCA1* | 9 | 107678278 | C/T | 0.05 | 0.07 | 0.09 | Intron |
| rs12350560 | *ABCA1* | 9 | 107678446 | G/A | 0.17 | 0.2 | 0.07 | Intron |
| rs77425484 | *ABCA1* | 9 | 107678610 | C/G | 0.03 | 0.04 | 0 | Intron |
| rs12344687 | *ABCA1* | 9 | 107678640 | G/T | 0.15 | 0.2 | 0.07 | Intron |
| rs75193579 | *ABCA1* | 9 | 107678674 | C/T | 0.05 | 0.1 | 0 | Intron |
| rs12335854 | *ABCA1* | 9 | 107678776 | C/T | 0.19 | 0.21 | 0.07 | Intron |
| rs2515618 | *ABCA1* | 9 | 107678797 | C/T | 0.13 | 0.1 | 0.3 | Intron |
| rs75684347 | *ABCA1* | 9 | 107678835 | G/A | 0.01 | 0.01 | 0 | Intron |
| rs10991408 | *ABCA1* | 9 | 107678916 | T/C | 0.02 | 0.01 | 0.07 | Intron |
| rs10991409 | *ABCA1* | 9 | 107679141 | T/C | 0.02 | 0.01 | 0.07 | Intron |
| rs10991410 | *ABCA1* | 9 | 107679200 | G/T | 0.02 | 0.01 | 0.08 | Intron |
| rs73664376 | *ABCA1* | 9 | 107679315 | G/A | 0.1 | 0.1 | 0.03 | Intron |
| rs13291117 | *ABCA1* | 9 | 107679425 | T/G | 0.14 | 0.15 | 0.06 | Intron |
| rs12336969 | *ABCA1* | 9 | 107679500 | C/A | 0.19 | 0.22 | 0.07 | Intron |
| rs75030337 | *ABCA1* | 9 | 107679774 | A/G | 0.19 | 0.22 | 0.07 | Intron |
| rs7849869 | *ABCA1* | 9 | 107679849 | A/G | 0.14 | 0.14 | 0.06 | Intron |
| rs62568201 | *ABCA1* | 9 | 107679854 | G/A | 0.02 | 0.01 | 0.07 | Intron |
| rs11460644 | *ABCA1* | 9 | 107679857 | G/GA | 0.22 | 0.78 | 0.75 | Intron |
| rs146704815 | *ABCA1* | 9 | 107679870 | G/A | 0.17 | 0.19 | 0.07 | Intron |
| rs140275367 | *ABCA1* | 9 | 107679904 | A/G | 0.18 | 0.22 | 0.04 | Intron |
| rs149944280 | *ABCA1* | 9 | 107679997 | T/G | 0.19 | 0.22 | 0.07 | Intron |
| rs79639423 | *ABCA1* | 9 | 107680113 | G/C | 0.19 | 0.22 | 0.07 | Intron |
| rs77755871 | *ABCA1* | 9 | 107680172 | A/G | 0.19 | 0.22 | 0.07 | Intron |
| rs73506142 | *ABCA1* | 9 | 107680244 | T/C | 0.02 | 0.04 | 0 | Intron |
| rs73664377 | *ABCA1* | 9 | 107680307 | T/G | 0.19 | 0.22 | 0.07 | Intron |
| rs6479284 | *ABCA1* | 9 | 107680355 | T/A | 0.14 | 0.14 | 0.06 | Intron |
| rs35445387 | *ABCA1* | 9 | 107680441 | T/TA | 0.36 | 0.34 | 0.59 | Intron |
| rs2437811 | *ABCA1* | 9 | 107680458 | C/T | 0.2 | 0.84 | 0.79 | Intron |
| rs12003756 | *ABCA1* | 9 | 107680553 | G/A | 0.19 | 0.22 | 0.07 | Intron |
| rs10512335 | *ABCA1* | 9 | 107680639 | T/G | 0.11 | 0.14 | 0.06 | Intron |
| rs10512336 | *ABCA1* | 9 | 107680691 | G/C | 0.19 | 0.22 | 0.07 | Intron |
| rs10512337 | *ABCA1* | 9 | 107680730 | G/A | 0.18 | 0.22 | 0.07 | Intron |
| rs2515617 | *ABCA1* | 9 | 107680915 | A/G | 0.36 | 0.34 | 0.6 | Intron |
| rs73664378 | *ABCA1* | 9 | 107680953 | C/A | 0.19 | 0.22 | 0.07 | Intron |
| rs73506147 | *ABCA1* | 9 | 107681194 | G/C | 0.11 | 0.14 | 0.06 | Intron |
| rs12347523 | *ABCA1* | 9 | 107681425 | G/C | 0.27 | 0.34 | 0.07 | Intron |
| rs7872096 | *ABCA1* | 9 | 107681614 | C/T | 0.1 | 0.14 | 0.04 | Intron |
| rs7857983 | *ABCA1* | 9 | 107681688 | A/C | 0.39 | 0.5 | 0.17 | Intron |
| rs12000590 | *ABCA1* | 9 | 107681693 | C/A | 0.1 | 0.11 | 0.03 | Intron |
| rs7872225 | *ABCA1* | 9 | 107681717 | C/A | 0.17 | 0.25 | 0.04 | Intron |
| rs7861141 | *ABCA1* | 9 | 107681763 | T/C | 0.27 | 0.36 | 0.07 | Intron |
| rs149607070 | *ABCA1* | 9 | 107681850 | G/A | 0.04 | 0.07 | 0 | Intron |
| rs67348902 | *ABCA1* | 9 | 107681973 | G/A | 0.13 | 0.13 | 0.06 | Intron |
| rs2515616 | *ABCA1* | 9 | 107681995 | A/G | 0.2 | 0.84 | 0.79 | Intron |
| rs7861459 | *ABCA1* | 9 | 107682012 | T/G | 0.17 | 0.25 | 0.04 | Intron |
| rs62568202 | *ABCA1* | 9 | 107682194 | C/G | 0.07 | 0.08 | 0.09 | Intron |
| rs73664380 | *ABCA1* | 9 | 107682221 | T/C | 0.11 | 0.13 | 0.03 | Intron |
| rs146181053 | *ABCA1* | 9 | 107682397 | C/T | 0.02 | 0.04 | 0 | Intron |
| rs2791952 | *ABCA1* | 9 | 107682517 | T/C | 0.07 | 0.93 | 0.9 | Intron |
| rs6479285 | *ABCA1* | 9 | 107682885 | T/C | 0.29 | 0.37 | 0.07 | Intron |
| rs2472510 | *ABCA1* | 9 | 107683122 | T/G | 0.12 | 0.09 | 0.3 | Intron |
| rs7049195 | *ABCA1* | 9 | 107683124 | G/A | 0.29 | 0.37 | 0.07 | Intron |
| rs78608624 | *ABCA1* | 9 | 107683172 | A/G | 0.11 | 0.12 | 0.03 | Intron |
| rs7035444 | *ABCA1* | 9 | 107683257 | A/T | 0.08 | 0.12 | 0.0013 | Intron |
| rs147859690 | *ABCA1* | 9 | 107683299 | G/A | 0.01 | 0.02 | 0 | Intron |
| 9.107683378 | *ABCA1* | 9 | 107683378 | G/GA | 0.08 | - | - | Intron |
| rs7035693 | *ABCA1* | 9 | 107683465 | A/G | 0.29 | 0.37 | 0.07 | Intron |
| rs12347858 | *ABCA1* | 9 | 107683524 | T/C | 0.29 | 0.37 | 0.07 | Intron |
| rs12342233 | *ABCA1* | 9 | 107683594 | C/A | 0.04 | 0.16 | 0.04 | Intron |
| rs10512338 | *ABCA1* | 9 | 107683636 | T/C | 0.29 | 0.38 | 0.07 | Intron |
| rs2515615 | *ABCA1* | 9 | 107683692 | C/G | 0.07 | 0.93 | 0.9 | Intron |
| rs73506160 | *ABCA1* | 9 | 107683841 | A/T | 0.29 | 0.37 | 0.07 | Intron |
| rs2472509 | *ABCA1* | 9 | 107684230 | T/G | 0.13 | 0.1 | 0.36 | Intron |
| rs2472508 | *ABCA1* | 9 | 107684253 | G/A | 0.2 | 0.16 | 0.21 | Intron |
| rs10991411 | *ABCA1* | 9 | 107684276 | T/C | 0.29 | 0.37 | 0.07 | Intron |
| rs2487049 | *ABCA1* | 9 | 107684286 | A/G | 0.14 | 0.09 | 0.2 | Intron |
| rs2515614 | *ABCA1* | 9 | 107684318 | A/C | 0.13 | 0.1 | 0.36 | Intron |
| rs10991412 | *ABCA1* | 9 | 107684405 | G/A | 0.29 | 0.37 | 0.07 | Intron |
| rs10991413 | *ABCA1* | 9 | 107684419 | T/C | 0.09 | 0.11 | 0.1 | Intron |
| rs78880933 | *ABCA1* | 9 | 107684488 | A/G | 0.06 | 0.09 | 0 | Intron |
| rs12343571 | *ABCA1* | 9 | 107684626 | C/A | 0.03 | 0.06 | 0.04 | Intron |
| rs73664382 | *ABCA1* | 9 | 107684848 | G/A | 0.22 | 0.31 | 0.04 | Intron |
| rs73664383 | *ABCA1* | 9 | 107684907 | C/T | 0.08 | 0.11 | 0 | Intron |
| rs73664384 | *ABCA1* | 9 | 107684943 | T/C | 0.23 | 0.31 | 0.04 | Intron |
| rs77386036 | *ABCA1* | 9 | 107685016 | G/A | 0.23 | 0.31 | 0.04 | Intron |
| rs139040057 | *ABCA1* | 9 | 107685155 | G/A | 0.18 | 0.26 | 0 | Intron |
| rs142234285 | *ABCA1* | 9 | 107685156 | C/T | 0.2 | 0.27 | 0 | Intron |
| rs112555145 | *ABCA1* | 9 | 107685200 | A/G | 0.23 | 0.33 | 0.04 | Intron |
| rs73506168 | *ABCA1* | 9 | 107685387 | G/A | 0.25 | 0.36 | 0.0013 | Intron |
| rs2516313 | *ABCA1* | 9 | 107685542 | G/T | 0.39 | 0.45 | 0.37 | Intron |
| rs60295489 | *ABCA1* | 9 | 107685568 | T/C | 0.2 | 0.22 | 0.11 | Intron |
| rs2487050 | *ABCA1* | 9 | 107685577 | C/A | 0.09 | 0.08 | 0.23 | Intron |
| rs10991414 | *ABCA1* | 9 | 107685589 | T/C | 0.32 | 0.41 | 0.14 | Intron |
| rs144441671 | *ABCA1* | 9 | 107685767 | T/C | 0.01 | 0.02 | 0 | Intron |
| rs2515613 | *ABCA1* | 9 | 107686131 | A/G | 0.41 | 0.47 | 0.46 | Intron |
| rs116639139 | *ABCA1* | 9 | 107686148 | T/C | 0.02 | 0.04 | 0 | Intron |
| rs2437821 | *ABCA1* | 9 | 107686241 | T/C | 0.09 | 0.08 | 0.23 | Intron |
| rs2487051 | *ABCA1* | 9 | 107686292 | C/T | 0.09 | 0.08 | 0.23 | Intron |
| rs10991415 | *ABCA1* | 9 | 107686354 | A/G | 0.16 | 0.18 | 0.11 | Intron |
| rs2487052 | *ABCA1* | 9 | 107686405 | C/T | 0.09 | 0.08 | 0.23 | Intron |
| rs76908150 | *ABCA1* | 9 | 107686592 | T/TA | 0.32 | 0.39 | 0.22 | Intron |
| rs2515612 | *ABCA1* | 9 | 107686752 | G/A | 0.09 | 0.08 | 0.23 | Intron |
| rs10820747 | *ABCA1* | 9 | 107686823 | G/A | 0.32 | 0.39 | 0.22 | Intron |
| rs77727599 | *ABCA1* | 9 | 107687017 | T/A | 0.03 | 0.04 | 0 | Intron |
| rs2437820 | *ABCA1* | 9 | 107687033 | C/G | 0.42 | 0.47 | 0.45 | Intron |
| rs2472377 | *ABCA1* | 9 | 107687104 | C/T | 0.42 | 0.47 | 0.45 | Intron |
| rs200931562 | *ABCA1* | 9 | 107687122 | TA/T | 0.01 | 0.02 | 0.02 | Intron |
| rs56042940 | *ABCA1* | 9 | 107687151 | C/T | 0.11 | 0.13 | 0.13 | Intron |
| rs2472507 | *ABCA1* | 9 | 107687193 | A/C | 0.09 | 0.08 | 0.23 | Intron |
| rs55737841 | *ABCA1* | 9 | 107687261 | G/A | 0.11 | 0.13 | 0.13 | Intron |
| rs2487053 | *ABCA1* | 9 | 107687425 | C/T | 0.09 | 0.08 | 0.23 | Intron |
| rs2515611 | *ABCA1* | 9 | 107687496 | G/A | 0.09 | 0.07 | 0.23 | Intron |
| rs10991416 | *ABCA1* | 9 | 107687698 | A/T | 0.19 | 0.22 | 0.11 | Intron |
| rs2472506 | *ABCA1* | 9 | 107687711 | C/T | 0.09 | 0.08 | 0.23 | Intron |
| rs115919518 | *ABCA1* | 9 | 107687896 | C/G | 0.03 | 0.04 | 0 | Intron |
| rs2515610 | *ABCA1* | 9 | 107687906 | G/T | 0.09 | 0.08 | 0.23 | Intron |
| rs2515609 | *ABCA1* | 9 | 107688059 | G/A | 0.09 | 0.08 | 0.21 | Intron |
| rs10991417 | *ABCA1* | 9 | 107688629 | A/C | 0.3 | 0.77 | 0.48 | Intron |
| rs201952658 | *ABCA1* | 9 | 107688755 | A/AC | 0.16 | 0.2 | 0.11 | Intron |
| rs75070349 | *ABCA1* | 9 | 107688764 | A/AC | 0.31 | 0.24 | 0.47 | Intron |
| rs10991418 | *ABCA1* | 9 | 107688770 | C/T | 0.19 | 0.21 | 0.11 | Intron |
| rs62568211 | *ABCA1* | 9 | 107688892 | C/T | 0.08 | 0.12 | 0.12 | Intron |
| rs78248658 | *ABCA1* | 9 | 107688916 | C/CG | 0.02 | 0.01 | 0.11 | Intron |
| rs62568212 | *ABCA1* | 9 | 107689035 | G/T | 0.04 | 0.06 | 0.12 | Intron |
| rs10820749 | *ABCA1* | 9 | 107689042 | C/A | 0.3 | 0.76 | 0.48 | Intron |
| rs4742928 | *ABCA1* | 9 | 107689250 | G/T | 0.32 | 0.76 | 0.48 | Intron |
| rs12347784 | *ABCA1* | 9 | 107689289 | T/G | 0.02 | 0.08 | 0.08 | Intron |
| rs4742929 | *ABCA1* | 9 | 107689352 | A/G | 0.33 | 0.74 | 0.47 | Intron |
| 9.107689493 | *ABCA1* | 9 | 107689493 | G/GA | 0.17 | - | - | Intron |
| rs113598632 | *ABCA1* | 9 | 107689501 | AG/A | 0.42 | 0.41 | 0.56 | Intron |
| rs75193896 | *ABCA1* | 9 | 107689528 | C/A | 0.07 | 0.12 | 0 | Intron |
| rs2472373 | *ABCA1* | 9 | 107689710 | C/G | 0.07 | 0.05 | 0.2 | Intron |
| rs139686313 | *ABCA1* | 9 | 107689802 | T/C | 0.01 | 0.02 | 0 | Intron |
| rs113389093 | *ABCA1* | 9 | 107689815 | A/AC | 0.1 | 0.11 | 0.12 | Intron |
| rs114233414 | *ABCA1* | 9 | 107689829 | T/C | 0.02 | 0.05 | 0 | Intron |
| rs79739357 | *ABCA1* | 9 | 107689865 | G/A | 0.03 | 0.04 | 0 | 5' |
| rs79440945 | *ABCA1* | 9 | 107690039 | G/A | 0.03 | 0.04 | 0 | 5' |
| rs2437817 | *ABCA1* | 9 | 107690057 | C/A | 0.25 | 0.24 | 0.38 | 5' |
| rs2243312 | *ABCA1* | 9 | 107690124 | A/G | 0.25 | 0.23 | 0.38 | 5' |
| rs1800977 | *ABCA1* | 9 | 107690450 | G/A | 0.26 | 0.24 | 0.38 | 5' |
| rs2740483 | *ABCA1* | 9 | 107690535 | G/C | 0.19 | 0.87 | 0.69 | 5' |
| rs5923 | *LCAT* | 16 | 67973953 | G/A | 0.13 | 0.24 | 0.04 | Syn |
| rs114275966 | *LCAT* | 16 | 67974442 | T/G | 0.04 | 0.02 | 0 | Intron |
| rs13336998 | *LCAT* | 16 | 67974893 | G/A | 0.1 | 0.14 | 0 | Intron |
| rs17246758 | *LCAT* | 16 | 67975193 | C/T | 0.01 | 0.02 | 0 | Intron |
| rs13306496 | *LCAT* | 16 | 67976692 | G/T | 0.12 | 0.16 | 0.0026 | Intron |
| rs1109166 | *LCAT* | 16 | 67977382 | T/C | 0.36 | 0.74 | 0.18 | Intron |
| rs11860141 | *LCAT* | 16 | 67977541 | G/C | 0.12 | 0.17 | 0.0013 | Intron |
| rs11860115 | *LCAT* | 16 | 67977696 | A/C | 0.12 | 0.17 | 0.0013 | Intron |

*Abbreviations: Minor Allele Frequency (MAF); ^1^ Build 37 Position; ^2^ MAF among 1000 Genomes Populations (*[*www.1000genomes.org*](http://www.1000genomes.org) *) with predominantly African ancestry (AFR) or European ancestry (EUR).*
